# Genome-Wide Identification and Expression Analysis of Tomato *ADK* Gene Family during Development and Stress

**DOI:** 10.3390/ijms22147708

**Published:** 2021-07-19

**Authors:** Lu Yang, Haohao Cao, Xiaoping Zhang, Liangxian Gui, Qiang Chen, Gui Qian, Jiaxin Xiao, Zhengguo Li

**Affiliations:** 1 Key Laboratory for the Conservation and Utilization of Important Biological Resources, College of Life Sciences, Anhui Normal University, Wuhu 241000, China; yanglu@ahnu.edu.cn (L.Y.); zhxp7463@ahnu.edu.cn (X.Z.); gui18375324776@163.com (L.G.); chenlion0804@163.com (Q.C.); xianyuebaili@gmail.com (G.Q.); 2 Key Laboratory of Plant Hormones and Development Regulation of Chongqing, School of Life Sciences, Chongqing University, Chongqing 400044, China; haohaocao@126.com

**Keywords:** genome-wide, tomato, *ADK* gene family, stress

## Abstract

Adenylate kinase (ADK) is widely distributed in organisms and plays an important role in cellular energy homeostasis. In plants, ADK has important functions in plant growth and development regulation as well as in adaptation to the environment. However, little information is available about the *ADK* genes in tomato (*Solanum lycopersicum*), an important economic crop. To investigate the characteristics and functions of *ADK* genes in tomato, a total of 11 *ADK* genes were identified and named according to their chromosomal locations. The ADK family in Arabidopsis, tomato, potato, and rice was divided into six groups, and motif analysis revealed that each SlADK protein contained five to eight conserved motifs. A total of 4 to 19 exons were identified in tomato *ADK* gene family members, and interestingly, most members possessed 4 exons. Several stress response elements were identified in the promoter regions of *SlADKs*. The 11 *SlADKs* were randomly distributed on 9 of the 12 tomato chromosomes. Three duplication events were observed between tomato chromosomes, and a high degree of conservation of synteny was demonstrated between tomato and potato. The online TomExpress platform prediction revealed that *SlADKs* were expressed in various tissues and organs, basically consistent with the data obtained from real-time quantitative PCR (qPCR). The qPCR verification was also performed to determine the expression level of *SlADKs* and demonstrated that the genes responded to multiple abiotic stresses, such as drought, salt, and cold. Besides, the qPCR results showed that *SlADK* transcription was responsive to most of the applied hormone treatment. For correlation network analysis under 44 global conditions, the results showed that the number of 17, 3, 4, and 6 coexpressed genes matched with *SlADK5*, *8*, *9*, and *11*, respectively. For specific gene function analysis, expression of *SlADK10* was inhibited using virus-induced gene silencing (VIGS). Compared to wild-type plants, plants with silenced *SlADK10* gene had poor drought resistance, indicating *SlADK10* regulated drought tolerance of tomato positively. In summary, the information provided in the present study will be helpful to understand the evolutionary relationship and their roles of tomato *ADK* gene family in further research.

## 1. Introduction

Adenosine monophosphate (AMP) is one of the four main mononucleotides that make up ribonucleic acid in cells, also known as adenylate. Formation of AMP is often accompanied by the release of energy in organisms [1]. Adenylate metabolism is an essential part of primary metabolism because the change of adenylate content is the main factor affecting cell metabolism [2]. AMP, adenosine diphosphate (ADP), and adenosine triphosphate (ATP) are three important adenylate forms in organisms. The ratio of AMP, ADP, and ATP determines the energy charge ratio and carbohydrate metabolism, which directly affect plant growth and development and the ability to resist stress [3,4].

Adenylate kinase (ADK, EC 2.7.4.3) is a ubiquitous and abundant enzyme found in virtually all living organisms [5]. It catalyzes a reversible transphosphorylation reaction (ATP + AMP ↔ 2ADP) and is considered as a crucial enzyme in maintaining energy metabolism and the pool sizes of various adenylates at equilibrium [6,7]. Usually, ADKs have three domains: a large central CORE domain, a nucleoside monophosphate binding domain, and an ATP-binding domain [8,9]. The activity of ADK enzyme has been demonstrated in many plants such as maize, rice, and potato, and the subcellular localization of ADK (cytosol, mitochondria, plastids, etc.) varies greatly in different plants [10,11,12,13]. Potato is a solanaceous plant abundant in starch. A previous study revealed that content of adenylic acid and production of starch were significantly improved after the suppression of *StADK* expression in potato plastids [12]. *Arabidopsis thaliana*, as a good model plant, was widely used for studying plant growth and development. Increased amino acid levels and enhanced root growth were confirmed in Arabidopsis after disruption in one *ADK* gene *At2g37250* with transferred DNA (T-DNA) insertion mutants [13]. Subsequently, another relevant study revealed that disruption of Arabidopsis *ADK* gene *At5g47840* leads to loss of chloroplast integrity, causing a bleached phenotype from early embryo to seedling development [14]. Researchers also found that ADK3 could interact with the chloroplast glyceraldehyde-3-phosphate dehydrogenase to form a stable complex in the chloroplasts of a green alga, which might be a potential mechanism to regulate the crucial ATP–NADPH ratio in the Calvin–Benson cycle [15].

Besides regulation of growth and development, ADK is also widely involved in abiotic stress responses in plants. When roots and stems of maize were treated with solutions of two different ratios of Ca^2+^/Na^+^, results showed that ADK content had an essential relationship with salt stress [16]. In tomato, microarray analysis of genes revealed that an *ADK* homolog (SGN-U214214) was repressed in salt-treated tissues [17]. Other microarray data revealed that the expression of *ADK* gene (SGN-U232826) was induced by drought stress in drought-tolerant tomato [18]. Taking pea seeds as a model, the balance of adenylate in dehydrating and imbibing seeds was investigated. The results indicated that ADK played a crucial role in building and later using the huge AMP pool, which appears as a signature of the dry state in seeds [19].

Tomato is one of the most important agricultural products worldwide, as well as an important model for studying fleshy fruit development and ripening [20]. Currently, the tomato *ADK* gene family members have not been identified, and their functions remain to be elucidated. Due to the importance of the *ADK* genes in regulating plant growth and stress resistance, it would be of interest to make a systematic investigation of the *ADK* family in tomato. In the present study, we used bioinformatics methods to identify *ADK* genes from the tomato genome and analyze the phylogenetic relationships, sequence features, gene location, chromosomal locations, evolutionary relationships, and cis-elements in promoters. The comprehensive transcriptomic profiling of the *ADK* family in various tissues and organs of tomato during different developmental stages were carried out using the online TomExpress platform. Also, the dynamic expression patterns of the *ADK* family in response to various plant hormones (methyl jasmonate (MeJA), ethylene (Eth), salicylic acid (SA), indole 3-acetic acid (IAA), and abscisic acid (ABA)) and abiotic stresses (drought, cold, and salt stress) were systematically studied in detail using quantitative real-time PCR (qRT-PCR). Furthermore, coexpression and correlation networks between *SlADKs* and other tomato functional genes were further investigated. Virus-induced gene silencing technology verified the function of *SlADK10* under drought stress. In brief, the present results will provide useful information for further functional and regulation mechanism investigations of the *ADK* family in tomato.

## 2. Results

### 2.1. Identification of the ADK Family in Tomato

To identify the *ADK* family in tomato, unigenes were searched in the SOL Genomics Network, and a hidden Markov model search was used to scan probable proteins. After confirming the existence of the *ADK* core sequences according to the description in the tomato genome database and the Pfam and SMART programs, a total of 11 *ADK* genes were identified. Based on their chromosomal locations, the genes were assigned as *SlADK1*–*11*. Gene information of each *ADK* gene and physicochemical properties of matched proteins were predicted with the tomato gene database and the ExPASy online tool, respectively. The names and IDs of genes, chromosomal and strand locations, open reading frame lengths, exon numbers, amino acid numbers, molecular weights (MWs), and isoelectric points (pIs) are listed in Table 1. In detail, the lengths of the SlADK proteins ranged from 630 (SlADK1) to 1989 (SlADK11) amino acids, and the corresponding range for MWs was 22872.10–74110.62 Da. The 11 *SlADK* genes were distributed on nine tomato chromosomes, with *SlADK3* and *4* located in forward strands and the others in reverse strands. The predicted pI values of SlADKs ranged from 5.76 (SlADK1) to 8.83 (SlADK4) (Table 1). For subcellular localization prediction based on four different online tools, most SlADK proteins were presumably located in mitochondria (e.g., SlADK2, 3, 4, and 6); SlADK1 and 5 may have been located in cytoplasm; and SlADK8 and 11 may have been located in chloroplast. Interestingly, SlADK9 may have been located in cytoplasm or the nucleus, and SlADK10 may have been located in chloroplast or mitochondria (Table 2). The coding sequence (CDS) and gene sequence of the *SlADK* family are provided in Table S1.

### 2.2. Phylogenetic Analysis and Multiple Sequence Alignment of SlADK Genes

**Figure 1:**
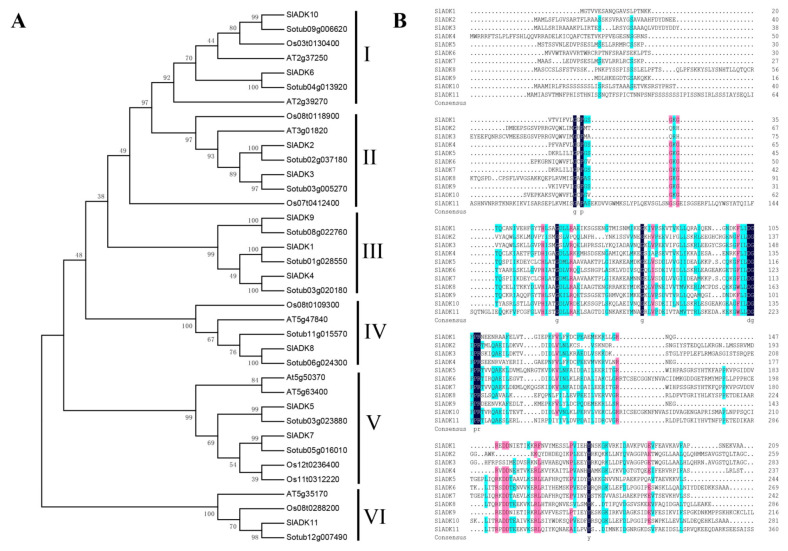
Phylogenetic analysis and multiple sequence alignment of adenylate kinase (ADK). (**A**) Phylogenetic analysis of tomato ADK family members, together with 7 Arabidopsis ADKs, 12 potato ADKs, and 7 rice ADKs. Full length of ADK proteins in these species were aligned using ClustalW and phylogenetic trees was carried out by the neighbor-joining method on MEGA7. Numbers on branches are bootstrap values calculated from 1000 replicates. Each branch is marked out with Roman numerals. (**B**) Multiple sequence alignment of SlADKs. SlADK proteins were aligned using DNAMAN8 software. Highlight homology level in 100%, ≥75% and ≥50% with the colors of blue, pink and green, respectively.

The phylogenetic relationship of tomato SlADK proteins, together with seven Arabidopsis AtADKs, twelve potato StADKs, and seven rice OsADKs, were examined by multiple sequence alignment with full lengths of the amino acid sequences (Table S2). Then, a phylogenetic analysis was conducted by MEGA7 based on the aligned results, and the neighbor-joining method was used with bootstrap replications of 1000. The ADK family was divided into six groups (Figure 1A). SlADK6 and 10, together with two potato homologs (Sotub09g006620 and Sotub04g013920), two Arabidopsis homologs (AT2g39270 and AT2g37250), and one rice homolog (Os03t0130400), were phylogenetically distinct and formed the predicted group I of plant ADKs. Group II contained two isoforms of potato (Sotub02g037180 and Sotub03g005270), one of Arabidopsis (AT3g01820), two of rice (Os08t0118900 and Os07t0412400), and two of tomato (SlADK2 and 3). Group III contained three evolutionarily similar subbranches: SlADK1 and Sotub01g028550; SlADK4 and Sotub03g020180; and SlADK9 and Sotub08g022760. Group IV contained two isoforms of potato (Sotub06g024300 and Sotub11g015570), one of Arabidopsis (At5g47840), one of rice (Os08t0109300), and one of tomato (SlADK8). Group V contained four subbranches: At5g50370 and At5g63400; SlADK5 and Sotub03g023880; SlADK7 and Sotub05g016010; and Os12t0236400 and Os11t0312220. At last, SlADK11, together with Sotub12g00740, Os08t0288200, and AT5g35170, formed group VI. Multiple sequence alignment of SlADK proteins was performed using DNAMAN8 software (Figure 1B). Because the amino acid sequence of SlADK11 was much longer than other proteins, the last comparison part, which contained only ADK11 C-terminal amino acids, is not shown in Figure 1B. 

### 2.3. Motif Analysis

**Figure 2:**
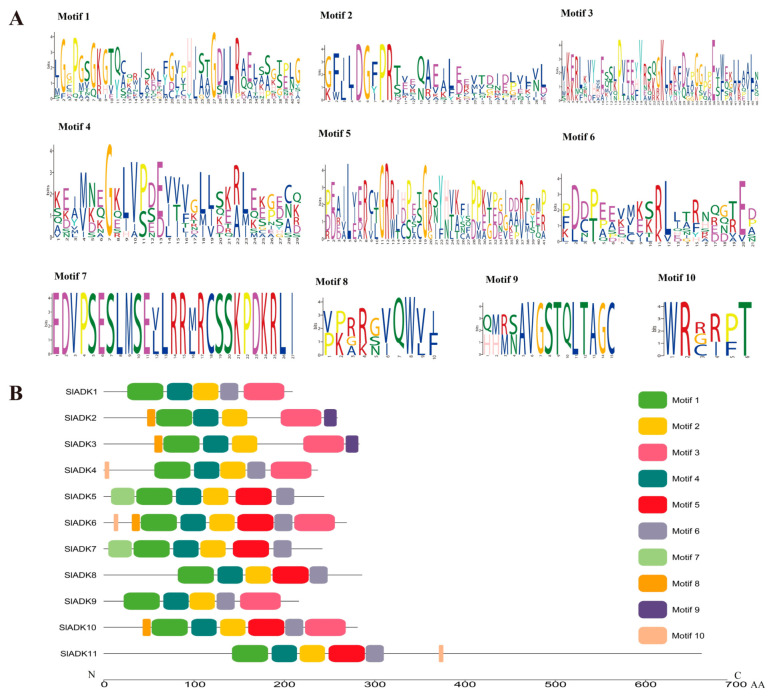
Details of ten conserved motifs and their distributions in SlADK proteins. (**A**) Details of ten selected motifs. Ten conserved motifs in SlADK proteins were generated by the online MEME tool. The overall height of the stack represents the level of sequence conservation. Heights of residues within a stack indicate the frequency of each residue at the indicated position. (**B**) The motif composition of tomato SlADK proteins. Ten putative motifs are indicated in different colored boxes. The length of each protein can be estimated using the scale at the bottom.

**Figure 3:**
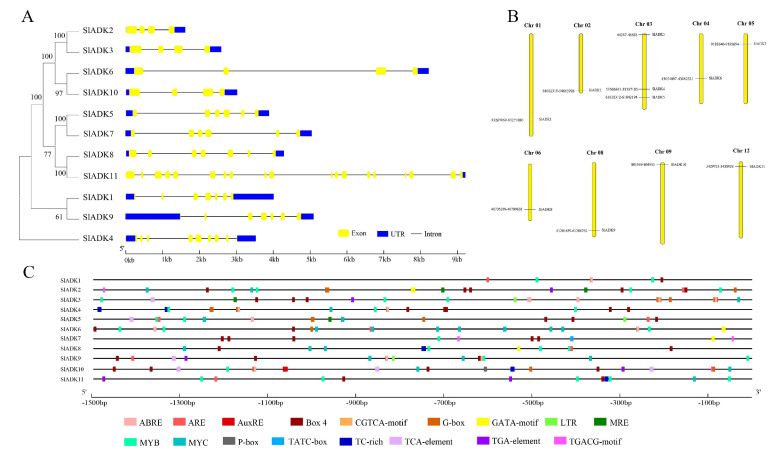
Phylogenetic relationships, gene structures, chromosomal locations, and predicted cis-elements of the *SlADK* gene family. (**A**) Phylogenetic tree (left) of 11 SlADK proteins. The unrooted neighbor-joining phylogenetic tree was constructed with MEGA7 using full-length amino acid sequences of 11 SlADK proteins, and the bootstrap test replicate was set as 1000 times. Exon/intron organization (right) of *SlADK* genes. Yellow boxes represent exons, and black lines with same length represent introns. The UTR region of *SlADK* genes are indicated in blue boxes. The length of exons can be inferred by the scale at the bottom. (**B**) Chromosomal location of *SlADK* genes. The number of chromosomes is indicated at the top of each chromosome. Also, the numbers at the left and right of each chromosome represent genomic locations and names of *SlADK* genes, respectively. (**C**) Predicted stress- and hormone-related cis-elements in *SlADK* promoters. Promoter sequences (−1500 bp) of 11 *SlADK* genes were analyzed by PlantCARE. The upstream length to the translation start site can be inferred according to the scale at the bottom.

According to the amino acid sequences, the MEME web server was used to search the conserved motifs that were shared with the SlADK proteins. A total of 10 distinct conserved motifs were set up and found, and the number of motif residues ranged within 38–50 amino acids; details of the conserved motifs are shown in Figure 2A. Each SlADK protein contained five to eight conserved motifs. It is worth noting that motifs 1, 2, and 4 were fundamental in the ADK domains because they were shared by all SlADKs (Figure 2B). SlADK1 and 9, SlADK2 and 3, and SlADK5 and 7 shared common motif compositions, which was consistent with the grouping results (Figure 1A and Figure 3A left). In addition, motif 7 was unique to SlADK5 and 7 at the N-terminal, and motif 9 was unique to SlADK2 and 3 at the C-terminal. The specific motifs may contribute to the functional divergence of *SlADK* genes. In order to better annotate the function of genes, the sequence of each motif was performed blast in Pfam and SMART database; the prediction result showed that except for motifs 7 to 10, which were too short to predict, motif 1 to 6 all contained the ADK domain (Table S3).

### 2.4. Gene Structure and Chromosomal Location Analysis of SlADKs

Gene organization plays a vital role in the evolution of multiple gene families [21]. A neighbor-joining phylogenetic tree constructed with MEGA7 is shown in Figure 3A, which is consistent with the result in Figure 1A. Corresponding to each gene, the genomic sequence and cDNA sequence information were submitted to the Gene Structure Display Server ((http://gsds.gao-lab.org/) (accessed on 7 August 2019) together to show the gene structure. Among these genes, the average gene length was 1610–9218 bp. *SlADK 11* had the maximum number of exons and the longest gene length, while *SlADK2* had the shortest gene length. Each gene had 4–19 exons, and the majority of *SlADK*s harbored four exons (*SlADK2*, *3*, *6*, and *10*) (Figure 3A right, Table 1). The results also revealed that genes close to each other in the phylogenetic tree had similar gene structure, such as *SlADK2* and *3*, *SlADK6* and *10*, *SlADK5* and *7*, and *SlADK1* and *9*. Tomato contains 12 chromosomes; the 11 *SlADK*s were distributed on 9 of them randomly (Figure 3B). The majority of *SlADK*s were located on the proximate or distal ends of the chromosomes. Chromosome 03 had the greatest number of predicted *SlADK*s, with three (*SlADK3*–*5*), and no *SlADK* existed on chromosomes 07, 10, or 11. Only one *SlADK* existed on each of the other eight chromosomes.

### 2.5. Cis-Regulatory Elements in SlADK Promoters

To pave the way for further study of potential gene function and regulatory mechanisms of the *SlADK* family, especially during abiotic stress responses and hormone treatment, the 1500 bp upstream sequences from the translation start sites of *SlADK*s were submitted to PlantCARE to detect cis-regulatory elements (CREs). After screening, CREs related to stress and hormones were retained; detailed information on these CREs can be found in Table S4. Also, the location and number of 17 representative CREs (ABRE, ARE, AuxRE, Box 4, CGTCA-motif, G-box, GATA-motif, LTR, MRE, MYB, MYC, P-box, TATC-box, TC-rich, TCA-element, TGA-element, TGACG-motif) were visualized on each gene with GSDS software (Figure 3C). The upstream regulatory sequence of promoters contained multiple elements that respond to hormones (such as Box 4 and G-box, TGACG-motif) and stress signals (such as TC-rich, LTR, MYB- and MYC-binding sites), which indicated that expressions of *SlADK*s were associated with abiotic stresses and hormone signal transduction response. It is worth noting that from the promoter region of −1500 bp to −700 bp, cis-regulatory elements of *SlADK11* and *1* seemed to be less distributed and almost nonexistent, respectively (Figure 3C and Table S4).

### 2.6. Synteny Analysis of SlADK Genes

**Figure 4:**
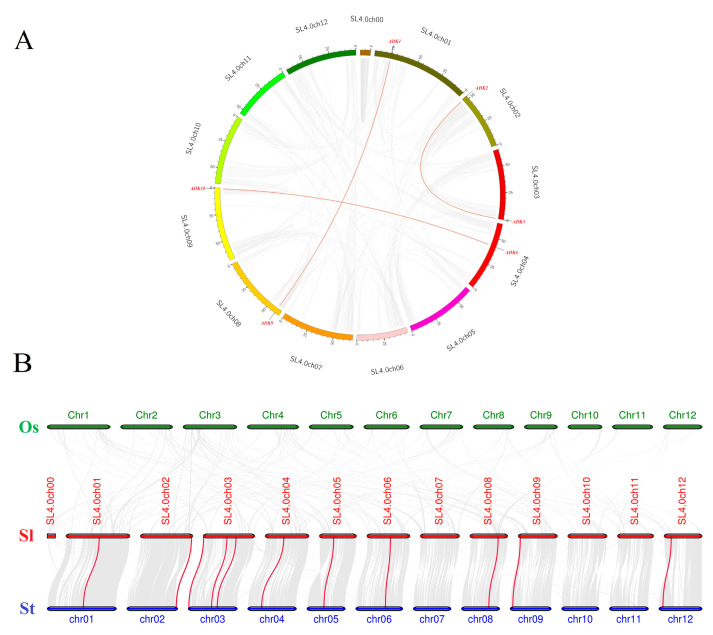
Collinearity analysis of *ADK* gene family. (**A**) Synteny analysis of the *ADK* family in tomato. The gray lines represent the collinearity result of the tomato genome, and red lines linking *SlADK* genes represent the duplication events occurring in the tomato *ADK* gene family. Chromosomes are drawn in different colors, and the approximate location of *SlADK* genes is shown by short black lines on the circle. (**B**) Synteny analysis of *ADK* genes between tomato and other two representative plant species. The gray lines in the background represent the collinear blocks within tomato and other plant genomes (potato, rice), while the red lines highlight the syntenic *ADK* gene pairs. Here the abbreviations “Os”, “Sl”, and “St” indicate *Oryza sativa*, *Solanum lycopersicum*, and *Solanum tuberosum*, respectively.

Synteny analysis of *SlADK* genes was conducted to investigate the duplication events occurring in the tomato *ADK* family (Figure 4A). Three duplication events were observed between chr01 and chr08 (*SlADK1* and *SlADK9*), chr02 and chr03 (*SlADK2* and *SlADK3*), and chr04 and chr09 (*SlADK6* and *SlADK10*), which evolved from segment duplication. Interestingly, each gene pair with duplication events belonged to the same subfamily in the phylogenetic tree (Figure 3A). To further infer the phylogenetic mechanisms of tomato *ADK* gene family, we constructed two comparative syntenic maps of tomato associated with two representative species, rice and potato (Figure 4B). The homology between tomato and potato is closer because they both belong to Solanaceae while rice belongs to Gramineae. Under default parameters (minspan of mcsanx = 30), our results revealed that ten *SlADK* genes showed syntenic relationship with potato genes (*SlADK1* and *Sotub01g028550*, *SlADK2* and *Sotub02g037180*, *SlADK3* and *Sotub03g005270*, *SlADK5* and *Sotub03g023880*, *SlADK6* and *Sotub04g013920*, *SlADK7* and *Sotub05g016010*, *SlADK8* and *Sotub06g024300*, *SlADK9* and *Sotub08g022760*, *SlADK10* and *Sotub09g006620*, *SlADK11* and *Sotub12g007490*); when the parameter minspan of mcsanx was changed from 30 to 12, previously filtered gene pairs of *SlADK4* and *Sotub03g020180* also showed syntenic relationships (Figure 4B). However, no collinear relationship existed for *ADK* genes between rice and tomato (Figure 4B).

### 2.7. Expression Pattern Analysis of SlADKs

**Figure 5:**
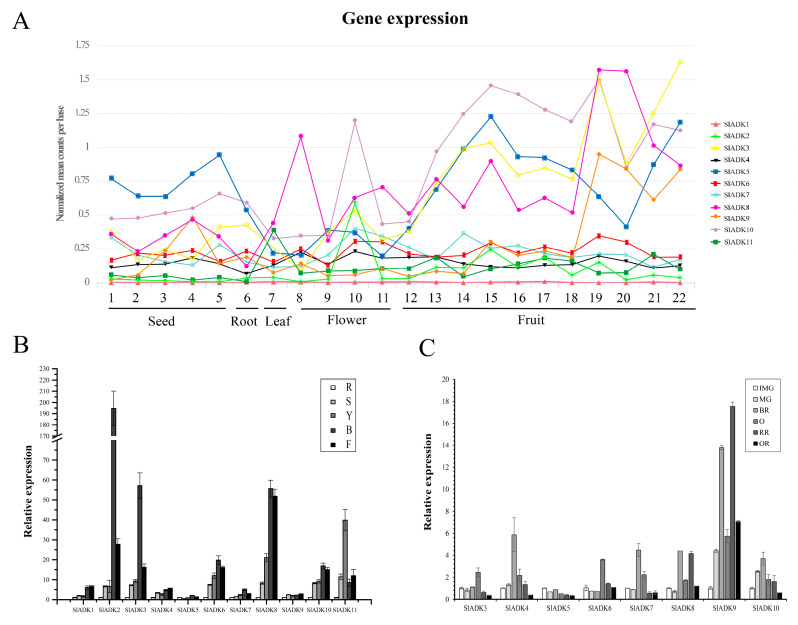
Expression patterns of the *SlADK* genes in different tissues. (**A**) Expression patterns of the *SlADK* genes in various tissues obtained from the TomExpress platform. 1, whole seed of immature green fruit (10 day post anthesis, DPA); 2, whole seed of mature green fruit (35 DPA); 3, whole seed of breaker fruit (38 DPA); 4, whole seed of orange fruit (41 DPA); 5, whole seed of red fruit (44 DPA); 6, whole root; 7, whole leaf; 8, petal of flower; 9, whole flower in the stage of bud; 10, whole flower in the stage of bud at 3 mm; 11, whole flower in the stage of anthesis; 12, whole fruit in the stage of 4 DPA; 13 and 14, flesh and peel of immature green fruit (10 DPA), respectively; 15 and 16, flesh and peel of mature green fruit (35 DPA), respectively; 17 and 18, flesh and peel of breaker fruit (38 DPA), respectively; 19 and 20, flesh and peel of orange fruit (41 DPA), respectively; 21 and 22, flesh and peel of red fruit (44 DPA), respectively. (**B**) Expression of *SlADK* genes with real-time PCR detection in various tissues including roots (R), stems (S), fully expanded leaves (L), bud (B), and flowers (F). (**C**) Expression of *SlADK* genes with real-time PCR detection at different stages during fruit development and ripening: immature green (IMG), mature green (MG), breaker (Br), orange (O), red ripe (R), and over-ripe (OR). Quantitative PCR data represent mean values for three independent biological replicates (*n* = 3).

Comprehensive transcriptomic profiling of 11 *SlADK*s in tomato vegetative and reproductive tissues was carried out using the online TomExpress platform and associated data mining tools (http://gbf.toulouse.inra.fr/tomexpress (accessed on 12 August 2019)) (Figure 5A and Figure S1). Gene *SlADK1* was seldom expressed in all tomato tissues. Genes *SlADK5* and *10* showed higher expression in seed and root than other genes. The expression of *SlADK10* was enhanced at the flowering and fruit stages and reached the highest level at the stages of bud at 3 mm and mature green fruit (35 DPA). The expression of *SlADK5* was enhanced at the onset of fruit development and reached maximum at mature green fruit (35 DPA); however, during ripening, its expression decreased before increasing again during the red fruit stage. Some genes such as *SlADK2* and *6* exhibited relatively moderate expression in all tissues. Of particular interest, *SlADK3*, *8*, and *9* were the most highly expressed during late fruit ripening, displaying a net upregulation at the onset of ripening starting after the mature green stage. This pattern of expression suggests a potential role of these genes in regulating the ripening process.

To assess the potential roles of *SlADK*s throughout tomato development, we conducted detailed quantitative real-time PCR (qRT-PCR) to examine transcription in different tissues (Figure 5B,C). In nonfruit tissues, including root, stem, leaf, bud, and flower, *SlADK1* and *4* showed similar expression patterns. Notably, the expression levels of most *SlADK*s was drastically enhanced in bud compared to other tissues, and expression of *SlADK2* was much higher in bud. Genes *SlADK2*, *3*, *6*, *7*, *8*, and *10* also showed similar expression patterns, possibly indicating they have similar gene function. Interestingly, the expression of *SlADK11* was higher in leaves than other tissues. Additionally, expression of *SlADK5* and *9* showed little difference among tissues (Figure 5B). During the critical stages of fruit development, including the immature green stage (IMG), mature green stage (MG), breaker stage (BR), orange stage (O), red ripe stage (RR) and overripe stage (OR), the expression levels of *SlADK4*, *7*, and *10* were relatively high at BR stage and those of *SlADK3* and *6* were relatively high at O stage. It was remarkable that the mRNA level of *SlADK9* was significantly upregulated at RR stage, and interestingly, expression of almost all detected genes was relatively low in OR stage. Additionally, low expression of *SlADK1*, *2*, and *11* genes was observed in fruit impeded analysis of gene expression by qPCR (Figure 5C).

### 2.8. Expression Characteristics of SlADKs under Diverse Abiotic Stresses

**Figure 6:**
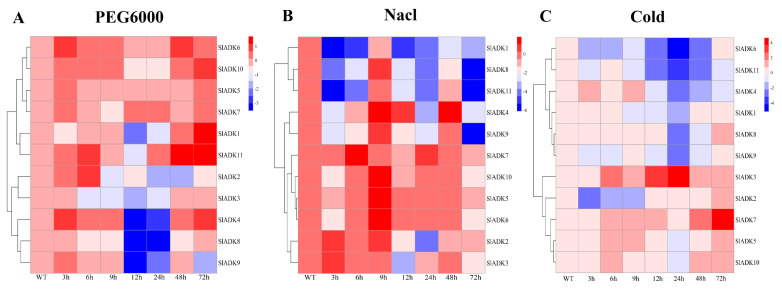
qRT-PCR expression analysis for 11 *SlADKs* in tomato leaves under diverse abiotic stresses: (**A**) PEG6000; (**B**) NaCl; (**C**) cold. The treated tomato leaves were harvested at the indicated time points. The color scale represents the log2 mean value of relative expression levels from three independent biological replicas (*n* = 3). Used primers specific for *SlADK*s are listed in Supplementary Materials Table S5.

To identify potential functions of *SlADK*s in response to different abiotic stresses, their transcript profiles were assayed under drought, salt, and cold treatments (Figure 6). Following 3 h of treatment with PEG6000, the expression of most *SlADK*s was obviously upregulated except for *SlADK1*, *3*, and *8*. Interestingly, expression of most *SlADK*s decreased rapidly during 9–12 h after treatment but increased rapidly during 24–48 h. In particular, the transcript levels of *SlADK1* and *11* almost linearly increased with time under PEG6000 treatment during 12–72 h (Figure 6A). Under salt treatment, expression levels of most *SlADK*s were obviously downregulated at the early stage. It should be noted that the expression of almost all *SlADK*s at 9 and 48 h were higher than that at other time points, whereas only *SlADK7* showed the highest expression at 6 and 24 h (Figure 6B). Under cold stress, there was obvious up- and downregulation in expression levels of *SlADK3* during 12–24 h and 24–48 h, respectively. For *SlADK7*, expression showed no significant differences for all time points before 24 h of treatment, but expression rose rapidly during 24–72 h (Figure 6C). Notably, *SlADK7* exhibited unique changes under multiple stress treatments, suggesting that it may have a unique role in stress responsiveness. Moreover, the result of gene clustering showed that homologous genes, such as *SlADK2* and *3* or *SlADK5* and *7*, always had similar expression patterns, especially under PEG6000 treatment (Figure 3A left and Figure 6A).

### 2.9. Expression Profiles of SlADKs in Response to Diverse Hormone Treatments

**Figure 7:**
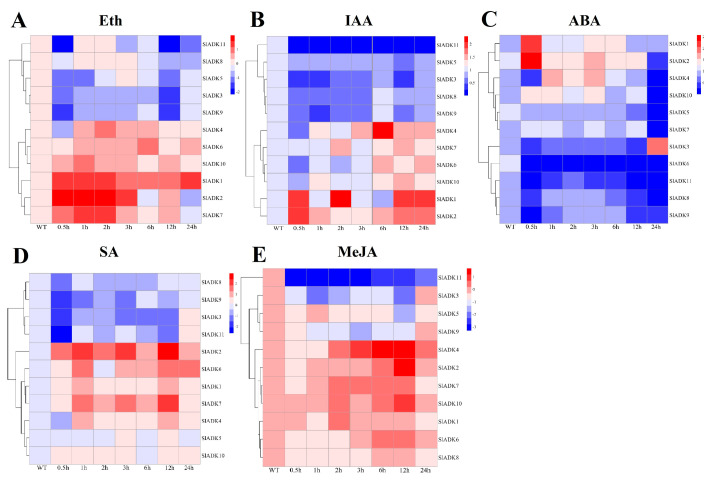
Expression profiles of *SlADKs* under various hormone treatments by qRT-PCR. The treated tomato leaves were harvested at the indicated time points. To ensure that expression of the same gene in different treatments, as well as expression of different genes in the same treatment, can both be clearly displayed and compared, for treatment with Eth (**A**), SA (**D**), and MeJA (**E**), the color scale represents log2 mean value of relative expression levels from three independent biological replicas (*n* = 3); for treatment with IAA (**B**) and ABA (**C**), the color scale represents mean value of relative expression levels from three independent biological replicas (*n* = 3). Primers specific for *SlADK*s are listed in Supplementary Materials Table S5.

Previous evidence indicated that different hormones play important roles in stress signal transduction and cell responses [22,23,24]. Here, we investigated the expression profiles of *SlADK*s in response to Eth, IAA, ABA, SA, and MeJA treatments (Figure 7). With Eth treatment, in general, the expression of most *SlADK*s showed little difference, but that of *SlADK1*, *2*, and *7* increased gradually to different levels during 0–2 h (Figure 7A). With IAA treatment, the expression maxima of *SlADK1*, *2*, and *4* were at 2, 0.5, and 6 h, respectively. Interestingly, *SlADK11* showed decreased transcription at the early stage and almost no expression at later time points (Figure 7B). At 0.5 h after ABA treatment, expression of *SlADK5*, *7*, and *10* showed no significant differences; however, expression of *SlADK3*, *4*, *6*, *8*, *9*, and *11* decreased, and that of *SlADK1* and *2* increased at the first time point. Moreover, most *SlADK*s did not change significantly after 1–12 h of treatment. *SlADK6* showed decreased transcription at the early stage and almost none at following time points (Figure 7C). With SA treatment, transcription of most *SlADK*s was repressed in the first 0.5 h. Expression of most *SlADK*s showed mild change under SA treatment, except for *SlADK2*, *6*, and *7*. *SlADK2* and *7* showed especially similar expression patterns throughout all time points, with maximum expression at 12 h (Figure 7D). The plant regulator MeJA mediates diverse developmental processes and defense responses. It rapidly induced upregulation of *SlADK4* and *2* during 1–6 h and 3–12 h time points, respectively. Expression of *SlADK6* increased gradually during 1–6 h, with opposite results for expression of *SlADK9* during 0–3 h. As in IAA treatment, *SlADK11* showed decreased transcription at the early stage and almost no expression for later time points (Figure 7E). Hence, transcription of *SlADK*s was responsive to most of the applied stress treatments. Interestingly, *SlADK2* and *4* exhibited significant changes under multiple hormone treatments, suggesting that they may have unique roles in hormone regulation. Notably, the result of gene clustering showed that the homologous genes of *SlADK6* and *10* had similar expression patterns under treatment with Eth and IAA (Figure 3A left and Figure 7A,B), and *SlADK8* and *11* had similar expression patterns under treatment with Eth and ABA (Figure 3A left and Figure 7A,C).

### 2.10. SlADK Expression Patterns under Stress Based on RNA-Seq Data

**Figure 8:**
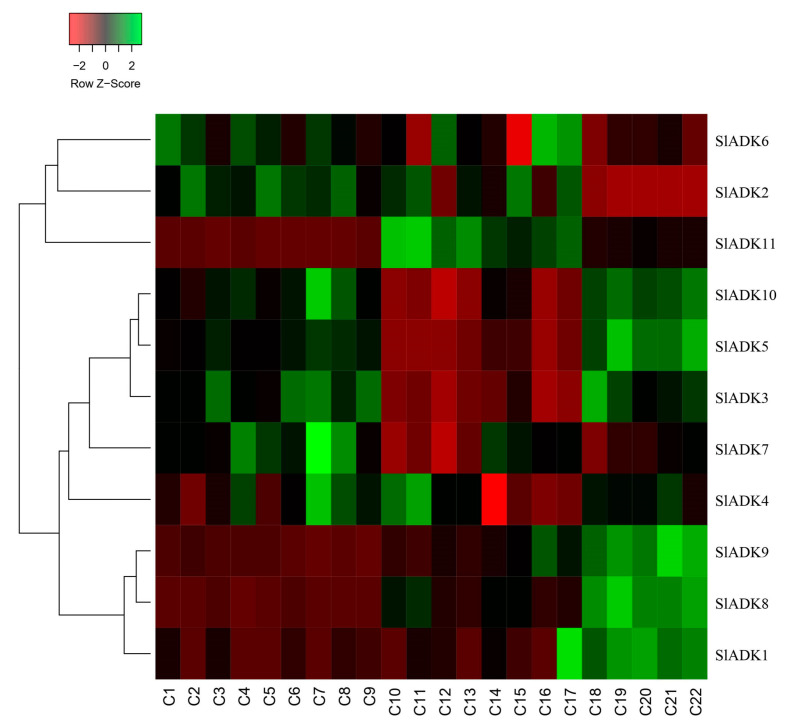
Expression patterns of *SlADK* genes under stress based on RNA-seq data. Heatmap of the expression pattern of tomato *SlADK* family genes in root, leaves and fruit with different and multiple hormones treatment. The distance used for the clustering is based on the classical Euclidean distance which allows clustering gene expression by expression levels. For a given row of the heatmap, green and red colors present high and low values of expression of the considered gene, respectively. Details of C1 to C22 are provided in Figure S2.

As introduced above, TomExpress provides a unified and standard method to judge tomato gene expression from released RNA-Seq data sets. Here, *SlADK*s expression patterns under treatment with different plant hormone and multiple hormones were analyzed (Figure 8 and Figure S2). In general, the expression of most *SlADK*s (*SlADK3*, *5*, *6*, *7*, and *10*) were lower in leaves (C10 to C17) than in roots (C1–C2, C4–C5, C7–C8) whether these tissues were treated with cytokinin or not. Also, the expression of *SlADK1* was lower in many tissues, and even could not be detected in roots (C2, C4, C5, C7) and leaves (C10, C13, C16) under special stress conditions. With the treatment of auxin in tip of roots, the expression of *SlADK7* and *10* were downregulated significantly. In fruit, several *SlADK* genes such as *SlADK3*, *5*, *8*, and *10* displayed higher expression levels. Compared with treatment of ACC in fruit, multiple-hormone treatment of ACC + IAA could upregulate the expression of *SlADK3* and *5* (Figure S2). Heatmap can be very convenient to show gene clustering. As shown in Figure 8, under treatment with different plant hormone and multiple hormones, *SlADK3*, *4*, *5*, *7*, and *10* showed similar expression patterns in many tissues, as did the groups of *SlADK2*, *6*, and *11* and of *SlADK1*, *8*, and *9* (Figure 8).

### 2.11. Coexpression and Correlation Network Analysis

**Figure 9:**
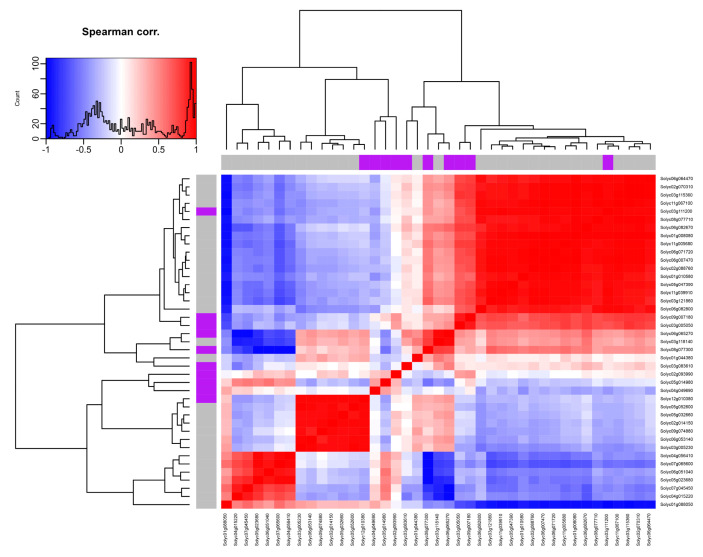
Correction heatmap of *SlADK* genes. Based on the calculation of the correlation values, correlation gene pairs were visualized as a heatmap after a hierarchical clustering to highlight the positively and negatively correlated groups. For a given row of the heatmap, red and blue colors represent positive and negative correlation coefficients, respectively. Purple colors represent *SlADK* family members. The diagonal line from the lower left corner to the upper right corner of the heat map shows the same gene, and the default correlation coefficient is 1.

Based on expression data of *SlADK*s under 44 global conditions of different development and stress treatment derived from TomExpress platform, coexpression and correlation networks were analyzed (Table S6 and Figure 9). The correlation values of coexpressed genes pairs were calculated and the correlation threshold was set as 0.92. Then, the pairs of coexpressed genes of which the correlation coefficient was more than |0.92| were displayed (Table S6). The results showed that *SlADK5*, *8*, *9*, and *11* possessed the numbers of 17, 3, 4, and 6 coexpressed genes, respectively. Among the 17 genes existed correlation with *SlADK5*, 16 showed positive correlation and 1 showed negative correlation; among the 3 genes existed correlation with *SlADK8*, one showed positive correlation and two showed negative correlation. However, the six coexpressed genes of SlADK11 and the four coexpressed genes of *SlADK9* showed only positive and negative correlation, respectively. Furthermore, these correlation data were visualized as a heatmap after a hierarchical clustering to highlight the positively and negatively correlated groups. The results show that *SlADK8* (*Solyc06g065270*) and *9* (*Solyc08g077300*) showed similar coexpression patterns, while *SlADK5* (*Solyc03g111200*) and *11* (*Solyc12g010380*) showed quite different coexpression patterns (Figure 9).

### 2.12. Functional Analysis of SlADK10 Silencing in Tomato under Drought Stress

**Figure 10:**
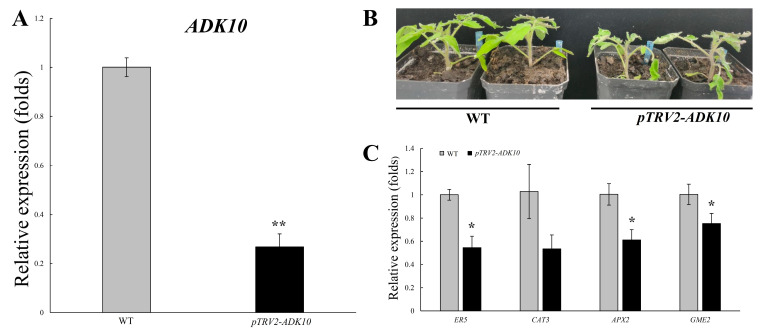
Suppression of *SlADK10* leads to drought sensitivity in tomato. (**A**) Detection of *SlADK* gene expression after virus-induced gene silencing (VIGS). (**B**) Phenotype of TRV2-*ADK10* and wild-type plants under PEG6000 treatment for 24 h. *pTRV2*-*ADK10* showed more obvious wilting symptoms under simulated drought conditions. (**C**) The qRT-PCR analysis of the plant stress-related genes (*ER5*, *CAT3*, *APX2*, *GME2*) in *ADK10*-silenced and WT plants under PEG6000 treatment for 24 h. From three independent biological replicas, data are represented as mean value ± standard error. “*” indicates significant differences between *pTRV2*-*ADK10* and WT plants with *p* < 0.05, as determined by Student’s *t*-test.

Previous microarray data revealed that the expression of an *ADK* gene (SGN-U232826) in drought-tolerant tomato was induced by drought stress [18]. The sequence of SGN-U232826 was consistent with the *SlADK10* identified in our study, which was induced during 24–72 h following PEG6000 treatment (Figure 6). Therefore, *SlADK10* was chosen for further investigation with the virus-induced gene silencing method to further verify gene function. After about 7–10 days following agrobacterium infection, the new leaves of the TRV2-*Su* plants showed yellowing, which indicated that the inoculated plants had systemic spread of the virus (Figure S3). Although the gene of *SlADK10* was silenced, there was no significant growth difference between *pTRV2*-*SlADK10* and WT plants under normal conditions (Figure S3). After one month of tomato inoculation, real-time PCR was performed to detect *SlADK10* gene expression. The results showed the expression of *SlADK10* was significantly reduced in TRV2-*SlADK10* plants compared to that in WT (Figure 10A). Then, phenotypic analysis was performed in *SlADK10*-silenced and WT plants with uniform height under PEG6000 treatment, which was used for simulating drought stress. After 24 h treatment, *SlADK10*-silenced plants showed obvious wilting (Figure 10B). To explore the molecular mechanisms of drought stress resistance mediated by *SlADK10*, transcripts of the plant stress-related genes, including the ethylene-responsive gene *ER5*, ascorbate peroxidase gene *APX2*, GDP-mannose 3′,5′-epimerase gene *GME2*, and catalase gene *CAT3*, were analyzed by qRT-PCR. Consistent with phenotype, the expression of these four genes in TRV2-*SlADK10* plants was lower than that in WT plants after PEG6000 treatment for 24 h (Figure 10C). These results indicated that *SlADK10*-silenced plants were more damaged than WT plants under drought conditions.

## 3. Discussion

ADK is ubiquitous in the kingdoms Animalia and Plantae, and it is found in the cytosol as well as many other organelles such as mitochondria and chloroplasts. So far, ADK-encoding genes have been cloned from a wide variety of plant species [13]. However, genome-wide analysis of the *ADK* gene family has not been pursued in tomato, a model plant for studying plant fruit ripening. In the current study, 11 *SlADK*s in tomato were identified and designated *SlADK1–11* on the basis of their chromosomal location (Table 1). The phylogeny, motif, gene structure, chromosomal location, cis-elements, and expression patterns in different tissues and under stress treatments were analyzed. Synteny analysis was also performed in genome of tomato and among several related plant species. Combined with expression data sets from TomExpress platform, coexpression and correlation networks were investigated between *SlADK*s and other tomato functional genes. This study provides comprehensive information on the *SlADK* family and will aid understanding of the function of *SlADK*s.

Previous research revealed seven *ADK* isoforms with high sequence homology in the Arabidopsis genome [14]. The lower number of *ADK* genes in Arabidopsis may be related to its small genome [25]. Potato belongs to Solanaceae and has strong homology with tomato. In potato, 12 *ADK* genes were identified. Rice, on the other hand, belongs to Gramineae, which contains 7 *ADK* genes (Table S2). The ADK proteins from the four plant species were classified into six groups, with genes in the same group showing a closer evolutionary relationship. For example, SlADK6 and 10 belonged to group I; SlADK2 and 3 belonged to group II; and SlADK1, 4, and 9 belonged to group III. It is worth noting that SlADK1, 4, and 9 were already identified as being closely related to UMP-CMP kinases, with highest homology to the respective three genes in Arabidopsis. Much closer evolutionary relationships existed in the same subbranch. Interestingly, each ADK family member of tomato has a potato ADK family member with high homology, such as SlADK1 with Sotub01g028550, SlADK2 with Sotub02g037180, and so on (Figure 1A). Subcellular localization prediction showed that SlADKs were distributed in the mitochondria, chloroplasts, and other plastids in cells, with the greatest occurrence in chloroplasts (Table 2). This is consistent with previous reports that ADK activity in plants is mainly distributed in the chloroplast matrix and mitochondrial membrane space [26,27,28].

Motif analysis revealed a total of 10 motifs (Figure 2A), with motifs 1, 2, and 4 shared by all SlADKs. In addition, motif 7 was unique to SlADK5 and 7 at the N-terminal, and motif 9 was unique to SlADK2 and 3 at the C-terminal (Figure 2B). Common motifs imply functional redundancy, and the specific motifs may contribute to functional divergence [29]. For the evolution of multiple gene families, the model of gene organization is very important [21]. Gene structure analysis revealed 4–19 exons in each *SlADK* (Figure 3A). All aforementioned genes in the same group (Figure 1A and Figure 3A left), such as SlADK1 and 9, SlADK2 and 3, and SlADK5 and 7, shared common motif compositions (Figure 2B) and similar gene structure (Figure 3A right). This correlation between gene structure and motif arrangement further confirmed the classifications of the *SlADK*s.

A total of 17 CREs related to hormone regulation and stress response were analyzed (Table S4 and Figure 3C). When plants are exposed to abiotic stresses such as salt, drought, or low temperature, ABA-dependent and -independent pathways are simultaneously activated [30,31]. Genes involved in the ABA-dependent pathway not only induce ABA biosynthesis, but also regulate the expression of genes containing ABREs [32,33]. The ABREs mainly occurred in *SlADK3* and *6*, and the G-box element was mostly distributed in *SlADK3* (Table S4 and Figure 3C). In the barley *HVA22* gene and the *Lea* gene promoter, the core sequence ACGT of G-box and other regulatory sequences (CE1 and CE3) constitutes an ABA response complex to facilitate the transcription strength regulation of ABA-regulated genes [34]. The MYB elements are found in the promoters of several stress-resistance genes in Arabidopsis [35]. Our results showed MYB elements distributed in all *SlADK*s, especially *SlADK2* and *3* (Figure 3C). The MYC element is a cis-acting element in response to drought, and ABA and exists in a variety of antistress gene promoters, with reports related to soybeans and Arabidopsis [36,37]. Our results revealed that MYC existed in almost all *SlADK*s, except *SlADK1* and *7*, and was distributed frequently in *SlADK6* (Figure 3C).

Gene duplications play an important role in the evolution of plant genomes and genetic systems [38]. Duplicated genes promote the generation of new genes and their corresponding new functions. Three principal evolutionary patterns are segmental duplication, tandem duplication, and transposition events; the former two patterns can often lead to gene family expansion [39,40]. Our results revealed that in the tomato genome, three duplication events were observed between chromosomes, which evolved from segment duplication (Figure 4A). Compared with other related species, all *SlADK* genes showed syntenic relationships with potato *StADK* genes (Figure 4B). It was found that gene pairs existed collinear relationships were also close to each other in the phylogenetic tree (Figure 1A and Figure 4B).

The expression patterns of *ADK* genes in different tissues have been described in many species, including Arabidopsis [14] and rice [10]. In Arabidopsis, expression of *AtADK*s was detected in leaves, roots, and 16 d old seedlings, and *AtADK1–5* were much more expressed than *AtADK6*, while *AtADK7* was at the detection limit [14]. However, there was no uniform gene expression pattern for *SlADK*s in tomato. Our qPCR results for *SlADK*s in tomato were basically consistent with those predicted by the online TomExpress platform. For example, the predicted expression peak of *SlADK2* and *11* was in bud of 3 mm and leaves, respectively, which was highly consistent with the qPCR result (Figure 5A,B). Also, it was difficult to get satisfactory qPCR results for gene expression analysis of *SlADK1*, *2*, and *11* due to their low expression abundance and amplification efficiency, which was consistent with the software prediction that these three genes had very low expression levels in fruit (Figure 5A,C). For qPCR detection, *SlADK2*, *3*, *6*, *7*, *8*, and *10* also showed similar expression patterns, which hints at similarities in structures, redundancies in functions, and shared induction mechanisms (Figure 5B).

Abiotic stresses, such as drought, high salinity, extreme temperature, and flooding, are major causes of crop loss worldwide, reducing average yields for most major crop plants by more than 50% [41]. In addition to regulation of growth and development, a previous study showed that *ADK*s are widely involved in abiotic stress response in plants [16,17,18,19]. In our study, transcript profiles of the tomato *ADK* family were assayed under drought, salt, and cold treatments (Figure 6). With the increased time of PEG6000 treatment, expression of most *SlADK*s was upregulated, especially of *SlADK1* and *11*. Microarray data revealed that the expression of an *ADK* gene (SGN-U232826) in drought-tolerant tomato was induced by drought stress [18]. The sequence of SGN-U232826 was consistent with the *SlADK10* identified in our study, which was induced during 24–72 h following PEG6000 treatment (Figure 6A). Further functional analysis of *SlADK10* with the VIGS method indicated that *SlADK10*-silenced plants were more damaged than the WT plants under drought conditions (Figure 10B). The ethylene-responsive gene *ER5*, ascorbate peroxidase gene *APX2*, GDP-mannose 3′,5′-epimerase gene *GME2*, and catalase gene *CAT3* were plant stress-related genes [42,43,44]. When transgenic plants become more resistant to drought, the expression of these genes is upregulated in general [45]. Here, after PEG6000 treatment for 24 h, transcript levels of *ER5*, *CAT3*, *APX2*, and *GME2* were downregulated in *SlADK10*-silenced plants compared to those in WT plants, which further confirmed the positive regulatory role of *SlADK10* in drought response. The maintenance of mitochondrial ATP synthesis during water stress is essential for preserving plastid function, and increased ADK gene expression may indicate the ability to provide more ATP for maintaining cellular activities under drought stress [18,46]. With PEG6000 treatment, gene clustering results showed that *SlADK6* and *10*, *SlADK5* and *7*, and *SlADK2* and *3* possessed similar expression patterns, which supports the gene sequence homology (Figure 1A, Figure 3A left, and Figure 6A). Several enzymes, such as ADK and catalase, were specially induced by drought but repressed under salt stress in tomato [17]. In tomato, microarray analysis of genes revealed that an ADK homolog (SGN-U214214), which is the same gene of *SlADK10* named here, was repressed in salt-treated tissues [17]. In the present study, with NaCl treatment, almost all *SlADK*s contained two expression maxima at 9 and 48 h, while the two expression maxima for *SlADK7* were at 6 and 24 h. Gene clustering results showed that the pairs of *SlADK2* and *3* and of *SlADK8* and *11* possessed similar expression patterns, which supports the gene sequence homology (Figure 1A, Figure 3A left, and Figure 6B). Interestingly, with cold treatment, *SlADK6* and *10* displayed quite different expression patterns although their sequences were highly homologous. In addition, *SlADK3* and *7* responded strongly to cold treatment, indicating that they may play a role in cold stress (Figure 6C).

Previous evidence indicated that different hormones play important roles in cell responses and stress signal transduction [22,23,24]. For example, IAA is involved in almost all aspects of plant growth and development, from embryogenesis to senescence, from root tip to shoot tip [47]. Eth is a key regulator during fleshy fruit ripening [48]. ABA is a crucial phytohormone induced by biotic or abiotic stress and plays important roles in plant tolerance to abiotic stresses [49]. MeJA plays an important role in alleviating biotic (pathogens and insects) and abiotic stresses in plants [50]. Our study showed that the transcripts of these *SlADK*s were responsive to most hormone treatments (Figure 7 and Figure 8). In plants, many hormones need to cross function. For example, two plant hormones, ABA and Eth, play an important role in the complex story of abiotic stress and, consequently, cross-talk between these two has been reported [51]. Also, both Eth and SA play important roles in response to biotic stresses [52]. Notably, *SlADK2* and *4* exhibited significant changes under these hormone treatments, suggesting that they have unique roles in hormone responsiveness (Figure 7). Understanding the response of *SlADK*s to hormones can lay a foundation for further elucidating their functions in plant growth and stress response.

Gene coexpression network analysis (GCNA) is a genetic approach for investigating correlations between genes using large-scale gene expression profiling data, which is especially useful for finding relationships between phenotypic traits and functional modules [53,54]. Besides many ribosomal proteins having high-correlation relationships with *SlADK* under 44 global conditions, other proteins such as threonine-protein kinase and fructose-1,6-bisphosphatase also possessed higher correlation with *SlADK* family members. A high positive correlation coefficient (0.92) existed between *SlADK8* and one resistance protein (Nbs-lrr), which hinted that *SlADK8* may have a potential role in stress resistance (Table S6 and Figure 9).

## 4. Methods

### 4.1. Plant Materials and Growth Conditions

*Solanum lycopersicum* cv. Micro-Tom was used as wild-type plant material. The plant samples used in this study were collected from the Key Laboratory of Plant Hormones and Development Regulation of Chongqing, School of Life Sciences, Chongqing University, Chongqing, China. Collection of plant materials complied with institutional, national, and international guidelines. No specific permits were required. To assess potential roles of *SlADK* family genes throughout tomato development by experiments, the tissues of roots (R); stems (S); leaves (L); bud (B); flowers (F); immature green fruit (IMG); mature green fruit (MG); breaker stage fruit (BR); orange fruit (O); red fruit (R); and over-ripe fruit (OR) were collected from wild-type tomato, frozen in liquid nitrogen immediately, and stored at −80 °C for RNA extraction later. For stress and hormone treatment, germinated seeds were cultivated in a greenhouse with suitable conditions: 16/8 h light/dark cycle, 25/18 °C day/night temperature, 80% relative humidity, and 250 μmol/m^2^/s light intensity. The seeds and subsequently growing plants were watered daily. In addition, the plants were irrigated with nutrient solution once per week. One-month old tomato plants with good growth status were selected and transplanted from soil to hydroponic box for hydroponic culture (pH 5.8), and the Hogland nutrient solution was renewed regularly. Hydroponics were adapted for 5–6 days to eliminate the damage caused by transplantation; then, abiotic stress and hormone treatment experiments were performed with these plants.

### 4.2. Identification of Tomato ADK Genes

Two methods were employed to comprehensively identify tomato *ADK* family genes. In the first method, “adenylate kinase” was used as keywords to retrieve unigene families in the SOL Genomics Network (http://solgenomics.net (accessed on 10 July 2019)) [55]. In the second method, tomato genome information files (gff, cds, pep, fasta suffix files) were downloaded from the Ensembl database (http://plants.ensembl.org/index.html (accessed on 16 July 2019)) and unzipped. Subsequently, corresponding relationships between gene and mRNA were acquired with appropriate script under the bio-linux operating system. The hidden Markov model (HMM) file corresponding to the ADK domain (PF00406) was then downloaded from the Pfam protein family database (http://pfam.sanger.ac.uk/(accessed on 16 July 2019)) [56]. Next, the command of “Hmmsearch” was used to search the *ADK* genes from a tomato genome database under the bio-linux operating system. The default parameters were adopted, and the cutoff value was set to 0.01 [57]. All candidate genes that may have contained an ADK domain based on HMMER results were further examined by confirming the existence of the ADK core sequences using the Pfam and SMART program [58]. In short, the tomato *ADK* gene family was identified with the two aforementioned methods, and then the identified members were further verified combined with gene description in the SOL Genomics Network and gene sequence blast in the National Center for Biotechnology Information (NCBI) database.

### 4.3. Characteristics, Phylogenetic Relationships, and Sequence Analysis of ADK Proteins

In regard to other species, twelve *ADK* family genes in potato were also identified according to the above methods. In rice, seven *ADK* family genes were screened in the China Rice Data Center (http://www.ricedata.cn/gene/ (accessed on 9 October 2020)) and RGAP (http://rice.plantbiology.msu.edu/ (accessed on 9 October 2020)). Also, seven adenylate kinase isoforms were found with high sequence homology in Arabidopsis genome [14]. The full-length ADK proteins in Arabidopsis and identified in tomato, potato, and rice were aligned using ClustalW. Phylogenetic analysis of ADK proteins was performed using MEGA 7.0.26 with the neighbor-joining (NJ) method based on the Poisson model [59], the bootstrap method was used to test the tree with 1000 replicates, and paired deletion was performed [60]. Multiple sequence alignments of the amino acid sequences of tomato ADK proteins were analyzed by DNAMAN8 software (v.8.0, Lynnon Biosoft). The MEME online program (http://meme.nbcr.net/meme/intro.html (accessed on 14 August 2019)) provides a unified portal for online discovery and analysis of sequence motifs representing features, such as protein interaction domains [61]. Here, the MEME Suite Web server (version 4.12.0) for protein sequence analysis was used to identify conserved motifs in the identified tomato ADK proteins, with the number of found motifs as ten and the other parameters as default values. Then, sequences of the motifs were blast in the Pfam (http://www.pfam.org/ (accessed on 14 August 2019)) and SMART (http://smart.embl.de/ (accessed on 14 August 2019)) databases. The ADK protein sequences in tomato were analyzed by the online ProtParam tool of ExPASy (http://weB.expasy.org/protparam/ (accessed on 15 August 2019)) for physical and chemical characteristics. The prediction parameters included the number of amino acids, molecular weight (MW), and theoretical pI [62]. For predicting subcellular localization of mature proteins, four online tools were also employed: CELLO (http://cello.life.nctu.edu.tw/ (accessed on 27 October 2020)), Wolf Psort (http://www.genscript.com/psort/wolf_psort.html (accessed on 27 October 2020)), Predotar (https://urgi.versailles.inra.fr/Tools/Predotar (accessed on 27 October 2020)), and TargetP (http://www.cbs.dtu.dk/services/TargetP/ (accessed on 27 October 2020)).

### 4.4. Gene Structure, Chromosome Location, and CREs of SlADK Genes

The visualization of gene structure and annotation features helps to determine the function and evolution of family genes intuitively. Physical locations of all *SlADK* genes on each chromosome were obtained from a tomato genome database: SOL Genomics Network (http://solgenomics.net (accessed on 17 July 2019)). The exon–intron structure of each *SlADK* was determined by aligning the full-length cDNA sequence with the genomic DNA sequence. The Gene Structure Display Server 2.0 (GSDS2.0; http://gsds.gao-lab.org/ (accessed on 7 August 2019)) program was used to display the gene structures on the basis of the coding sequences (exons), introns, and untranslated region (UTR) composition information [63]. With the strength of the genome annotation, MapChart software was used for mapping the genomic location and relative distances of SlADK genes in the chromosome [64]. The 1500 bp upstream sequences of the *SlADK*-coding sequences were retrieved from the SOL Genomics Network and then submitted to PlantCARE (http://bioinformatics.psb.ugent.be/webtools/plantcare/html/ (accessed on 9 August 2019)) to identify stress- and hormone-related cis-regulatory elements (CREs) [65].

### 4.5. Synteny Analysis of SlADK Genes

For analysis of interchromosomal relationships of *SlADK* genes, the gene duplication landscape was obtained using MCScanX [66]. Each duplicate segment with *SlADK* genes was selected, and a syntenic map was generated using CIRCOS [67]. The putative duplicated genes were linked by connection lines. To analyze synteny relationships of *SlADK* genes between different species, the genome sequences, genome annotation information, and ADK coding sequences for rice (http://www.ricedata.cn/gene/ (accessed on 9 October 2020)), tomato (https://solgenomics.net/ (accessed on 16 July 2019)), and potato (https://solgenomics.net/ (accessed on 9 October 2020)) were obtained. Fragment lengths in alignment that exceeded 75% of the length were confirmed as tandem duplications. The synteny relationships between the *ADK* family members in tomato and other aforementioned species were determined using MCScanX with the adjusted parameter of minspan (12).

### 4.6. Expression Data Mining of Tomato SlADK Genes

Expression patterns of identified tomato *SlADK* family genes during vegetative and reproductive development were carried out with the TomExpress bioinformatics platform (http://gbf.toulouse.inra.fr/tomexpress (accessed on 12 August 2019)). TomExpress provides a unified and standard method to judge tomato gene expression from released RNA-Seq data sets. Expression data represent normalized counts per base and mean values of multiple cultivars for each tissue and stage, as well as different biotic and abiotic stress treatments. The expression value was appropriately associated with corresponding experimental annotations. Various forms of data output, such as line diagram, heat map, and other graphic types, were utilized to make the web pages more user-friendly. For coexpression and correlation network analysis, the coexpression tool of the TomExpress platform was used to identify genes that displayed similar or opposite expression profiles. The comprehensive visualizations of coexpression results were based on the calculation of the correlation values [68,69].

### 4.7. Hormone and Abiotic Stress Treatments

One-month-old tomato plants that were transferred to a hydroponic box from soil were subjected to hormone and abiotic stress treatments. For hormone treatment, 200 μM ethephon (Eth), 100 μM abscisic acid (ABA), 500 μM salicylic acid (SA), and 50 μM Methyl Jasmonate (MeJA) were prepared. Then, different tomato leaves were sprayed with each of the above solutions. When the solution dropped, spraying was stopped, and the leaves were sealed with transparent plastic film quickly for moisture retention. After treatment for 0, 0.5, 1, 2, 3, 6, 12, and 24 h, samples of leaves were harvested separately. Plant leaves sprayed with ddH2O were used as control. For salt stress treatment, salt (NaCl) was added into the hydroponic medium to ensure that the final salt concentration was 150 mM, it was necessary to submerge root adequately with salt solution. Samples were collected after 0, 3, 6, 9, 12, 24, 48, and 72 h treatment. For cold stress treatment, tomato plants for hydroponic culture were transferred to a cold chamber maintained at 4 ± 1 °C. Leaves were sampled at 0, 3, 6, 9, 12, 24, 48, and 72 h post treatment, and untreated plants were used as controls. For drought treatment, PEG6000 was used for simulating drought. The final concentration of PEG6000 in hydroponic medium was 12%, and it was necessary to submerge the root adequately with PEG6000 solution. Samples were collected after 0, 3, 6, 9, 12, 24, 48, and 72 h treatment. Three individual plants in good status were used for each treatment. After treatment, tissue of leaves in each biological replicate was collected and mixed thoroughly, then frozen in liquid nitrogen immediately and stored at −80 °C.

### 4.8. RNA Isolation and Quantitative Real-Time PCR Analysis

Total RNA was extracted using Trizol reagent (Invitrogen, Carlsbad, CA, USA) according to the manufacturer’s instructions. After RNA integrity detection and RNA concentration measurement, DNase I (Thermo Fisher Scientific, Waltham, MA, USA) was used to remove any genomic DNA. About 2 μg of total RNA from each sample was used for first-strand cDNA synthesis following the manufacturer’s protocol. DNAMAN8 software was used to design primers, and *SlActin* (*Solyc03g078400*) was used as internal control (Table S5). Quantitative real-time PCR was conducted on a CFX96 Touch™ Real-Time PCR Detection System (BIO-RAD, Hercules, CA, USA) using the SuperReal PreMix Plus (SYBR Green) (TIANGEN, Beijing, China). Each reaction mixture was 20 μL sample volume in total, containing 1.2 μL cDNA, 1.2 μL primer mix, 10 μL 2× SYBR Mix Taq, and 7.6 μL sterile distilled water. The PCR amplification cycle was performed as follows: 95 °C for 15 min, 40 cycles at 95 °C for 10 s, and 60 °C for 30 s. Melting curve analysis was performed ranging from 60 to 95 °C to verify amplicon specificity for each primer pair. Relative expression levels of the detected genes were calculated using the standard curve and normalized by the control’s expression. For analysis of the expression pattern of the *ADK* gene family under abiotic stress and hormone treatment, the results were displayed with a heatmap to display gene clustering based on expression patterns.

### 4.9. Virus-Induced Gene Silencing

VIGS (virus-induced gene silencing) was performed using VIGS vectors TRV1 (pYL192) and TRV2 (pYL170) [70]. First, the 380 bp sequence of the *SlADK10* coding region was selected as the interference fragment and amplified using gene-specific primers. The primer sequences were *ADK10*-BamHI-F (5′-ACGCGTGAGCTCGGTAATCTGAGACAGTGAAATCCC-3′) and *ADK10*-XbaI-R (5′-GTAAGGTTACCGAATTCAATCTATGTCTGTCACCTG-3′). Near the 5′ end, ACGCGTGAGCTCGGTA in F and GTAAGGTTACCGAATTC in R primers were homologous arm sequences linked to the recombinant vector. Then, the correct interference fragment after sequencing was cloned into TRV2 vector (digestion with BamHI and XbaI), yielding *pTRV2*-*SlADK10*. Vectors of *pTRV1* and *pTRV2*-*SlADK10* were transformed into Agrobacterium tumefaciens (strain GV3101) and subsequently used for the infection of tomato seedlings [71]. Efficiency of the silencing protocol was examined using a tomato *Sulfur* gene (*Su*) as a marker of silencing in tomato plants. PEG6000 with a concentration of 180 mM was used for simulating drought stress.

## 5. Conclusions

In this study, a total of 11 *ADK* genes were identified and named according to their chromosomal locations. The phylogeny, motif, gene structure, chromosomal location, cis-elements, evolutionary relationship, coexpression analysis, and expression patterns predicted in different tissues were analyzed with bioinformatics methods. qPCR verification results revealed that the expression levels of *SlADK*s in different tomato tissues were basically consistent with prediction results. Additionally, qPCR data revealed that the *SlADK*s responded to multiple abiotic stresses and plant hormones. Analysis of coexpression and correlation networks between *SlADK*s and other tomato functional genes supplies new ideas for exploring gene function. Interestingly, *SlADK10*-silenced plants showed poorer drought resistance than WT plants under drought conditions, indicating that *SlADK10* regulated the drought tolerance of tomato positively. In general, the study provides comprehensive information for the *SlADK* gene family and will aid in determining the specific *SlADK* gene function in further research.

## Figures and Tables

**Table 1:** Features of *SlADK* family genes identified in tomato.

Name	Gene ID	Chr	Genomic Location	Strand	ORF	Exon	AA	MW (kDa)	PI
*SlADK1*	Solyc01g088480.2.1	1	83267069–83271080	–	630	10	209	22872.10	5.76
*SlADK2*	Solyc02g093990.2.1	2	54662319–54663928	–	780	4	259	29003.16	6.45
*SlADK3*	Solyc03g005050.2.1	3	44287–46883	+	852	4	283	31654.06	6.46
*SlADK4*	Solyc03g083610.2.1	3	53563661–53567185	+	714	10	237	26768.76	8.83
*SlADK5*	Solyc03g111200.2.1	3	61838312–61842194	–	735	6	244	26669.91	8.57
*SlADK6*	Solyc04g049690.2.1	4	43054097–43062321	–	810	4	269	30011.55	6.90
*SlADK7*	Solyc05g014980.2.1	5	9188640–9193694	–	729	6	242	26541.56	7.01
*SlADK8*	Solyc06g065270.2.1	6	40705359–40709638	–	861	7	286	31970.68	6.96
*SlADK9*	Solyc08g077300.2.1	8	61201659–61206762	–	651	8	216	24411.10	7.63
*SlADK10*	Solyc09g007180.2.1	9	801969–804985	–	846	4	281	30520.98	6.36
*SlADK11*	Solyc12g010380.2.1	12	3429721–3438938	–	1989	19	662	74110.62	6.67

Chr: chromosome.

**Table 2:** Predicted subcellular location of SlADK family members.

Predicted Subcellular Localization					
Locus Name	Name	Wolf PSort	Predotar	TargetP	CELLO
Solyc01g088480	SlADK1	cyto: 13	none	other	cyto(2.70)
Solyc02g093990	SlADK2	mito: 6	mitochondria	mitochondrial transfer peptide	mito(1.76)/cyto(1.28)
Solyc03g005050	SlADK3	mito: 8	mitochondria	mitochondrial transfer peptide	cyto(1.49)/nucl(1.46)/mito(1.15)
Solyc03g083610	SlADK4	mito: 7.5	mitochondria	mitochondrial transfer peptide	cyto(2.93)
Solyc03g111200	SlADK5	cyto: 8	none	other	cyto(1.83)/mito(1.70)
Solyc04g049690	SlADK6	mito: 10	mitochondria	mitochondrial transfer peptide	mito(1.81)/nucl(1.52)
Solyc05g014980	SlADK7	cyto: 6	none	other	mito(1.83)/cyto(1.51)
Solyc06g065270	SlADK8	chlo: 14	plastid	chloroplast transfer peptide	chlo(2.32)
Solyc08g077300	SlADK9	cyto: 9	none	other	nucl(2.74)
Solyc09g007180	SlADK10	chlo: 11	mitochondria	mitochondrial transfer peptide	chlo(3.70)
Solyc12g010380	SlADK11	chlo: 11	plastid	chloroplast transfer peptide	chlo(3.42)

For subcellular localization prediction, all the SlADK proteins were analyzed with software Wolf Psort, Predotar, Target P, and CELLO. Localization (cyto—cytoplasmic, nucl—nuclear, chlo—chloroplast, mito—mitochondrial) is followed by probability score in each prediction algorithm.

## Data Availability

Data is contained within the article or supplementary material.

## Supplementary Materials

The following are available online at https://www.mdpi.com/article/10.3390/ijms22147708/s1. Table S1: CDS and gene sequence of *SlADK*s. Table S2: ADK amino acid sequences of different plant species for phylogenetic tree construction. Table S3. Features and description of ten motifs of SlADKs. Table S4. Detailed information of cis-regulatory elements in *SlADK* promoters. Table S5. Primers sequences used for quantitative real-time PCR. Table S6. Coexpression and correlation network analysis. Figure S1. Expression level data sets of 11 *SlADK* genes in tomato different tissues and during fruit ripening. Figure S2. Expression level data sets of 11 *SlADK* genes in different tomato tissues under stress treatment. Figure S3. States of *SlADK10*-silenced plant (TRV2-*ADK10*), *Su*-silenced plant (TRV2-*Su*), and wild-type plant (WT) under normal growth conditions.
