# Supplementary material for: Genome-Wide Identification and Expression Analysis of Tomato ADK Gene Family during Development and Stress
Source: Int J Mol Sci. 2021 Jul 19;22(14):7708. doi: 10.3390/ijms22147708 (PMC8305589; doi:10.3390/ijms22147708)
Supplement: Supplementary file 1 [file ijms-22-07708-s001.zip › Table S1 CDS and gene sequence of SlADKs.pdf]

The coding sequence (CDS) of SlADK gene family

>SlADK1

ATGGGGACTGTTGTCGAGTCTGCTAACCAGGGAGCAGTAAGCCTGCCAACCAACAAG
AAGGTCACTGTTATCTTTGTTCTAGGTGGCCCAGGCAGTGGTAAGGGCACCCAATGTG
CTAATATTGTTGAACACTTTGGGTACACCCATCTAAGTGCTGGTGATCTTCTCCGAGCA
GAAATAAAATCTGGTTCCGAGAATGGGACGATGATTTCGAACATGATTAAAGAAGGGA
AGATTGTTCCGTCAGAGGTAACAGTTAAGCTTCTCCAACGAGCAATTCAGGAAAATGG
CAATGACAAATTTCTTATTGATGGTTTCCCCCGGAATGAGGAGAATCGTGCTGCTTTTG
AGTTGGTCACTGGAATTGAGCCTGAGTTTGTGCTCTTCTTTGATTGTCCTGAAGCAGAG
ATGGAGAAACGCCTTTTAGGTCGGAACCAGGGAAGAGAAGATGATAATATTGAAACAA
TAAAGAAGCGATTCAACGTTTACATGGAATCTAGTCTACCTGTTATTGAACATTACAAC
TCCAAGGGGAAGGTTCGAAAGATTGATGCTGTAAAGCCTGTTGGAGAAGTATTTGAAG
CCGTTAAAGCTGTTTTTGCCCCATCTAATGAGAAGGTTGCTGCCTGA

>SlADK2

ATGGCAATGTTGAGCTTCCTCGGAGTTTCCGCCCGAACATTTCTTCGGGCTGCTTCAAG
CAAGTCGGTTCGGGCCTACGGTTCAGCTGTTGCAGCTCACTTTGATTACGACAATGAG
GAGGATATGGAGGAACCATCAGGATCAGTTCCACGCAGAGGAGTACAGTGGCTGATCA
TGGGTCATCCAATGACGCAGAGACACGTGTACGCCCAATGGCTGTCCAAACTTATGGA
CGTTCCATATATTTCCATGGGCTCTCTCGTACCTCAACAACTCAACCCTCACTATAACAA
GATATCAAGTGTTGTGAATGAGGGAAAGCATGTTCCAGAAGAAGTTATTTTTGGTTTGT
TATCAAAGAGGCTAGAAGAAGGTCATTGCAGAGGTGAAAACGGGTTCATTTTAGATGG
AATTCCTCGTACAATGCTTCAGGCTGAAATCCTTGACAAAGTTGTGGACATAGATTTAG
TTCTGAATCTCAAGTGTTCCGTGTCAAAGAATGATAGAAGCAATGGAATTTATTCAACT
GAAGATCAACTCCTTAAGAGAGGTAATTTAATGTCTTCAAGGGTCATGGATGGTGGTGC
CTGGAAAGAGAAGCAATATGATCATGATGAACAGATCAAACCCTTAGAAGAATATTATA
GGAAGCAGAAAAAGCTCCTCAATTATCAAGTAGCAGGAGGGCCTGCTGAAACTTGGC
AAGGCCTTTTGGCTGCATTGCAACTTCAGCACATGATGAGTGCAGTTGGTTCAACACA
GTTGACTGCAGGATGCTGA

>SlADK3

ATGGCATTGCTCAGCCGTATCAGAGCGGCGGCGAAGCCACTTATTCGGACTGAATCTCT
ACGGTCTTACGGATCAGCTGCAGCTCAGCTAGTAGACTACGATTACGATGATTACGAAT
ATGAAGAGTTTCAGAACCGGAGCTGCGTAATGGAGGAATCTGAGGGATCGGTTCCTCG
AAGGGGAGTGCAGTGGGTGATTATGGGTGATCCTATGGCACAGAGACACGTGTATGCC
CAGTGGCTCTCGAAGCTTCTAGGTGTCCCTCATATTTCCATGGGTTCACTCGTTCGCCA
AGAGCTCCACCCTCGTTCTTCTCTCTACAAGCAGATAGCAGATGCTGTCAACCAGGGA
AAACTTGTCCCGGAAGAGGTTATATTTGGTTTATTGTCAAAGAGGCTAGAAGAAGGATA
TTGCAGTGGTGAAAGTGGATTCATCTTGGATGGGATTCCTCGATCAAAGATTCAAGCTG
AGATCCTGGACAAAACTGTGGACATAGATTTAGTTCTGAATCTTAAACGCGCAGAGGA
TTTGGTGTCAAAGAAAGATAAAAGCACTGGGCTTTATCCACCACTGGAATTCCTCCGC
ATGGGGGCTTCTGGAATTAGTACCAGTCGGCAGCCAGAGGGTGGTCATTTTAGGCCTT
CAAGTATCATGGAAGATGTCTCGAGGAAGAACCTGCATGTGCATGCAGAGCAGGTCAA
CCCGCTAGAAGAATACTACAGGAAACAGAGAAAGCTCCTAGATTTTCAAGTGGCTGGA
GGACCTGGAGAAACATGGCAAGGCCTTTTGGCTGCTTTGCATCTTCAGCACAGGAATG
CAGTTGGTTCTACACAGTTGACCGCAGGATGCTGA

>SlADK4
ATGTGGAGGAGGCGTTTCACTTCACTCCCTCTATTCTTTTCGCATCTTCAACAGGTGAG
AAGAGCTGATGAGCTGAAGATTTGTCAAGCATTTTGTACTGAAACTGTCAAACCGCCG
GTAGAAGGTGAAAGTAATTCCGGAAGAAATAGTCCTTTTGTCGCTTTTGTATTGGGAGG
TCCTGGTAGTGGTAAAGGCACTCAATGCCTGAAGATTGCTGAAACTTTTGGGTTCGATC
ACATAGGTGCAGGAGATCTATTGAGGAAAGAGATGCATTCTGACTCCGAGAATGGTGC
CATGATTCAAAAGTTAATGAAGGAAGGAAGTATTGCTCCATCAGAGGTGACTGTCAAA
CTGATTAAAAAGGCAATTGAATCAGCTGAAAATCGTAAGTTTCTAATAGATGGTTTCCC
AAGAAGTGAAGAAAATAGAGTGGCGTATGAGAGGATTATTGGTGCTGAACCGAACTTT
GTGCTTTTCTTTGATTGCCCTGAAGAAGTAATGGTCAAACGAGTATTGAACCGGAACG
AGGGGCGAGTTGATGATAATGAACATACAGTCAAGGAGCGCCTAAAGGTATATAAAGC
TATCACTCTTCCAGTAGCCAACCACTATGCCATGAAAGGAAAGCTTTACAAGGTCGATG
GTACTGGAACTCAGGAAGAGATATTTGAACGAGTCCGTCCAATTTTTGCTTCATTGAGG
TTGTCTACATAA
>SlADK5
ATGTCGACATCATCAGTGAACTTGGAAGATGTTCCCTCCGAGAGCCTCATGTCGGAGC
TTCTCCGCCGCATGAGGTGTTCCTCCAAGCCGGACAAACGTCTTATTCTCATTGGCCCA
CCTGGATCAGGGAAAGGAACACAATCTCCTATCATTAAAGATGAGTATTGCTTGTGTCA
TTTAGCCACGGGGGATATGCTGAGAGCTGCTGTTGCTGCCAAAACACCTTTGGGCATTA
AGGCGAAGGAGGCCATGGATAAGGGAGAACTTGTTTCTGATGACTTGGTTGTTGGGAT
CATAGACGAAGCATTAAAGAAGCCATCATGTCAAAAAGGATTCATTCTTGACGGGTTTC
CTAGAACTGTGGTGCAAGCAGAAAAGCTTGATGTGATGCTTCAAAATCGAGGTACTAA
AGTTGATAAGGTTCTTAATTTTGCGATTGATGATGCCATCTTGGAAGAGAGAATTACCG
GACGCTGGATCCACCCAGCTAGTGGTCGGTCATACCATACAAAATTTGCACCTCCTAAA
GTACCTGGGATAGATGATGTCACTGGAGAGCCATTGATACAACGAAAAGATGATACTGC
TGCTGTTCTCAAGTCAAGGCTAGAAGCCTTCCACAGGCAAACTGAGCCGGTCATTGAC
TACTATGCCAAGAAGGGTAATGTAGTGAATCTTCCTGCTGAGAAACCACCACAGGCAG
TCACAGCTGAAGTCAAGAAAGTTCTCTCCTAA
>SlADK6
ATGGTGGTTTGGACCCGTGCAGTGGTAAGAACATGGAGATGTAGACCCACCAATTTTA
GCCGGGCTTTTTCTGAAAAATTACCTACATCGGAGCCTAAGGGCAGAAATATTCAGTGG
GTATTCCTGGGTTGCCCTGGTGTCGGAAAAGGGACTTATGCAGCTCGACTTTCTAAGCT
TCTTGGCGTACCCCACATAGCTACTGGTGATCTCGTCCGTCAACAATTATCCTCTCATGG
TCCTCTTGCCTCAAAGCTTGTGGATATTGTTAGCCAAGGGCAATTAATTTCAGACGAAA
TTGTAATAGATTTGCTATCTAAGCGTCTTGAAGCTGGAGAAGCAAAGGGTGAAACAGG
ATTTATTCTTGATGGATTTCCTCGAACTATACGGCAGGCGGAAATCTTAGAGGGAGTGA
CAGACATTGACTTGGTGATTAATCTGAAGCTTCGGGAAGATGCGTTGATTGCCAAGTGC
TTAGGAAGAAGGACTTGTAGTGAGTGTGGAGGCAATTATAATGTTGCGTGCATTGATAT
GAAGGGTGATGATGGAGAAACTAGAATGTACATGCCTCCCCTTCTCCCTCCTCCGCATT
GTGAAACCAAACTTATTACACGGTCTGATGACACTGAAAATGTTGTGAAGGAACGCCT
CCGCATCTACCATGAAATGAGCAAACCAGTAGAGGACTTTTACCGCCAGCGAGGTAAG
CTGTTGGAGTTTGATCTCCCTGGAGGAATTCCAGAGTCATGGTCAAAGTTACTTCAGGC
TTTGAATATCTACGACGACGAGGATAAGAAATCTGCTGCAGCATGA

>SlADK7

ATGGCTGCTTCATTAGAAGATGTTCCTTCAGAAAGCCTAATGTCTGAAGTTCTCCGCCG
TCTCAGATGTTCTTCTAAGCCTGACAAACGTCTCATTCTCATAGGTCCACCGGGATCTG
GAAAAGGTACACAATCTCCTATCATAAAGGATGAATACTGCTTGTGCCATTTGGCCACC
GGTGATATGCTCAGAGCTGCTGTTGCTGCTAAAACTCCACTTGGGATTAAGGCAAAGG
AAGCTATGAACAATGGTGAACTTGTGTCGGATGACTTAGTTGTTGGTATAATTGATGAA
GCAATGAAGAAACCTTCATGTCAAAAAGGCTTCATTCTTGATGGTTTCCCAAGGACAG
TGGTTCAAGCAGAAAAGCTAGATGAGATGCTTCAGAAGCAGGGGTCCAAGATTGATAA
GGTGCTCAATTTTGCAATTGATGATGCAATCTTGGAAGAGCGGATCACAGGCCGGTGG
ATTCACCCTTCAAGTGGTAGATCTTATCACACCAAATTCCAACCCCCAAAAGTTCCTGG
CGTCGATGATGTCACTGGAGAGCCTTTAATTCAACGGAAAGATGATACTGCTGAGGTTC
TTAAATCAAGGCTAGATGCATTTCACCGTCAAACTGAGCCGGTAATCAATTATTATTCCA
CCAAAGGTGTTGTTGCAAGTCTTCATGCTGAGAAACCACCAAAGGAAGTTACTTCTGA
GGTTAAACATGTGTTGTCTTCTTGA

>SlADK8

ATGGCTTCGTGCTGTTCATTGAGCTTCTCAACAGTCTCTTCAAAGCCTAACAAGCCTTA
CTCATCACCAATTTCTTCTTCTCTTGAGCTCCCCTTTACTTCCCAGTTGCCATTTTCTAA
AAAATATTCACTTTATTCCAATCATACCCTTCTCCAAACTCAATGCCGGAAAACCCAATC
ACCGGATTGTCCCAGTTTCTTGGTTGTGGGATCTGCGAAAAAGCAAGAACCTTTGAGG
GTAATGATATCGGGAGCTCCTGCTTCTGGTAAAGGAACGCAATGCGAGCTCATTACCAA
GAAGTACGATTTGGTGCATATTGCTGCTGGAGATTTATTGAGGGCTGAAATTGCTGCAG
GCACTGAAAATGGGAGGAGAGCAAAGGAGTATATGGATAAAGGACAATTGGTACCAA
ACGAGATAGTTGTAACGATGGTCAAAGAGCGGTTGATGTGTCCAGACTCTCAAGAAAA
GGGTTGGCTTTTAGATGGATATCCTCGGAGCTTGTCTCAAGCAGTAGCTCTCAAAGAGT
TCCAGCCAAACCTTTTCATTCTTCTGGAAGTACCTGAAGAGATACTTGTTGAGAGAGT
GGTTGGCCGTAGACTAGATCCTGTAACTGGGAGAATATACCATTTGAAGTATTCTCCGC
CAGAGACCGATGAAATCGCTGCAAGGCTTACCCAGCGCTTTGATGATACAGAAGAAAA
GGTGAAGCTGCGTCTGCACACTCACCGTCAAAATGTGGAATCAGTTCTCTCAATGTAC
AAAGATACTATATTCCAGGTGGACGGGAGCGTTTCCAAAGAGGAAGTATTTGCTCAAA
TTGATGGTGCATTAACTCAACTTCTTGAAGCAAAGGAGTGA

>SlADK9

ATGGATTTACACAAGGAAGGTGATACAGGCTCCGCAAAGCAGAAGAAGGTCAAGATT
GTTTTTGTTATAGGTGGTCCGGGGAGTGGTAAAGGAACACAATGCAAAAGAATAGCAC
AACAATTTGGATACACTCATCTTAGCGTTGGCGAGATTCTACGTCAAGAAACCAGTTCT
GGTTCTGAAACTGGCCATATGGTTCAGAAAATTATGAAGGAGGGAAAGCTTGTTCCGT
CGGATGTAACAGTGAGGCTTCTTCAACAAGCCATGCAGGGAATTGATAATGACAAATT
CCTCATCGACGGCTTCCCCCGGGATGAAGAGAATGTTAAAGCATTTGAGGATCTTACA
AAAATGGAGCCTGAGTTTGTCCTTTATTTAGATTGTCCACAAGACGAAATGGAGAAGC
GCTTGCTATCAAGAAATGAGGGAAGAGAGGATGATAACATCGAGACAATAAGGAAGC
GATTGAAAGTTTTCGTGGAGTCAACTCTCCCTACAATTGAATACTATGAATCAAAGGGG
AAAATTAGGAAGGTTGATGCTGGAAAATCTATTGATGAGGTTTTTGAATCCATCAAAGT
TATTTTCTCACCAGGAAAAGATAACAAGATGCCACCAAGTAAACACAAGTGCAAATGC
TTGATACTTTGA

>SlADK10

ATGGCGGCCATGATCCGCCTGTTCAGATCTTCATCATCTTCATCATCTCTTATCAGTAGAT
CTTTATCTACAGCAGCTGCATCTGAGACAGTGAAATCCCGATCTTACCCTCATTCTACA
AGTGTTGAACCCAAGGCTAAATCTGTTCAATGGGTCTTTTTGGGTTGTCCTGGTGTCGG
AAAAGGTACATATGCTAGCCGTCTCTCAACCCTTTTAGGCGTTCCTCATATTGCTACTGG
AGATCTTGTTCGTGATGAGTTGAAATCTTCAGGTCCTTTATCGAAACAACTTGCAGAGA
TTGTCAACCAAGGAAAATTGGTTTCAGATGAGATTATACTGAATCTACTATCCAAAAGG
CTTGAGAGTGGGGAAGCTAAGGGTGAAGCTGGATTCATACTTGATGGTTTCCCTAGAA
CTGTGAGACAAGCAGAAATATTGACCGAGGTGACAGACATAGATTTGGTGGTTAATCT
CAAGCTTCCAGAGCGGGTATTGGTTGAGAAATGCCTTGGCCGAAGAATCTGCAGTGAA
TGTGGAAAGAATTTCAACGTGGCATCTATAGATGTCGCTGGTGAAAATGGGGCTCCTAG
AATCAGCATGGCTCCCCTTAATCCTCCCTCTCAGTGTATATCAAAGTTAATCACTCGAGC
AGATGATACAGAAGCCATTGTGAAGGAAAGGCTCAGTATATACTGGGATAAGAGTCAG
CCTGTTGAGGACTTCTACCGTAGCCAAGGTAAGTTACTGGAATTTGATTTACCGGGAGG
CATCCCCGAATCATGGCCTAAGTTGCTGGAAGTTCTCAACCTTGATGAACAAGAACATA
AACTGTCTGCTGCAGCTTAG

>SlADK11

ATGGCGATGATAGCTTCAGTCACTATGAATTTTCCTCACATTTCTACTCATAATATTTCTT
CAAATCAAACGTTTTCTCCAATCTGTACTAATAATCCTTCTAATTTCTCATCATCGTCGTC
TTCATCAATACCAATTTCTTCGAATTCTATCCGTTTATCATCTTCAATTGCTTACTCGGAA
CAACTAATCGCGTCTCACAATGTCAATCGACGTACTAAGAATCGCAAAATCAAGGTGAT
ATCTGCAAGAAGTGAGCCTTTAAAGGTGATGATATCAGGTGCCCCTGCATCTGAAAAG
GATGTAGTGGGATGGATGAAATCCCTCTACCCTTTACAAGAGGTATCGGGTTTGAGCAA
CGGGAGTGGAGAAATCTCTGGTAGTGAGCGCTTCCTCCTTCAATATTGGAGTTACGCA
ACACAAATTTTGTTTAGTCAGACCAATGGGTTGATTGAACAACAAAAGTTTGTCTTTTG
TGGTTTTGGATTGGTGCACATATCAACTGGTGATCTCCTACGAGCTGAATTATCGGCTG
GTACAGATATTGGAAATAAAGCTAAGGAGTATATGAACGCTGGTCGCCTGGTTCCTGAT
GAGATTGTAACAGCTATGGTGACAACACGATTATCAAAGGAAGATGCAAAAGAAAAA
GGGTGGCTTCTGGATGGGTATCCACGAACGTTGGCTCAAGCAGAAAGTCTGGAAAGG
TTGAATATCAGACCAGATATCTACATTGTGCTTGATGTTCCTGATGCGATTCTTATTGAC
AGATGTGTTGGTAGAAGGCTAGATCCTCTCACTGGTAAAATATACCATGTTACTAATTTC
CCTCCAGAGACCGAGGACATCAAAGCAAGGCTCATAACTCGTCCTGATGACACAGAG
GAAAAGGTGAAATCACGTCTGCAAATATACAAGCAAAATGCTGAAGCAATATTACCAG
TGTACTCAGATATAATGAATAAGATTGATGGGAACCGCGGCAAAGATTCAGTATTTGCA
GAAATTGATTCCTTATTGTCACGTGTGCAGAAAGAGGAACAAGATGCAAGAAAATCAG
AAGAATCAGCAATTTCCAGTACTCGAGCTGATATGGCATCTTTGAGCAAGGATTGGAG
AGGAATACCTACTAGACTGAATAATATTCCTCACTCAAGAGAAATTAGGGAATATTTCTA
CACTGATGTGCTTCAGGCTACTCAAAGAGCTGTCAATGATGGGAAAACTCGTTTAAAG
ATAGAAATCAATATCCCGGAGCTGAATCCTTCAATGGATGTTTATCGAATAGGTACTCTA
ATGGAACTTATCCGTGTACTTGCTCTTTCATTTGCTGACGATGGAAAGCGTGTCAAGGT
TTGTGTCCAAGGGTCTATGGGGGAAGGTGCACTTGCAGGGATGCCTTTGCAGCTTGCA
GGAAGTCGAAAGATATTAGAGTACATGGATTGGGGTGATTATGGCGCGTTGGGAAACT
TTATCAATATTGGTTCTATAGGTGGCAAGGAGGTTGAAAAACAAGATGACGTGTTCATT
CTAGTGGCTCCCCAGAATGCTGTCGGAAATTGCATCATAGATGATATGAGAGCTATGAC

TGATGCAGCCGGTAACCGACCGATTATTCTAGTCAACCCCAAACTAAAGGATTTACCTG
CTTCAAGTGGTATCATGCAAACAATGGGTAGAGATAAAAGATTGGAATATGCTGCATTG
TTTGAGATATGCTATCAATTCCGGCTACTCTACTATGCAGGAACACAATATCCTATTATGG
GGGCACTCAGGATGTCGTACCCGTATCCTTATGAGTTATACAAGAGAGTCGATGAATCA
CCGGGGAAGGAGAAATACATATCCTTGGCAACATTTGCAAAGAGGCCAAGTATTGATG
AAATGAACGATGCCTTCGAAGGAAAATCAAGAAATCAAGAGAAAAAAGCAGAGGGAT
TTTGGTATGTGATTAACTATCCTCCGTTTTATCTATGTTTTGAGTAG

The gene sequence of SlADK gene family

>SlADK1

TTGTATTTTCTGAAGCTTATTAATGTGAACATGTTCATTTTTCTTATTTGATCTGAAGCTG
GAATATATTAAGTGAAGATTCCAGCATCTGATCTCTGAAAGTGCAAGTCTTTTCCTTTTA
GATGTTCAGTCCCCCCTCCTTTATTTTTAGTGTCAACATAATCCTGAGTAATTGATTGAG
TCTGTCTTTCTGCAGACAATTTTGTAAGACCAAGTTACCAGAAATCCATGGGGACTGTT
GTCGAGTCTGCTAACCAGGTAGCTTTCTTTACAACTAAATGTGCTTCATATATCTAAAGA
ATATTCTTATTTACCTTGATTATTGGTGTCCACTCCTTTTGTGAATGAGTTCTAATTAATAT
ATCGTCTAAGCATCTGGATTCTAAACAAAAGTGAAATGCTTTTTACCCTGGATGACATTA
TTGGAGGGATGCTTGACCTTTAAGCTATCACTAACTTCTCTATTGTCAAATGCAAATAAG
AACTTCCACTATAATTAAAGAAACCTGCAGAAATTTTTTTGAGATTTTAAAGACTAAAA
ATAGTGGAGTTGTGGTGTGTCTTACAACATATGGGGCTACAACAATTGCACTGTGAAAT
TAGATACCGATTTTATAGATTTGCAGGATGCATGTACCAACAGCAACACCAAGAAAGAA
CGGAAATAGGGGTCTAACTCCCATTGTCCTCCCTGAAGTTCTTTGTTTATAAAAGGGGT
CTAACTCCCATTCTCCTCCCTGAAGTTCTTTATTTATAAAAGTTCTCATATAGTTCTATGA
GTTCTTCTTGCTAGGCATTATTCTATTATCATAAGAACATTATTGTTGAATATAAAAATTC
CTCCCTATACATAAATTACCACCACCACTTTTAATAAATAGTATAATTATCCATGATTTATG
CTCCTGCACAGTGTCATGCAGATGCCACATTCTCCTTAATTTGATATCTTCTTAATAATG
AATTGCAGGGAGCAGTAAGCCTGCCAACCAACAAGAAGGTCACTGTTATCTTTGTTCT
AGGTGAGTTTTTCAACACCAACATTGCATGATCTTGTGGCATTCTTGATACGGCACCTC
ATAGTTTGGCAAAGTATTTTGTCCCTGTATTTAATTGTGAGGATTAAAGGTGGAACTTGT
TTGAATCATATGTTTGCCATCATATGTTATCTGATCTACATGTTCAGTTAGTGCTGCCACT
AATACCGATTAAATGCAGCTCTTGACAGTCTATATGTCTAACTTTTTAGGGTTCGACGCC
GTAACTATAGCTTCTTTGGTTGAAACTGCCCCCCATTTATTGTATTAATAAACTATTGCC
TGTATGACTGCAAGAATCCTTTGGGCATTTACATCCAATTTTAATAAAAATGAAACAAG
AAGCTATATTGTGTAAAAGCCAGATCAGATTGGACTGGTTGGAAAATTTTTTACTCCAG
TTTAGATTGTGATGGTATAATTGATTTTCTTAAGTAACGGGGCTTCATCTTTGTGATTATT
TACATACAAGTTAACAGGAGGGTTTTGTAGTAATTAACTTGTGAGGGTTACTTGAGAAA
ATTTGAAGGAGGTATATCTATTTCACCATAAACTTAATATACTCTTAAGCTAATTGCTCTA
CTTCTTTATCTAACTCACTCAAAAAAGATTTTTTGTGTTTGGCGTTGTGAATAAAACAAT
TATTTGGAAATATCCTATTATGGGGGTGCAACCTGTGCGGATTTCTGGATCAAATACACA
TCCTCATTGCACATACATATTTGTTGATGCAATCTGTACCATTGATGTTAGATTTTTATCC
TGTGCTAAGATTTGTCTCCTCTATTAACTTGGACAGGTGGCCCAGGCAGTGGTAAGGGC
ACCCAATGTGCTAATATTGTTGAACACTTTGGGTACACCCATCTAAGTGCTGGTGATCTT
CTCCGAGCAGAAATAAAATCTGGTTCCGAGAATGGGTAGGATATCTTGTTTTGTTTGTG
TGTTGTTCGATCTTTCTCCTTACTTTTTACCCTTTGTCATTTCAACCAACATTCTGTGTAG

TAGATCAACAGCTGTTGCTAAGTTGCATCATCTCTTTGAGTTTTGTTCAATAGCTTCAAG
CAGTATTTATTGCACACAAAGGATTAATCCTCAGTACTGTGCTTTTGAATGTACAGGAC
GATGATTTCGAACATGATTAAAGAAGGGAAGATTGTTCCGTCAGAGGTAACAGTTAAG
CTTCTCCAACGAGCAATTCAGGAAAATGGCAATGACAAATTTCTTATTGATGGTTTCCC
CCGGAATGAGGAGAATCGTGCTGCTTTTGAGTTGGTCGTGAGTTCCTTATTCCACAGCT
CTTTTTAGCGGCATATTGATAATTCTCCTATTTATTAAGAGTTGTTAAAATTTAACTATCAT
ATTTTAATGTCTCCCAGACTGGAATTGAGCCTGAGTTTGTGCTCTTCTTTGATTGTCCTG
AAGCAGAGATGGAGAAACGCCTTTTAGGTCGGAACCAGGTTAGACATATTATAAGACT
TCTTCTCATGCTTACAGTTCTTTAAACCTTTATGGTATTCTTGTTTCTTTTAAGTTAATCT
TCTTTTCTTATTATTGTGATTGATTTTCTTCTCATTAACTTTGAAATTTTTGTTCTTTAGGG
AAGAGAAGATGATAATATTGAAACAATAAAGAAGCGATTCAACGTTTACATGGAATCTA
GTCTACCTGTTATTGAACATTACAACTCCAAGGGGAAGGTTCGAAAGGTACTGCTTTAT
CTTTCAGCCTCTTTTTCTTCCTCCCCTCCATTCCAGAGGGAGAGACTCTGCATATTAAAC
AGAAATTGACATCTTTTTTGCCTTCAGATTGATGCTGTAAAGCCTGTTGGAGAAGTATT
TGAAGCCGTTAAAGCTGTTTTTGCCCCATCTAATGAGAAGGTATAGTAGAAATGATGGT
TTAGTGAATGTTTAAACGATATTAAGACCTAGTAACTTGCATGGAAAAGGATATCTGAC
GTATGTAATGTAATTGGGAGGTAAATGTCCTCTGTTGCAAATGTTAGGGCTTATGCATCG
CTAGATTTATTCTACATTATGAGATCAGTAAATTAATACTTAGTGTTGAGAATCCTGTGAA
TGTTTGCCTTACAATTCAAACAATTTCTGCATATATTTTGAGTTCATGGAGCATTATTTTT
ATTTATCATTATTGATATGGTTAGATTTGCTCCTAAAATTTGCCTTATCCAGGAGAATTGT
CTCCATAATAATGGAGTATATGGGATGATTCACTGTGGTTGGCTGCTAATTGAGCTCATA
ATATGAGCTGATCTTCAGTTTATTTGTATGAAAAACAATTAGGCCTCAATCCATAACAAG
TTGGGGTCAACTATATGAATCCTCATTAACCATATTAATCCAATTAAAATTCATCTCAGAC
CAGCATTTATACAAAATATAAAACTTAAAGTACTTGAAGTTCTCTAACTTTGTGCAGTCA
TAAAAATCTCTTCTATTACCAATTGATTGGTATGGGCATATATTCTGTCGTCTTTATTTGTT
TGTCAAAATAACAATATTTTCGTTCTGCATATCCTTTATTTCCTTTTTCTTGTTATTTTATA
CATATTGTTCTTGTATGTAACAGGTTGCTGCCTGAGAATTCATTAGTAGAGAGGCATCAC
AATTAATTGTTATGGTAATTAAAAGAACCCTATACTTGGGCTTGCGTAAATGCGTGCATC
TGTTTCTGCTGCTTTGAATTGATTTTACTGTTAACATTTTCTTTTGTCATTTCTTTCTCTC
CAGGTTGTTTGAATATTGGTGCTTGCTGTGTGTCTTCAGCAGCAAAACGATGCATCTTT
ATAGATTCTACTTTTTGATACGAGTTTATGCAAAATTTTCTTTGTTTTTCCTCTAATTTTTT
TGTTTCTTTTGAAGGAAACACAATGTTGTATTATTAGTTTATCGTCTCTGTATTGTTATCG
TTGTCAATTCTGATATCTGATAAATAAGATTATAAGAGTTTCTTCGTCT

>SlADK2

ACGCCATGGCAATGTTGAGCTTCCTCGGAGTTTCCGCCCGAACATTTCTTCGGGCTGCT
TCAAGCAAGTCGGTTCGGGCCTACGGTTCAGCTGTTGCAGCTCACTTTGATTACGACA
ATGAGGAGGATATGGAGGAACCATCAGGATCAGTTCCACGCAGAGGAGTACAGTGGCT
GATCATGGGTCATCCAATGACGCAGAGACACGTGTACGCCCAATGGCTGTCCAAACTT
ATGGACGTTCCATATATTTCCATGGGCTCTCTCGTACCTCAACAACTCAACCCTCACTAT
AACAAGGTATTTATTTATCTTTTAACAATATGACCAATATGCTTTTATTGAAATAATTTATG
GTTGTATGAAGATATCAAGTGTTGTGAATGAGGGAAAGCATGTTCCAGAAGAAGTTATT
TTTGGTTTGTTATCAAAGAGGCTAGAAGAAGGTCATTGCAGAGGTGAAAACGGGTTCA
TTTTAGATGGAATTCCTCGTACAATGCTTCAGGCTGTATGTATACCTCTCCCTCCTCACTT
AAGGAAGGGCTCTTCATTTTTCTCATGAAAAATTGAGTCGGTTTTTCTGATATATCTTTA

TGTAGGAAATCCTTGACAAAGTTGTGGACATAGATTTAGTTCTGAATCTCAAGTGTTCC
GTGTCAAAGAATGATAGAAGCAATGGAATTTATTCAACTGAAGATCAACTCCTTAAGA
GAGGTAATTTAATGTCTTCAAGGGTCATGGATGGTGGTGCCTGGAAAGAGAAGCAATA
TGATCATGATGAACAGGTATCTGTCTGACATATTGTAAATTTTACAAGTTAACAATAACA
GTAGCCAAGAAAACATTTGAGATTGCCTAGCAGTTCTGTTACTGTAAATATATGAACAA
AAGATTTGTCACAAAAATTAAAAGCGTTCATGATCATCCATAGTTGGACTAATCATGCT
GAAGAGTTTATATCACAATGTCAAAAGATATATCTCAAACCACTCAATTCAATATGCTTT
CACATTTACTCTCTGCATGTGCTTCACAGATGATGAGCTTTATATATCTTAGTATGATTGT
CACCATCTTGATGCAGAATCATTACTCATTGTTTTCTCAGTTGAACTACAAAGGCAACT
AATACATGTTATTGTTCTGTATATTATTTTGAAGATCAAACCCTTAGAAGAATATTATAGG
AAGCAGAAAAAGCTCCTCAATTATCAAGTAGCAGGAGGGCCTGCTGAAACTTGGCAA
GGCCTTTTGGCTGCATTGCAACTTCAGCACATGATGAGTGCAGTTGGTTCAACACAGT
TGACTGCAGGATGCTGATACGATTCTCCTGTGTTACTTAATTCTTTTTGCAAGTATATTAT
GTGCAGTGATATGAGTGTATCAATATCAGTTTTGACCGGCAATGTATAGCTAGTTTTTGT
TTCATTCTTGTTTACGAGTGTAACCATGTTATCTCTAGCAGAACACAGTTTGTAAAATGT
TCCATCTATATAACTATTGAGTGTGGTTTCGTGTGTTAACTTTGTAGTTTGAGTTTATACT
GGTGAAGTAACTATGGAAACTACTATACATTATGAAGCAGTAGAACAACTGCAATAACT
CAATCAC

>SlADK3

TCACTCTCCACCATCTTCGCGTCTCTAAAACCCCAATTTGCTTTCCTTAGAGAAACCCT
CTTGTTTTCTCTGGGCGGCTATACAGAGAATCATTTTCCAGCCGGCGATGGCATTGCTC
AGCCGTATCAGAGCGGCGGCGAAGCCACTTATTCGGACTGAATCTCTACGGTCTTACG
GATCAGCTGCAGCTCAGCTAGTAGACTACGATTACGATGATTACGAATATGAAGAGTTT
CAGAACCGGAGCTGCGTAATGGAGGAATCTGAGGGATCGGTTCCTCGAAGGGGAGTG
CAGTGGGTGATTATGGGTGATCCTATGGCACAGAGACACGTGTATGCCCAGTGGCTCTC
GAAGCTTCTAGGTGTCCCTCATATTTCCATGGGTTCACTCGTTCGCCAAGAGCTCCACC
CTCGTTCTTCTCTCTACAAGCAGGTTTTTGTTTATTCTGCATTTCTGTTTGTTTGAGCTTT
CAATCTATCTGCAATGGAAATATGAAAATAAAGATTGTTCTTTTAGTTATTAGCTGGTCAT
ATTTTTGACAAATTGTTAAAATAATTGAAAGAATTAACTTTGAATTGAGGGATTGAATTA
CAAAATTTACATATAATTGATCTATGCTAGCTCAGAATGAAAAGTTTATAAGCTTGATTG
AGCTCAAACCTTTTAATTGTTTGCTTGATTATATGATTGATCTCTGCATTAACATATAATTT
ACATAAGTAGAAAAGTTGCACTAAATTTGATTTCCCTTGAGAAATGTCATGTAAAATGG
AACAAAGGAGTACTTTCTCTTTTGCTTTAAGATAGTCTCACATTACACCTGTTCTTTTTG
TTTGTTTAACCCAATAATACTTTCCATCTTTGTTTATGGCTCAATGAAGATAGCAGATGC
TGTCAACCAGGGAAAACTTGTCCCGGAAGAGGTTATATTTGGTTTATTGTCAAAGAGG
CTAGAAGAAGGATATTGCAGTGGTGAAAGTGGATTCATCTTGGATGGGATTCCTCGATC
AAAGATTCAAGCTGTAAGTATAGCCTCCCTTCCGTATCGTTAACATGTCTCTCTACTTCT
GAGGTAGGGGTGAGGTTTGTGTGCCCTCCACAAACTCCATGGGTGAGGTTTGTGTACC
CTCCACAAACTCCACTTTGTGGGATCACCGTGCGCATGTTGTTGTTGTTGTTGTTGTTG
ACTTCTAGTGTTCTGGACAGAATAATACATAAACTTGTTAATAAAACGTCGAAAGTAAA
TTTTGTTATACTTTGATCAAAGAGGCCTGTTTATTTTCAACTGATTTATGTTGAACTGGT
TTGTTTTTGCAGGAGATCCTGGACAAAACTGTGGACATAGATTTAGTTCTGAATCTTAA
ACGCGCAGAGGATTTGGTGTCAAAGAAAGATAAAAGCACTGGGCTTTATCCACCACTG
GAATTCCTCCGCATGGGGGCTTCTGGAATTAGTACCAGTCGGCAGCCAGAGGGTGGTC

ATTTTAGGCCTTCAAGTATCATGGAAGATGTCTCGAGGAAGAACCTGCATGTGCATGCA
GAGCAGGTACCTAATGATGCTTTAAATTTTACAGTTGCAGATACTCTAAAAAAATTGATA
TTGTAACTTGAGATGAATGAAATTGTAGATTGCCTCTTTTAACATTCAATGAATTGGACT
ATAGATTCCCTCCTTAGCATTCTTGATGTGTGTAAATACTAGTGCTTGCTGAATTCAACA
TGCAAATGTGCTGTTTCAAAATGTCATTTTAGGCCTTCAAGTATCTTGTTTGAGGAAAA
ATCAAATCAAAGATCTTTTGTTTGATAGCTAATATACGGAGTACCTGATTGCTAGTTGAC
GTAACCACACTGAATTGTTTATATATCTATGCCATTTGTTGTGGAACTGTTGGTACTCAC
TTGGTCTTCTCACGTAGTTTTACTTTGTTTGTGCAAATATCTATATGTTTCAAAAGTGATG
TTTCCTGCAGTTGTGATGTGATGTATGTGAATTGTATATTCGATTTGTTTAAATATTCTGT
TTGATTTGTCTTTCATTTAATAGCATGATAAAACTAATTGCATGATCTTGGATCAAAATTT
TCTCAGTAAAAACAACATCAACTGATACATATCACCATTCTGTCTTATATTAGGTCAACC
CGCTAGAAGAATACTACAGGAAACAGAGAAAGCTCCTAGATTTTCAAGTGGCTGGAG
GACCTGGAGAAACATGGCAAGGCCTTTTGGCTGCTTTGCATCTTCAGCACAGGAATGC
AGTTGGTTCTACACAGTTGACCGCAGGATGCTGATCTTCATTCTTTTTGCAAGCTTACT
AAGTATAGGAAATATGAGTGTAACAAACTCTAATTTTTGGGCGGGAATACATGTATTTTT
GTTTCATTTTTGTTTAAAAGAGTACAATCTAGTTCTTCTGCAGAAGAGGTGTGAGCATT
AGACAAGCTTACATTATACTCTCCATATATAGATTTCTACAAAGTGCATTTTTCATGAATG
CTTATGTTGTGTAAAACTTCAAATCCACATTTATTAGTTCGTGTTGTGGCAGTTGTCACC
CATATGATTCTTGAATGTATCGTGTTCTGCAAAGTTTTGGC

>SlADK4

GAGATGGAAGCTCTCGAAATAGAGTTAATTGGCCTTGGGCCTAGCCTAAACTCAAATAT
CTAATTGAAGAAGGGGCAAGCCGATCTAGAAGGAACTAATCGGCCCTGAAAGTGTTGC
AGCGTTTATGCGAGTGAACGTGCAGACAGGTATTGTATTGTTTATGGGACTCACGCTTT
TACACTCACTCAACGTGCTTAATACAAACGCTCTTTCGCCATTCAAACCCAAATCCAGA
AAACGAAACACCGATCGAAGATGTGGAGGAGGCGTTTCACTTCACTCCCTCTATTCTT
TTCGCATCTTCAACAGGTAATTTTACTTCACAAATTGATCAAATCTCTGTAGTTTTGCTG
CTGCAAATCTCTCAATATTGTTCTCAATTTCTCCAGGTGAGAAGAGCTGATGAGCTGAA
GATTTGTCAAGCATTTTGTACTGAAACTGTCAAACCGCCGGTTATTCATTTTATCTCTCT
CTTAATGTCTTGTATTTGTTTTTAATCATCTCTGAAATGTTTTTTTTTTCTACAAAATGTTA
TTGACTACTGAATTTTTTCCCTCGAATGCATGTAGGTAGAAGGTGAAAGTAATTCCGGA
AGAAATAGTCCTTTTGTCGCTTTTGTATTGGGTAATTAATTTCGCATTTTAGTCTAATAAT
AACTGTGCGCTATTCGGACACTAAAAGCTAAATCTCCTTTTATCTCTTTGAAAATTTGAT
TAATTTATCTCTCATGTGATCTCAGTTAAATCTATCCTGATTGTAGCTCATCTGAGATAGA
TATCACTCCAACCTCTTAAGTAGATAGATGTGCTACTCTTTTGTAGTTGTTTGTTAAAATA
TTTTATTTTTCTCCTGTTTTTCGGGGGGTTTTGGTCCGGGACAGGGTGAGAGGGGATGG
AACAATACAAGTTTTTTTTTTTTTTTTTTGAGAATTTTGACACTGATTTTCATTTTTGATA
ACCCTGGTGTTCGGGCCATTTTTCTGCACCTCAACTAATTCCATGTAATTTGCTTTTGTG
CACCTCTACTAATTCCATGGAAATTCCTGCCACCTCCTAGCAGCAAAAGGGACCAGCTA
ACTCTGTCTACCACGGCTAGGACAGTTGACTAAAAATCAACTAGTGTTTTTTGTCTTTG
CGGAATTTGAATTTGAGACCTCATAATTCTCAACTAATTCTTATTTAATTATGTATAACAT
TAAGCAAAAGATAATGAGGTACAAAGGATATCCTTATGGCGAGATGGACTTTTTCATGA
TACTGATTAGGTCACTCTTGTTGGAAGGAAGAGAATATGTCTAGAAGTAACATGTCATA
TTGCGTATTATGTCTGTTGTTGAGCTACCTATGCTCATAGCACTTTTTTTGTTTAAACCAG
TTTTTAATCCTTAACGTTGCGACATATTGTCCATCTTACACATGCTTGTGTGATCAGAAA

TTTGTCAAGTTTGCTGAAGAGTCTGCCTTCATCTCATTCTTTGCTAGTTTGTAGTGGAA
AGTGAATAAACATAGGTTCCTTTGCAGACAACCTACAATCAGTTTGTTTAATAACTATG
AGGGGTTTAGTTGATTCTTTCCTTCTTAATGACAGTTTTCAGCTAAAATCACATGATTAT
ACATGAGAATAGACAAAATGACTATTTTACTAGATTATTTGGGTAAAATATAGAATTACT
GAGCAGTATTTGCTGTCCTTTTAGGAGGTCCTGGTAGTGGTAAAGGCACTCAATGCCTG
AAGATTGCTGAAACTTTTGGGTTCGATCACATAGGTGCAGGAGATCTATTGAGGAAAG
AGATGCATTCTGACTCCGAGAATGGGTATGAACTCCATTTCTCATTGTCGATTTTTAGTT
CATTTCCAGAAAAAAATTTGTAATGTCATTCTATCTAAATTCAGTGCCATGATTCAAAAGT
TAATGAAGGAAGGAAGTATTGCTCCATCAGAGGTGACTGTCAAACTGATTAAAAAGGC
AATTGAATCAGCTGAAAATCGTAAGTTTCTAATAGATGGTTTCCCAAGAAGTGAAGAA
AATAGAGTGGCGTATGAGAGGATTGTAAGTTTCTTTAATTAGATTCTTATCTTTATTTATT
ATAATGAGCATTATCTATTAAGTTATACTCTAGGAATGTTTAGTTGTGTAAAAGGAACTT
TTGCTAGTTTAGCCACTGCACTGTGATGCAGTGATCCTGTAGTTGATTGTTAAGATTTTG
AAGTTCAGATGTAAATGCAGATTGGTGCTGAACCGAACTTTGTGCTTTTCTTTGATTGC
CCTGAAGAAGTAATGGTCAAACGAGTATTGAACCGGAACGAGGTGATGTCAATACTTA
TTAAAAGAAAAAGTGAATGCCATATGCTTTTATGTTTTAGTTTCATTCTGAAGAGGAAA
AAATAACATAACATTTTGTTTTCAGGGGCGAGTTGATGATAATGAACATACAGTCAAGG
AGCGCCTAAAGGTATATAAAGCTATCACTCTTCCAGTAGCCAACCACTATGCCATGAAA
GGAAAGCTTTACAAGGTACTCTCTCTAAGGATGACAAGTAGTTCAAAAATGTCCAATTT
CTGCTCAACGGGCCTTTTTTATTCTCACCTCGTTTCCCTTTCATGTTGAAAAAAGGTGCT
ATGAATTTGTCAGTGGTTTTTGTTGACTTATCCTGCTCAGCTGGAACCAAGCTCCTTATT
GGTGTAGCAGTTTTTATTCATCAACAATTTCTTGCAGGTCGATGGTACTGGAACTCAGG
AAGAGATATTTGAACGAGTCCGTCCAATTTTTGCTTCATTGAGGTACATTTTGCGTTCTC
CTGCTGCTATGTTGCCAAGTATATATTTGCATCGAGATCATGATTTTTCTGAATTGATCTG
TTCCTTCTTGCAACTGTGTTTTCTACCTAATTAGGGTATTGCTCTAGCTTGATATCTTCTC
TCTTACACTCTTTGCCTTTGCTGAAAAGTATCACTATATTCAATTTGTTGTAGACACATG
TTTATGGCTCCATTTTTCACTTTAGCAGGTTGTCTACATAAACTGATTCCTACAGAAGCG
AGTGATAGTAATTGAAGAAGTTGAATTTGCTGTAGAAAAGCACACTAGAATGTAGATC
AATTCATGGAAGAAAAATGAAAAGGCAAATTGCTAGTCCAAGTTTACTATTTGTTCCTG
CTTTTCTGGTGAAAAATCCAGAGTCAAAACTGCCAATCGTGAAGATAAAATCCGCCTC
CTCTGTTGCCAATCTTTAGGCAAAGCAAACCTCTCATCCAGCAAGTTTACAGATGTGTC
TTTTTCTTTTCCCTTGTTGTTTTAATTGTTTACACATTACCTTTGCTCCTTTCATGCTATAA
CCAATATTGCGTGTACACACGCGAAAGTGTTCAAATACATTACTTCTGCAGTCAATGTT
CCTGCGAAGTTTTGATAATTTCATGAAAGATGTTGTTGTAAGCACATTGGACAGTAAAT
AACTCTGATCTACGGTATGTTGTTGTTGTAAGCACATTGGACAGTAAATAACTCTAATCT
GATTATGAGAG

>SlADK5

GAAGGGTATAATGGTCATTATCACAAAACCTTACTATGGTACGCTTCCATTTTCCAGCGT
TGGAATCAACGAAGTCCAACACCCGGAAGTGTCTCATCTCTCTGTAATAAAATAAACA
CTCAAAAAAGAGAAGAAGAAGAAGCCATTGCAGAGAATTTATCGAGGAGTTAGCCGG
AGAGAAAAATGTCGACATCATCAGTGAACTTGGAAGATGTTCCCTCCGAGAGCCTCAT
GTCGGAGCTTCTCCGCCGCATGAGGTGTTCCTCCAAGCCGGACAAACGTCTTATTCTCA
TTGGTAATAATAATAATAATAATACTCGAAGTTGGTTTTTATCATTATTTTTTCAAATCTGC
TATCTTTGTTATGATTTGTTTGTGCGTATGTGGTGTGAACTTTCAAAATTCTATCTTTTGG

CTGATTTTATGGTTTTTGCATCTAAATGGCTATCAGATCCGCATTTTTGCTATTTTGTCGG
AGTGGGGTTTTGTGAATGATCTTTTTGCTTTGTTGGGGTTTGCTTTTTCCCCCTAAATTG
CTATCAGATCTACAGTTTTTTACAGTTTAATGAAGTGGAATTTTGAAATTGGGTAGTTTA
TTTTGCTGTTGGGTTTTGCTTCTTGGGGTTATGGGAGTAGGGGGAGTTTGTGTGTACTT
GGAAACAAGATGAAAATTCTGGAATTTAGGGGGATTTTGTGGTTTAAAGGATGAGGCA
TGATCTCTGCTTAGAGCAGGTTTGCTTTTGTTAATTTTGTTAAGTGAAGAAGTGAGTGA
CGAGTAAAATTGCTCGCCTTTAATGGTTTTAAATCTAAGAAAATAATATGTGCATTCAAG
CTAAAATGGTATTCCTTAAAAGGAGTTATGAGTGAACCATGCTTTCATGTTAAAATAGG
GATTATGTTGGGGCATATTGACTATTTATGGGTTATTTGGTAACAAGAAAACCTAGCTTT
CCCGATACTTGCTAATGAAAGTTCAAATCAAGTATGCATAAATATAAAGGTGATTTCATA
GTGTCTTGTTTGGTTTTTGCTAATATTGGGAGGAAGTAGCAGTCCCCTTCTCAAGTAAA
GCTATCTCTCTATATGGTTATATCCCATGAAGATTTTCCTTTTGCGAATCTTTTTGTAATTG
AAGTTACTACCTGCTCAACCATTGCTATGGTAAACCAGGGTTCATCCTTTGGTCTCGAC
TGCATAGACTTGATTTGCTCTGCAATAATGTATGTACAACAACAACATACCCAATGTAGT
CCCACAAATGGGGTCTAGGGAGGGTAGGATATACACAGATCGTACCCCTACCTTTGTGG
GGTAGTGAAGCTGTTTCTAATAGACCCTTTGCCCAAAAGAAGTGTAATCAAAGCAGGG
ATGTTAATAATATTCACGTATACTGTGTACAAAAGTATACTGTCCACTAGTGTTTCCGTA
AGTTGTAATTGTTGCTCAACCAGTCTCAAAAGATATATGAGGAAAGGTCCTCAAGCAA
AAATTAGCTGTTATATTTTCGCATTTCAATGATTTAAGACTTTGAACGCTTGTCTTCTATG
CCCCTCATCACCACTATAACAAATAAAGTAGTTACCTAGTTGGTAGCATATGACCTTGTA
GGAGCATTGTTATTAATATTGTTTGTAGAAAGGCCATTATCAAAAAAAATATATTGTATGT
AGAAAGGAAAAAAACTTTACTAAGGATTCTCATGCTTCGGAAAGGAGACTAGACAATA
ATTGGCCTCCTATCTCATGTTGAGTCTTTTGGTCTTTTTTTATTCCCTAAGCTTTTTGCAT
GCTCGTAAGGGGGTAATGTGTAGCTGGTCGAAAGTTTTAACCATGGATCTATATATGGAT
TTGGGTTTGGAGCTAGGTTTTTCTTAATGAGTTATAACAATTTTTCTTTTCCCAATTGCCT
AGTATTTATTTCGTGTATTTTTGTAGTTTATATGGATAAATAATGTCATCCATGTGCTCAAT
CTGAATTCATTTTGGGTGTTGACATGTCATCGTACGGACTTATACATCGAATTATGAATT
GTTCTGTCATGCTTTACCAGATTTCTGTGATAGATAAATCTGTAGATTTTCCTTCTTTCCA
ACTATTATTAGTGTTAAGATCTCTAAAAACTTTAAACAAATTCTGCAGGCCCACCTGGAT
CAGGGAAAGGAACACAATCTCCTATCATTAAAGATGAGTATTGCTTGTGTCATTTAGCC
ACGGGGGATATGCTGAGAGCTGCTGTTGCTGCCAAAACACCTTTGGGCATTAAGGCGA
AGGAGGCCATGGATAAGGTCATATTGAGTGTTCTTTAAATTATTGTTATATGTTTCTGAC
TTCTTTCAAGTTCCAACAGGAGAGTTTAAGGGAGAACAGCAATTGGTTTTCCTTTTCTC
AGTAAAATTGTTTTTGTACTACTATCTTTTCAGGGAGAACTTGTTTCTGATGACTTGGTT
GTTGGGATCATAGACGAAGCATTAAAGAAGCCATCATGTCAAAAAGGATTCATTCTTGA
CGGGTTTCCTAGAACTGTGGTGCAAGCAGAAAAGGTTATTTTCCTATTAATCCTTATTTA
TGTGGACTTTATGTGCAATTCCTATCAACTTTGATTGAATCCTTGTAGTAGGATTATCAC
GTCTCGCTCTACTTATTGCAGCTTGATGTGATGCTTCAAAATCGAGGTACTAAAGTTGAT
AAGGTTCTTAATTTTGCGATTGATGATGCCATCTTGGAAGAGAGAATTACCGGACGCTG
GATCCACCCAGCTAGTGGTCGGTCATACCATACAAAATTTGCACCTCCTAAAGTACCTG
GGATAGATGATGTAAGACAATAGAATCAAAGTTTTTAGTTTTACTTTTTGTGATAATACC
ATCTCTTCCCTCTAACTTGTCAAAAACAAAGTACTGTAGTTTTTTGTTGCTGAAGGGAG
TTCATCGTAGTGTGTCTTCATACGCTTAGCAATTGATTATTTTACAATTTTAATATCTTTTT
TGTCTGCTTTGCAACTTAAATTCTGTCAGACAAATTTACCTTCATAATAAGGATTGTCAA

CCAAAAAAAAAGGAACATTCTATATTAAGGATAGTTCTCTGATATCTGAATCTAATTCAA
TTATATCTTTCTACATGAAGGTCACTGGAGAGCCATTGATACAACGAAAAGATGATACT
GCTGCTGTTCTCAAGTCAAGGCTAGAAGCCTTCCACAGGCAAACTGAGCCGGTAAACA
TCTGTTTATCTCTGACTTATTGAGGGGAAAGGGAAAGTAAAAAAGAACGATAAACATC
TGTGTATCTATCTAGTCGCTTTGGTAGAGAGTTGGGAAAACTAGGAATTGCATTTGGGA
ATTTGACATAATAATCTATATAAGATGTTTGGCAACATTCTTCCAAACATTATATACACGG
TATAGCTTTCATTTGACATAATAATCATTTTTGTAGTCTGTTTGGACTCTTCGTTAGTTTC
TGTCCTCCATGCTAAAGCTGTATTGGAATAATGTACAGGTCATTGACTACTATGCCAAGA
AGGGTAATGTAGTGAATCTTCCTGCTGAGAAACCACCACAGGCAGTCACAGCTGAAGT
CAAGAAAGTTCTCTCCTAATGGAAGGATGCCAACTTTTCATTTTGGTTTAAGGATTGAA
AATTTAACTATGAATTTTGGTTGACCAGTTATATTTCTGGTTTCCTTTTTATGGCACTAGC
TGGTTCAACCCTTTATGAACATATCACTAAGTGTTGGTGAATTTATTTACTTTTGACATT
GCTCTTTAGTGAGCTTTTGCAGAGTGCAATAGGAATTGACACAAAATTATTACTCGTAC
CAAGTCAGAATGAAGTTTAACAGAAGATACCCATTTTTGAGTTTTGTGGGAGGGTAATA
GAAGC

>SlADK6
TTGTATTGAGTATAGTTGGAAGACATAGATCCCCAGTCCTCTCGGTGCTGTGTTGAAAC
CCATTATGCTTGCTGACATCTTTCATGGTCGGCATTGTCAAACCCCCATTTTCCCTTTTC
CTTTTCCCCACCCTCCTCTTTCATTTTCCTCTTTTCCTTTCCTCAATCACTCTTACTTCAA
TTCAATTCAATCATTAATCTCACAAAAGGGTAAAGATCCATTGATTAACTATGGTGGTTT
GGACCCGTGCAGTGGTAAGAACATGGAGATGTAGACCCACCAATTTTAGCCGGGCTTT
TTCTGAAAAATTACCTACATCGGAGCCTAAGGGCAGAAATATTCAGTGGGTATTCCTGG
GTTGCCCTGGTGTCGGAAAAGGGACTTATGCAGCTCGACTTTCTAAGCTTCTTGGCGTA
CCCCACATAGCTACTGGTGATCTCGTCCGTCAACAATTATCCTCTCATGGTCCTCTTGCC
TCAAAGGTTGTGCCCTTTTTGCTTTTTGAATTGTTTTGGGTTTCTATTAAATTATTATGTG
TAGCTGTGAAATTTATGTGATCTTTTGCCTAGTTCCCTCGGATTTTCATATGTTTGTGAAA
TCTTGAAATGTTTACTCTAAGTAATTCTGGGGTTTGTATATTTGAAATTTATGTGATGGAA
TGGTAAAGTTCCATCTTTTGCCTAGTTCCCACGGATTTCCATATTTTTGGTAAAATCTTG
AAAATTTGTGCTCCAAGTGATTCTGGGGGTTCTATTAAATTGTGTGTATCTTTGAAATTT
ATGGGATGGAATGGTGGTAAAGTTCCCATCTTTTGTCTATTTCCACAGATTTTCGTATCT
TTGTAAAATCTTGCAAATGTAAGTCATTTTCTTTTTTCAGCTATCTCAAATCTTATCCAAT
TTTACTTCTATTAATCTACAATTTATTTTCTTTTTAGTATCTAAATATATTACGAAACTACT
AATCTCATAGCTTATCCTACTTCTGTGCGATTGCTATGCATATGTCACCTACTTTTGCACA
TTACCAAGCACTAGAAATTGATCAAGACAATCATTTCCTAAGGGATATCATTTTCCTCCA
CTCCAGATGCAAACATCTACTGGATTTGATTTATGTGTGCATGTGTAGATACAGTAGTCA
ACTGAAGATTACAAATGGCTGATCTTTTAATACAAGGACCATGATACACTTAAAGGGTT
CCAAATGACTGACCAAGTAAGGGATAAGTTGGAGATTTGCTGTTACCCTAGTCATTTGA
TTTGTTCGACTGACACTTTATAAATACAGTTCCATACAAATGCTTTATGTAATAATACTGT
ACCCATGAGTCAATGAAAATGCTGCAACAACTCTTATAGCATCTTATAAAGTTATGCAAT
AGCTAAATAATAGGGGAAGTATTTGTAAGTATGCTTGCATTAGTGGTGTGAGAGATACA
TTTATCAATGTTGTGAGAGATGAATATGGTATTCTCACTTTTAGATGTATATATTAGGTCT
TTTACCATATAAAAAGATTCTCTTTCTTAAAAGTCAATGTAAGCTTATTTCAATGACTTTA
AGATTGATCTTCCCGACTTTGGACAATGCAACCATTTTAGGAGTATGTTTAAAAAAGAA

TTATCATTTGGAAAGTTCTTAATGTTTGCGTAGTTAATACTTTTGTATCTTATTTGGACAG
TAAATAAAAACTAGAAAAATCTTTTTTTCTGGATATCTTGTTGCTTTCACATGAAGTTCT
CCTGAAAAAAAGTTACCAGTACTACAATAAAAATTATGATGATTCAACCCTAAACTAGT
AGTATGCGAGACGTGACTTGGTTCCCTTTTTTGTCCTTCATATATAGGAGACATGAGTTG
GTTCTATTCTTTCTTTTGGGTCCATTATTTCATTGCTCAGTAATTTTATTTTAAAGTACCTA
GTAGATTTCCAAGACTAGAGATTCTCTAAATTTCAATTATCACACTTGTTCCTACACCAA
AATGTAAATTTTAACCAAACTAAAAAAGCATATTGGCAGACACTGGACCAAAGGTAAG
TACCAATAACCAGTTAATCTTAATTCTAACTAGTTGGTATTGCCTATATGGATCTTTGCTT
CCATTGTATGTGCTCTTTGACAAAACCACATAGATTTGAGGAGATTGAAGGTATTTTGA
GACAACTTTGTTCCATGTGGGATAGCTCGATCTCTGTAATGCCTTGAATCATGATAGTGT
TATGCGAACAATCAGCACAATTTTAAGTACATACAACATGATTTGACCATTATGCGTTTT
TCTCTCGTTGTCTCCTCGATATGTGCTACTTGCATCTTTGTCAGATATGATTATTTCTAATA
TCATTTATCCATCCATCTTAATTTCTATTAAATACTTATGTGTTCATATTTCTGCTGTAGTG
CCTTTTAAACTGTATTTTAATAAAATAATTGTTTCTTTGATAAGTTGAAGGAAAGTCTATT
TCTTAGAGGCAGTGATGATATTGTTAAAAAAAGAAAAAAGAGTCTAATTCTTAAGCTTA
GATGTGAAAATATGAATTTGTATATATCTTCTTTATTGATTATGCCCTTCATTTACTCCACT
GTGCCTTTTCCCCCCCTAAAGTCGTTCTACTAATTTCTTTTCTGAACTTACTTTGTGCCT
TCTGTTGCAGCTTGTGGATATTGTTAGCCAAGGGCAATTAATTTCAGACGAAATTGTAAT
AGATTTGCTATCTAAGCGTCTTGAAGCTGGAGAAGCAAAGGGTGAAACAGGATTTATT
CTTGATGGATTTCCTCGAACTATACGGCAGGCGGTAAGCAGTTGTTTGTATAATGGATTT
CCTATGTCTAGATTGATTGTTGCGCTTCTGAATAAATACTTCCCATGTTTTTTCTAGGGAA
TAGCTTACGAATCTAAAGAAATACTTACATATAAATGTGCATACACACACAGATATCTGT
GTATTCATGTGCTATTGTCATGTTTCTTACATGTGTGTATTATTTTATTTGACGGTTGCAC
CTGTTGACTGGGACTTCATCTTGTGCATCTATCTACTTTAATAGGCCAATATTTTCTTTCA
CAGTCTGGCATCTGGCACGCAGGGGCTTGCTATGGATATAACCTAGCAAGATAATTTATT
AACGTTCTAATGTCAGTACTTGAGGACTGTTTCACAACTAGCAGGGATGCATTATTATA
ACAGGTTAACGGTTATGGGCTTATAGCTGGACCTGCAATTGGAATGCCGCTTGCTCAAG
AAAAGTTGAATATGCTGTTGAACGAAATGAATGAATAAAGTACAAAAGGTTGAAATCA
ACAGCAAAGCTCGATATGATTCTCTCAATAGATTGTGCGGTCTGTGAATTAAGCTCTTA
GTTGAGGTGATAAGATCCTAACTAGAGTAGATATACTTCATCTTACATGAGATGCATTTT
ATTTGTATGATACATCAAAGTTTCTGCAAAGAGCAAAACTCTGAACTGCATAATATGTT
CATCAGATATATTAGCCAATTCTTATGTTCATATGTAGATAAGGGAAAATGTCTGAGCTA
ACAAGAGCAAAGATCTGGCCTAAGTGTGTCATATTAGTTGTTTCTTGATTTCTTCTAGTG
ACACCTGATGATTGGGAGAGACCTTGTTAAACATTGACCACGGTTAACCATTCTTCAAC
TTTTTACAATTAGTTTCAAAATTTGAGGCATGTTTGAACAGCCATGTACATTGTCTTATAT
GTTCTCTTCAATTGCCACTAGAAACATTTGATATTGCTTGTCATTTTTTTGTATGTCAATG
CAAGTAATGGCCTTGAAATTTCTAAATTATGTCCTCGCTAGGTTTTTCATTGCTGCGTAG
TCTTTACTAACATTAACCTTTTCTCAGATGTACCTTGTTAAACGGCTTTACTTAGCTCTC
AGATATCATCCACAATTGCCTCTACTTAATGGAGCATCACATCATGTACATGCTTTGATT
GTTTGACTGGCATAAAATGTTGTGTCGCTACTCCTCTATGAGCATCAGCTGTAAATTTTG
CTAACTAGAGAAACAATGTTGAGTATACATTCTAATTCTCTTGGACAGTCTTTGAGGCA
TGGTTTTATTATCTGCTTCTCTACCTTTATATGACTAGTATGAGACCACAGGGAAAGTAC
AATCTAAGAACTGAATGCCCTTGCTTGGGTTTTAGCTTTAATGGCCATACAATTGTGGAT
ACTTTAGTTACAAAAGGCTCATGTGATGTATTTCAACGTATTGTGTTTGTGATTTTAACA

GTCACAACTTTAGGGTTTTCTTGGACTGTTGTGGCTGTTATATGTTCCTCTGTTTTAATT
AGAAGCTTAACTTCTTTATTTGGTAGAATCTTTTAATTAGAAGCTTAACTTCTTTATTTGG
TATAATCTTTTCATTAGAAGCTTAACTTCTTTATTTGGTAGAATCTTTTGATTAGAAGCAT
CTTTCTATTGGTTCTTAATCAATATTTTGTTTGTGGATTGTTTTATGTGATGTTATGTTTAT
TGTATTATTGATTGCTTCACCAATGTTTATGGGATTTGACCAATTTCTAAATTTGTGGAA
GCTGCCAATAATGGCAGGGGATCAATTTATCATATGTCAGAAAGATGAACTATTAGAGG
ATGTAGTCAACTTACTGCCTAATGTAGGATTGCAAATCTTGTAACCTACATCAAAAAGT
GTAGCTCCCTTCAAAAACTTACAACATGTGCCATCTATCAGTTGCTCCTATAAGTAGTCT
AAAACATCAAAAATCTCTTCTGTTTGAGCTATTAGATTCTGTTTACACCAACAATAAAA
AAAGCGAGGCAGATCATCTTAAAGCCCTGTAAGTTGCTCTGCTTGTCTTCAAAACATCT
CCTTTTCCTTTCCTCCCAGACTGTCCACCAAATGCAAGCAGAGACAATTTTCTATCTCT
TTTCAAAACCATTTTGAGTCCTGTTGTTCAAAACTGAGAATATCACTGTGCTGATATAAT
GATTGGCTAAGCTGCTCGGACTCTTCACTTTTGGTGCCTCACCCATGTCGACACGACA
TGGGTGTGGGATCCTTGTCCGATTTGGTAAACGGATTTTTGGTACTTTGACCAAAATCG
TTGGGGAAAGTCTGGACAATTTTAATTATTCCTATAATCAATACAAAAGCTAAGGTGAA
ATTGAAGGAAATGGAATACCTTTTATATGGAAGTTTCTATTTCACTCCTTTTCCTTTAATC
TCCTTCCGGGATTCTCCTCAAGTTCTCTACATAATATGTCATAATCTGGTCTTTATAACTT
TATTTTTAGATATTTTAATTATTTTAGGCGACTCCCTGCACCGTATCATACATGGATCCTAT
CCGGGAATCTTAAAAATTAGATCATGAAGGATCCGACCTCTAGATCCACACTTGTACAC
CCGCACCCGAGTCTGAGCAACTTAGATGTTTAGTCCACACAATCCATCTTGTGCCAAAC
CCCATTCTTTGAAGCATATGGATGAAGGAGCTCCAGTTCAAGTGATATATGTTTAGTGGA
TATGTAGTTTGAATAAGATACCTGGATTTAGGCTTCTTCGTCTGTTTCCCCTGCACTCAG
TGGCAATTAAGATTGCATCCATGATCTTCCTCCCAATGTAAGGATCTTACATTTTTGTTT
AACCCTACTCCTTTTGTTCAAAAAGGTAATATGGAAGGAAGTAAGGAACATGAGTATCC
TCTTCCCCCAAGGGTAGGGAGAAACTGTTGGTAACATTAATGCTGATATCCCTTAGTTG
TGTATCCTAAGGAGAGGATCCTCATGCTCTCCTGATGATTGTTTTTTGGTTGATGCACAA
TGTTTGAATCGATTATCTGAAGGGCAAAAATTGAACACTGATGAAGATCAATGTAATAA
TTTACTGAAACTTCAGGAGGCTGTAAAGTAGAGGTACGGCCAGTTATCTTTTGGCAATG
CTTTATGCAAAGTCAGGACATTGATGTCTTCAGTATTCACTAGTGAAAAAAGGTTTCCT
GTACTAGGGTCTAGGGCTGCTTGCAGGAGTGTCTCCACCAGTATAGAGGATCATCTTTG
TTTCCCTTGTTCTTGGGACCAAAAGTTTCCAACACAAGCATTCCCCTGCCTTTCTTTCC
CGGCTATGTCTGCTTCTTGCTCCCTACGGTGTAGTAAAAGTCTGTTTCATGTGGTGTCAT
GGACCTGTATTCTCAGTCTCTTCTACTTTTTGATACTAAGACGTGGACCCTTGCTCCCTG
AATTTGGCTGGTCTGGTCATATCTTTTGTTGCCATCAGAATAACTCATATGATTACTAAGT
TCTGGTAACAAATTAGAATCCATGGGCAAACGCTGAAGCTTATGGTCTTATGTTTGAAG
GCTAAATTATTTATATATCTGACTTTGGTGGGTTCACCTTACTCTCTGTTGTATAACTTTT
TGTTTTATGCCAAGCAAAACTTTTACTATAATTGAAACTCTGCAGTCCTTGGTTCCTATA
TCTGACTTTGGTGGGTTCACCTTACTCTCTGTTGTATAACTTTTTGTTTTATGCCAAGCA
AAACTTTTACTATAATTGAAACTCTGCAGTCCTTGGTTCCTATATTTGGAATTTTATTTCC
CCTATAATTGGATCTAGAGATGAAACAGATCTGATATGTGTACAGGTCCAAGCAGTGAA
TTATCAACAGTGTAAGTACTGCTCCCTGATAGTGGCTAAGCTGCTTTGTGCACTGAAAT
TAATAATTTTTTTTCCTCCTTTAACTAGATATAGACGGCATTTTCTCTGATCCTGTCATATG
TGTTATTTGGTTATTTAGGAAATCTTAGAGGGAGTGACAGACATTGACTTGGTGATTAAT
CTGAAGCTTCGGGAAGATGCGTTGATTGCCAAGTGCTTAGGAAGAAGGACTTGTAGTG

AGTGTGGAGGCAATTATAATGTTGCGTGCATTGATATGAAGGGTGATGATGGAGAAACT
AGAATGTACATGCCTCCCCTTCTCCCTCCTCCGCATTGTGAAACCAAACTTATTACACG
GTCTGATGACACTGAAAATGTTGTGAAGGAACGCCTCCGCATCTACCATGAAATGGTAT
GATCTACTTTTCTCTTATTTATTGTTTCTCGCTTTGTGCAAGCGTTCTCTTCATGTACTTG
ACTGGTGAGTATACCCCATTCCTACGTTACAACTTCTTCACACTTCCGTAGACAACAAA
AATCTGTGCCATTCACAACTCAAGAATTGATAATTCTGAGCACTTATGATGTGTTTTACA
AGCAGATCATCAAATCATTTACCCAAATTTTTGTGTTTTCTTGTAGTACGTCAAATCGGA
AGATAATCACTGTTTTTGTCATCTGTATACAGTCAAAAGTGGCATTGAGATACTCTATAA
CCCTTTAGTTTTTTTTCCTCCTGTCCTCTATTTAGCCTGTCTCGACTAGTTTGGGGTTGA
AGTATAGTTGATTGGTTGGATTGCCCTAGAGTTGCTATTGCATCTGATCCTTCCAAAGTG
GCTTCTTTTGTCAATATGTGCATTTCTTTTGTGCAGTGGTTACTAACCTATTGTTGTATTT
CCTGTAACTGTGTTTTTTCTCCCCAGACCCCAGATCCTCATTTGTTTTTTAGGACGAGAT
GGTACAAACTTAATTCTCAGTTAGCTACTGTGACCACATTCATAATTTACTTTCCTAAAT
GCTTGCCCACAAAGATAGGGATAAAGTCTACGTATATCCTACCCTCTCCAGACTTACAC
TAGCTATGTTGTTGTAACATACTAACTGCTTAGGTATTATGTTGTTCCTGCAGAGCAAAC
CAGTAGAGGACTTTTACCGCCAGCGAGGTAAGCTGTTGGAGTTTGATCTCCCTGGAGG
AATTCCAGAGTCATGGTCAAAGTTACTTCAGGCTTTGAATATCTACGACGACGAGGATA
AGAAATCTGCTGCAGCATGATAATTGATCACTTGTCTTGTCAGTATGTTTACTATTGTTG
TAGGTTCATTCTTTGCATACATGTTGGACATGCACAAGCGTCTATAATGGGGCTCCCTAC
CTCCTACTCTTCTCTCCCTCACTATTGTATCATATAATGCTAAGGCAACTATAACGATTGT
ACGGAGTATTTCTTGTCCATATTTGTTCATAAAGCTTAGCAAACCATGTGATTTCATTAA
ATATTTTGATGATATAGTAGGGGAAATGTTATTTAGATCTTATTACATAGTCTGGTAAGTT
CATATGCTCAAATAAT

>SlADK7

CGTCATTGACTTTCTTTCTACTATTTGCTTGTTACAAAGTCATTTTTGTGCATCTATAGTT
TGGTGTACCTACCATTACCAAACCCAAATTCCCATTACAACAAGTGTTGTAGGGGAAGA
AGAAGAAGAAGAAGGCAAAGCATCAACCATGGCTGCTTCATTAGAAGATGTTCCTTCA
GAAAGCCTAATGTCTGAAGTTCTCCGCCGTCTCAGATGTTCTTCTAAGCCTGACAAAC
GTCTCATTCTCATAGGTAATTACTTCTTCAATTCTATTTTGAAATACTCTATTTGCTTTACT
CGATCAGCTTCGCCTCTTTCAGTATAACATATAGATATTTTGGTGGAATTGGACTAGTTA
CTATGATTTTTAATTTGACTTAAAATATTACATTAGTCATAGTTGAAAAAAATAAAATAA
AAAGTTTGATTGAGAAAATACTGCTTTAAAGTTAAAAGTTAAAATTTGAAGGTGTGAA
AGTTTGCATAGAAATTGAAAAGTGTTGGGCCTTTTGGGATGTACTGAAATGGAAACAA
TTTCGGTTTAGTTGGAACGAGTATTTAGTATTGAAATGTAATTGGCATGAATAGAGTATG
AGGGATACAAAATCTAAGGATTCAACCTAATTTGTGATTATACTTGCTTGATGGGGACT
GTAAACTGTATCTTATTGAATGTGCCAGAGTATTTTAGCTGGATGAATTTGGTAATGCAG
TTGTACAAATGAATAAAGCTACATCTTTTAAAATACATTTGGCTAAAGACTGTAGAGCAT
GGAGAAAGCTTTTCCATTTTGAGTTTCGTGTTTTCACACCACCTACCCCAACTAGTTTA
GAAAGTTTAGAATTGAGGCATTGGTGTTTTATGCGTCAAACACTACTTTGTACTGAGCA
AAGAATAGATCAGATTATTGTGATGAACCGAAGCAAAACGAGGAGTTATTGTGTCATG
GGTGATTCAGAAAAGTGTGCGCTTCTAACAATATGTGCAAAGTGATTTAAATGAGAATC
CGTTGGTTCTTTGAATCTCAACTTTGAATTGTTATCGTATATTGGAGGTTTAAACTAGTT
TTTTTAATTTTGCAATTTGATTGTTATAGTACTAAAAATCATATATGTTTGGTATTATTTTTA
TTTTTTGTGAATTGTGTGCTTCATTTCTCACCTGAAGTCCTGAACTCCATGGACCTTGTT

GTTTCTATGCACTTTTTGCTGTTCAAAATACTGATATTTACAGCTTGCTTTTGATATCCAA
ATGGGCAGCAGAAACTTGTTTTTGTTGAAAAATTAGCTATTTGGGCAGCAGAAACTTG
TTTTTGTTGAAAAGTTAGCTTCAATCCGTTTAAAGCAACTATTTTTGTTAATTTTGTGCT
TTTATTAACACAAATGGGAAGTTCTATAAATGCAGCTGCTGTATTAAAATGCAAGATCA
AGAACAAGTGTTGTTTTTTGACTTGTACTTTCACAATCTTGTGCAAAATGTTTCATAATA
TGTATCTCGTGTAATATTCTCCTTGCAAAGCATTGATGTTGTTGAAAAAGTGTTCACCGG
ATTCTTTGACATTGCTATGTTGCCATTTTTCTGTTCAAATTTTCGTAATATCTACTTCTTC
AGGGAGTTCTACATTGATACTTAATGAAAAAATATTGTTATCAATTGTGGTGATGAAGTG
ATTTGTAAAGTTCAAAGTTAGTGCTAACAATGTGAAACTAATGGCAGGTCCACCGGGA
TCTGGAAAAGGTACACAATCTCCTATCATAAAGGATGAATACTGCTTGTGCCATTTGGC
CACCGGTGATATGCTCAGAGCTGCTGTTGCTGCTAAAACTCCACTTGGGATTAAGGCA
AAGGAAGCTATGAACAATGTAAGTGGCAGTCAGTTTTGGGGATGGGTAACAAACTTCG
TAATGTTATTTCAAGTATTTTTCTCTTTTGGTGAATGATGTCCTTTTTGGGTGATTTTTAC
AGGGTGAACTTGTGTCGGATGACTTAGTTGTTGGTATAATTGATGAAGCAATGAAGAA
ACCTTCATGTCAAAAAGGCTTCATTCTTGATGGTTTCCCAAGGACAGTGGTTCAAGCA
GAAAAGGTTGTCCAACACTATGAAACATTTGCATCGTGACTTCTCATCTTATTAGTGTTC
TTACTTTTAATTGTGGCGTGATGCAGCTAGATGAGATGCTTCAGAAGCAGGGGTCCAAG
ATTGATAAGGTGCTCAATTTTGCAATTGATGATGCAATCTTGGAAGAGCGGATCACAGG
CCGGTGGATTCACCCTTCAAGTGGTAGATCTTATCACACCAAATTCCAACCCCCAAAAG
TTCCTGGCGTCGATGATGTAAGTTTGCTAAGTTGCCATTAGTTTTACGGAATGCTTTTGT
GTGAATGGCAAAGTATGGTTGTCATACCTCTAGTGGCTGTAGTTTCTTTATCGCTTTGCA
TACATTTGCTGAAAACAAATTATACTTATTGGTAATTAACATTGACCTCTCTATTGTTTCT
AAACTGCGGAGAAATTTCCTGGGAGTTCAGGTTCCTAAACCAGAAAGTGGTGGTAATT
AAAAACCATTTCTTTATCCAGTTTCTAAAAATAATATTTGGGGAGTTTAGGTGCGTTAT
TATGAGTCTCCTGATTTTATTTACTTGCTTAGGACGTTCTTTCTCAAGGCACCTTTAACT
TGTTCAATAATTAGCTGAAGGCCGGTAGTTTTTGACCTTTAAAAGTTTTCCAACAAAGT
ACCTGGTAAGATGGCTTAGTTTTGGCTAAAACTAGTTGTTGGTCTTATGTCTTTCATTTT
CAACGAGATCAGTGTTGTAAAAGCCATTGCTGGGTCGTGTAAACTGTAAAGTAATGAA
GCAATGCAAAGGGAGGGGTTATGGCTTTCTGGGTGAAGCCATGCGCTTCAGAGAAATG
GGTGCTTCAAACATTGACACACATATGTACTTTACATTTATAAAAAAAAATTACGCGCAT
AGGGGTCGTTTTGGTAGGATACATTGGAGGAAATAATGCATGAATTAGCTTTGTATATTA
CTAACACCTTGTTTAGTACACTTTTTCAACCTATGTATAACTAATAGAAGCATTATACACT
CTATTTGGTATTATCCTATGCTTTTCTGTTTGGGTTCTCCAAAAAGGATGTTTCTCCGTGT
CGGATCCTTGGAAAATGTACTACTTTTCAAGGATCTGACATGTACCCGTCGATATTTTTT
AAGAGTTTGGTCAACAAAAATGCTACGCATAATAACTAATACACGACAAACTATGATAT
TAGCAATGCAAGAGTTTTTAATGCATGCATTAGAAGGGGTTAAAGACACAATGCCCTCT
CAAACCAATTCCACATCCTCTCCACGATATTTGTGGAGGATATTTTTGTAAACACATAGT
TTGTTTTAGTAATTATGCAATGCATGTTATTATCAATATACCAAACCAAAAAATGCATAAG
AAATGTTCTTAGCATGACAAATGTCAGTATTATTGAGACACCCCTATGCCTACCAAACA
ACCCCACAAAAGAGAAGCTGTCAATTTTTTGAATCTCAACTCTCAGAAGTTAGAACTA
ACGTTGGCATAACTATAAATTCAATGCTGAAATTTTGGATATATTTTTATTTACCGTAAAT
TGTGCGCTTTATGTCAAAGAAGCTTTGTGTAAACTTCTTGCCTTTGTTACAATTATAAGC
CTTGTGCTTTTAGAAACATTGCCAACAATTAACCATTTCCCTATCAAGAGCTCGATTTCT
CGAAACAAACAAAAAGGAAACAAAAGATGTCAAGATAGTCTCAAGCCTTCAGTTATG

TTGCTTGGGCACAGATATGTATGTAATAGACATATTAACGATTAGGAGTACATAAATTCA
AATTAACATAGAGCAACTTAGTCAACAGTAAAGGAAAAATAATCATCAAAATCAATTCT
GAATCTCAAATTTTAATCAGGGACCCGTCGTTCTTTAGTATTTCCTAAAAAGAAACAGT
AGTGTATTTGAATTCATGTTGTAGCTAGAGGGGATCAATGATATTAGTTATTCCAAAATAT
CATTGAGCGGTTTACTGATGTATCTTTCTACATACAGGTCACTGGAGAGCCTTTAATTCA
ACGGAAAGATGATACTGCTGAGGTTCTTAAATCAAGGCTAGATGCATTTCACCGTCAAA
CTGAGCCGGTTCGTATTTACAGTTCATTCTTACTTCCTCTTAGTATTATAACCTGTATGTT
TTATACGTTTCCGTAAGGATAATCAGAATTTTAAAATTCGTTTTTAACTGTGAATTATCTA
GTTTTTATTTCTAGACGAACAATTAATTCTGATATCCTCGACAGCCGGATAACTTTTATGA
TTAAGATATATTATTTGGTTTCAAGTTTCGCTGAGTGAGCTTGAAGAGTCTATTGGAACA
AAGATGTAGAATTACTTGACTATGAAATTGTGGATTAACAATTGTGGCAGCTACCCCAA
TAGGAATAGATGCATAAAGTATCTTACGATAACCTATCCATTTGAGTATGAAAAACTTTG
TTATACGAGCTCTTATCAACATTTTATGATACGACCTTATATTTGCGTTATGTGTGCAAAA
GGAAGTCGAGTTTATTATGACCATTCAAAATTGTTTGATAATGCAGGTAATCAATTATTA
TTCCACCAAAGGTGTTGTTGCAAGTCTTCATGCTGAGAAACCACCAAAGGAAGTTACT
TCTGAGGTTAAACATGTGTTGTCTTCTTGAAAAATATCATCTGTCATAACAAAATTGAG
ACAATTCTTTTCTTTGTATTATCAAAAAAGAAAAAACATCCTTCTGATTTTTGGTTTTCT
TATCTGCGGCATTTTGTGCAAGTCCCTGAGGAAGTATTGTTTTTATGGGGAATTTTTGCT
AATAATTTTAGATATAACTCTGCATTGGAACAAATAATCCATGTAAGCCAAATCTTGATT
TGGGATTTGAGCATAGTTAATTGATATGTGATTTGAGAATTATGTTTGGTTGTTGCAAAA
TGTAGATTATGATTCACCTTGATTAATGCTTCAAGAGGATTTATTTCTTT

>SlADK8

TGGAGCCTTTAACCCTACAAATTGTAAAAGAAAAAAAGAAAAACACTGAGATCTCTAT
TTAGCACGGAGCAGAAGCGCCATGGCTTCGTGCTGTTCATTGAGCTTCTCAACAGTCT
CTTCAAAGCCTAACAAGCCTTACTCATCACCAATTTCTTCTTCTCTTGAGCTCCCCTTTA
CTTCCCAGTTGCCATTTTCTAAAAAATATTCACTTTATTCCAATCATACCCTTCTCCAAA
CTCAATGCCGGAAAACCCAATCACCGGATTGTCCCAGTTTCTTGGTACGTCTTGCAATT
CTTTTGACACCCTTTTGATTTTCACGTTCAATTTTGAGTTTAGAAAAGTGAGCCCAGAA
TTTGAAATTAGTAATAGGTTGTATTGAACATAAATGTATATTGAAGAGTTTTACTACGCA
GCTGTTGGTTTACAATATATTTCATGCTCAATCGAATGAGATGACTTGAAAGAGTTAGA
CTTTAGTTTGGGACTGAGATGTAGTTGATTGATTGTTTTTTTGGTATGGCTTTGTGTAGT
GGCTGAGTTTTAGCCAAAAAGGGAAACTTGATTAGTGATTGAATTGTATTTTTGTGATT
TAGGTTGTGGGATCTGCGAAAAAGCAAGAACCTTTGAGGGTAATGATATCGGGAGCTC
CTGCTTCTGGTAAAGGAACGCAATGCGAGCTCATTACCAAGAAGGTAATGGTTCTTTTT
TTTTTCTTCATAGGGACAAGTTGGTTGTTATGATCTTTTATGTTTGAATTATCTTTATGCT
GTTCTATGTAGGTTTCTCTTTTTGGAATGGATGATGATCCATTGGGATTTTGGTTTTATAA
TTCTGAATATGAAATCTCTATTCTTGAAAGTTTATCTACTAATATAAATCGTCCCAATTTC
CACTAATATGTGTGTTATAGACCAGGTTGTACAATATAGAGGTATACTTCCATTCATGAC
TTCTGACCATGGGTGTTTCTAAATTTGAGGACCAGTTTTTTACCATATTACTGGTATCATT
CTACCCAGGACCTCTTATGACTTGACCCTTACTCCATTTGGTTCGTAGACTGATAACAC
AAGAACTGGTATGTGGGGATTGTTATACACTGATTGACAATACCGGCATGGTTAATACC
AGTATTAGCTATGCATGTTGTATATCTAGTATAAGTCACAGGGTGATACACCTCTTTTACC
ACAGCCTAAAAAAGCAGAATTATCAGATTTATCTGCACTCTTGTAAACTAAAATCATCA
TGTAAATTTGTCCAGTTATCAGATTTGTCTTGAAAAGCATCTTACTAGCATCTGTTCAGC

TGTTCCATATATTTACGTTATTTCCTTAGCATAACATGTATGTCTCCTATGTGTATTCTCTT
ACAATTCACTGGTACCTTGTTCCCTAGCCTCTTTATCTTGTTTATTATGAATTCTCATCTG
GTTCCTCTAAATTCTCATTGGTGGGTTTGTAATATTTCCTTCAGCCGAAATTGTCCATATT
ATAATCAACTTTATCTTAATTATTAGCAAGTCCCTGCTACTTGAACTCACATTCTTCACAT
TTCTTGATTGATTTAGCTTTAGTTTGATTTCCATGCTCCTTATTCCTTCCCTTGTTTAACAT
GTAGTTGTAAAAAGGTATGTTGCTAGTAGCATTTGATGTTCCATTTCAAAAAATGTCTCT
AGTTGCAGTCGATGTTAGTTGCTACCTTAATTACCCATTTTCCTTGTTATGTATTACCCTG
TGAGAAGTAATAAATTGAATACCGTGAGCTCATATATGCAGTACGATTTGGTGCATATTG
CTGCTGGAGATTTATTGAGGGCTGAAATTGCTGCAGGCACTGAAAATGGGAGGAGAGC
AAAGGAGTATATGGATAAAGGACAATTGGTACCAAACGAGATAGTTGTAACGGTAGGT
TTACCTGGCAAACTGGTTCCTGCTTTTGTTAGCATGCTATTTTAAAGGTTGAAAGAGAT
CATTAGAAAGAGTGTTTTATTCATATGTGATTTGTTGATTCCTTGTTTTAGATGGTCAAA
GAGCGGTTGATGTGTCCAGACTCTCAAGAAAAGGGTTGGCTTTTAGATGGATATCCTC
GGAGCTTGTCTCAAGCAGTAGCTCTCAAAGAGTTCCAGCCAAACCTTTTCATTCTTCTG
GAAGTGAGGTTCTGCTATTGATATCCTCTGATTGTGTTCTCAATCTCTTTCTAGTTAAAG
CCTTAAGAGGATGAAAAGGATCAACATAGAGTATCCTGCATTTAAAATTTACCTGAAAA
AATCTTTGAACGGGAAAACTGTGCTTTTGGTCTGTGAAAACCTAATTTTAGCCTTCAAT
TGAGAGAACTGTGCGCTTTTGGTTCCTCCTCTAGCATATTTCCCTACAAGAGTCAGATT
ACTTCCTTAAAATGCAAAAGAGTATCAAAATAACTTTCTTAGCATAAAAGGTGAATAGT
TGTATGGTTTAATAGTTGTACTTTCATGGAATTTATCAGTTGAGGGACCAAAAGCTGATA
CTATCAGTTGGAGGTTTAGTGGGCTTGACTGAATACTGTGATTCGTTAGTACCAAGATT
ACAACTTACAAATAACCAAAAAAAAATTATTTTTTTCCTTATTTTAAATATGATTAGTTTT
TTCTGATTCTTTTTTGTTTCACATGTCGTATATTAAACTGCAAGAGGTTTCAGAATAAAT
GCACCTTCATCGAGTCATCAACCAATGTTTTAACTTTTAACAGGAACACGTTATATTGTA
AATGATAGGTACCTGAAGAGATACTTGTTGAGAGAGTGGTTGGCCGTAGACTAGATCCT
GTAACTGGGAGAATATACCATTTGAAGTATTCTCCGCCAGAGACCGATGAAATCGCTGC
AAGGCTTACCCAGCGCTTTGATGATACAGAAGAAAAGGCATGTTAATGCATGAATACGT
ACATTATTGTTGCTACAACTGCTGTAGGGCTTTTCCTTTTGTATGAAGCTTTTAAATCAC
GTAAACTTTTTTAATTCTTATTTGGTGGAAGCCTACCGTTTGTCCCATTGGTCATTTCTT
GACCTTCTAATCAAACCATTTTAATCTATTCTCCTCCTGGAAATTTTGTTGCAGGTCTCG
AAATTTCCATTTTCAACTAAATCTTTCTTTTAGCTATTTCCTTTATCATTCTTGCTGTCCTT
TTCCCTTGCTTACTCAAGTCGCTTTGTAGATAACTGACTTAAGTATAGGTTTCTATAACA
TTATAAATAATGTTGTGTGATCAGCTTCATTTTTGTCTAATTTCTTTTTCAATTTACCGTTT
ACAGGTGAAGCTGCGTCTGCACACTCACCGTCAAAATGTGGAATCAGTTCTCTCAATG
TACAAAGATACTATATTCCAGGTATTCTTTTTGCCACAGATGTGGTGCAGTTGCTTCACT
CGGGAATGCAGATTGAAACACTCATCAGTCACCATCAAAGTGTGGAATCATTCCTTATA
TACTTGATTCATAGTTTATTGGGTTGTTTGATGGTTTACAAGGTCTTGGTTAACAGGAAT
ATAGTCCATAAAAGACAATGTGGAAGGGACTCGGTGGAAATGGTTCGATGAGAAAGTA
GTTTGGTTTTCTATTTGGCAAAAGAACAATGATATGGTTATAAAATTTCGTTGTTTGACA
TGTAGGTTACAAAATGATTACTTTAAATTATCTTCAGTTCATCCAGGTCATCCTTCCTCAT
AACAGTTGTAAAACAAAGGAAAGCCAAAGCAAAAGGGCTATACATCCTCTTTTATCTT
AAAATTTACTCAATAAGCTAAAGGAAGAGTAAATCCTTTTGAGTATTACATTCATACTAA
TCAATCTGGGGGGAAATATACTTAATCAAGGACTCCCCTTCCCTCTTCAATGAACAAGG
ATACTGTATGTTCTTTCTTTCCATACGCGTGCATGATCGTGTTGTTATCATTTCCTGGTGT

TAAAACTTCCATCTTCTCTGAGCTATTTAATTTCAGGTGGACGGGAGCGTTTCCAAAG
AGGAAGTATTTGCTCAAATTGATGGTGCATTAACTCAACTTCTTGAAGCAAAGGAGTG
AAGATTTGGAACCGTAATGTCATAGAGATCAGGAAAAATCTGCAGAATGTACCATGTAA
TACTCAGGAGGATCATCGAAGACAAAAGAGTAACATTGTTGGTTGCTATCCCTCTTATA
CAAATGTATGAAATGTTTATGTATATCGTATTTGCTTCCAACTATCTATGTGGATATTCATT
GAATCAGATTCAAAGTTTACTACAAAACTTGTTTTGTTGTA

>SlADK9

AACCAAATTAAAAAAAGTTCCTTTTTTTTTTCTATTGGAATCAAAAGAGACCAAATCCA
CAAAAGTGACACTTGTAACATCAGAAACTGAGAATTATATTCCCATTCTCTCTGGTCTTT
ATTTATTATCATAAATAAATTATAGTATATATAAGTTTTGTTCTACTTCTGCAACTTTCTAA
ACAAATTTGCAATATTGTGCAAGTCAGGTATTTTCCTTTCTCACTCAAACATTTTTTTAA
AAAATTTTTTTGTATACAAAGTTGTGAACTTTTTTGTTTTGTAAAGGTATAGTTCTTGGA
AATTTTGATGGGAGTTGAAAAAAGGGGAAATTTTGTTGTATGATGATTGAGCAATTGAT
TTGATCTGTATTCTGTATGATGTTTTTAGCTTTGTAAGAATTGTTGGTGAATTCAGGTAG
AGTTGGAGTTAGAATTTTGAGTTTATGAATACGGGATTATAGAAAGAAAAAAATAAGGC
AATTCAATACTTTGTTATATCGTGCTTTTCGGAAGTCAAGTTTGATTAATTTTCAAAATTA
AATTAGATCACAATTATTTGATATATTAAACAAAATAATTAGATATTCTAAAACTATATGTA
AAGTACTATAGATTGCGATTTGAAGTGGATGTTAATTTGATGTTTTTATAGTTTGACTTTA
AAAATAGAAATTATGACAAACAATAACGGACGGACGGAGTTTTGTTCTTGCTATTTTAT
TTTCCAATTATTTCTTCAATTAAGAAAACTAAAGAGAATAAATAAGATTCTCTTAGCTCA
TTCGGGAGGAATACGAAGGAAGAGAATGATTAGAATTGGTTTTTGACTATTTTATCCGT
CCTTTATTTTTAAAACATTAATAGAAGTTTAAGACAAATAACAATTTCCCCATTTTGTTC
TCAATAGTAATTATTCTTTAAAACTACAAACACCTTATTAGTCAAAGTTCTACAACTCA
AATGAGGTGTTTGTAGTTACTCCTTTCGTTCGGTATTGTTTGTCATGGTTTCTATTTTTA
GAGTCAAACTATAAAAATTTTGACTAACATTTTAAGAAGCATTTTTTCATCATATTAATA
TGCAAAAAATTGTAATTTATAGTACTTTTCATATAGTTTAGAATATATCTTTTTGTTTAAA
ATATCGAATTAATGTGGTCTAATTTACCTTTGAAAATTAGTGATATTGACTTTCGATAAGC
GCAACATGACAAACATTTTCGGACGGAGGGAGTATTGAGAATAAAATGAAGGAAATA
ATAAACTTCTAAAATGATAAATAATTTGAGACAGAATGTTTTTAGCAATCATAACAGATA
ATTTGAGACAGAGGGAAATGTCAAGGTTTGGGCTAAAGTTAATTGGTTATAGTACTGTT
ATTTTAAACTTTTCTAGCAGGAAGTTTCATTATTGTTTTTCTATTTGTGAACAAGGTAGC
TGCAGGAACTAAACATATTCTTTGTGCAGGCAATCCAAGATGGATTTACACAAGGTGA
GAAAGAATCTTTTGGTGTTTCTGATTTCGATTCTATTATAGTTATCCGTATCGTCTTAATTT
ATTTTTTCAAGTTTGTTTTCATTGTCCTTCAAGTAATTGGTTAATATGCTTTGTGAAGGTT
GAGATCAAGAAGTGTACTGTTTATCATCGTAATTGATTTGGTACAACACAGATTAATGAA
TACTCTTTTGTTTTTAGATTAGAGATTTGAAAAAAGAAAAATTATTTTGGTTAATAAAGA
GAATGTTTCTTAAACTTGTTTCACCACTGAAGTCAACAACATTTGAGGCTTGTGTCCAA
ATATACAAATCGTACTTGTAATCTTACAAACAAAGATTCTAGTGATAATTCCTCATATGCT
ATGTCACTCTCACTGCTGAGTGCACATGAACTCTTCATGGTAAGGCATGTCCTGGTTCG
TAGTCTCGTTTAACACGAACTTTCCCAGCTCAGATATTCAAAGTTTGTTGAGTAATAGT
CCTGGGTTTGTATAACATAGACATCATTCTATTGTTAATGTGATAGTCATTTATGAGTCTT
GCCAATTAAGCTAATATTGTCTCGAGCTGGTAGTTTTCTTATTTTTTGAATTAAACCATTT
AATACTCTTTTGATCATTTGATCGGTATCTCGTTTTTGCAGGAAGGTGATACAGGCTCCG
CAAAGCAGAAGAAGGTCAAGATTGTTTTTGTTATAGGTTGGGACTTCCGTAATTCTTTT

ACTTGCACTACCTGTTGTAATTACATCTACTGCAGTTATCTCTTTAGATGAAATTAAAAC
TGCATCTTGTGTTCCTGTTTTATCATGAAAAATGCATCTAGTTTTAACTGGTGTTTATACA
TAATATCTTATTTGGTCACGGTGGAAGGTGGTAAAAATTGGAAGTGACATACCATTTCT
GCGGCTTCCATGCCTTGCATATCCGACCACCAGCGTAAAGGAAGGAAAAGCCACATTT
TGCTGTGTTTTCTCTTTACCTTTTTGTTCTTATAGCTTTCCTCTGTTTAATTTTAAAATGAT
AGCTGAATATAAAAGTTGAGTGTGTTTATGGTTTCTGTAAACACCATTGACTTATGATCA
GTATAGTCATCTGGATTTCACATCCAGTTTCGCCCAAAATGAAACAAGAAACATTGTTC
ATTGATGGTTATCAAATGGTCTTAGTTTAGATCGCGAGAGGGTAGAATTTACATGTGGTA
ATAGTGGAGGTTCATTCTAGTCTTTAGTTACAGTTTTATAAAAGTCAAATATAAGGCAGG
TTTGTGTTATTTCAAACTTCGGGGGACTGAACTTGAGGGCTACAGATATGCATTCTGCT
ATAAAAACTCATAATGAATAAAGAGGCAGGACTAGTGTGGTCTTGTGTCTCAGAATTTT
AGTTGTATGCATGATAGACACGTTTAAGCGTGTTAAAGGTATATTCAAGCACTTGATCA
GCTATAGGAAATTACAACGATGAACAGTGGTCTATTTAGACAATGTCTTTTAACAGTGTA
TAGGTTGAACAAACTAGAAACTAAGTTCTTGAAGAACTTCTTAATAGATGAAGTCTCGT
TCCAAACCTTCTCTTGAACTTGCTTCAGTGTGCTTTACACCTTTGCATGATCAACCTTGT
AAGTCGTTAATACAATGCTAACTAATACATTATACGTACATTCCTAAGCATAAGGAGTAC
TTAATAAGATCGCAAGGGACAGACGTCATAGAAGTGTTATGGATTCTCTTGCTTGTGGC
TTATATCATGTGATATTTCTCTTTATCTCTATTTTTTTAGAAACATAACTTCCTCCTTTCCT
TATTTTCACATTGGGATGCTTTATGTTACAGGTGGTCCGGGGAGTGGTAAAGGAACACA
ATGCAAAAGAATAGCACAACAATTTGGATACACTCATCTTAGCGTTGGCGAGATTCTAC
GTCAAGAAACCAGTTCTGGTTCTGAAACTGGGTAATGTTACGTTATTCAGTAACCGTTT
TTAATTCTCGATAAATTCGTAGTTGTTACCCTAGTATGTTAACTTATATGCTCCTAGAGTA
TATACAATGCAATTAGTTTCATCAAAGATATCCTCTATGCCGTTACTTGGATTATATCCAC
TTTGTAGGCTTACAGTTCTGTTAAGTAAGAATGTTATGTCACAACGCTGTCTTTATTTCT
TTCGTTCGCTTTATTTCAGCCATATGGTTCAGAAAATTATGAAGGAGGGAAAGCTTGTT
CCGTCGGATGTAACAGTGAGGCTTCTTCAACAAGCCATGCAGGGAATTGATAATGACA
AATTCCTCATCGACGGCTTCCCCCGGGATGAAGAGAATGTTAAAGCATTTGAGGATCTT
GTAAGTTTAATTTTTTGTTCAACTGTTTCTAGTGTAAGTCAGTAGCACCCTGATTTTGCT
TGTATAATTTGAAATAACTATCGTTTCGGATTTCTTTGTAGACAAAAATGGAGCCTGAGT
TTGTCCTTTATTTAGATTGTCCACAAGACGAAATGGAGAAGCGCTTGCTATCAAGAAAT
GAGGTATTTTTATCCTTTGTTGTTCAGGCTCTCCAAAATGCTGCCCCACCCGTGTCGGA
CCCTCCAAAAATGCACTACTTTTGGAGGATCCAACATGCACCTGACAGAATTTTTGAA
GAGTCGAGGAACATAGTATTAGGGTACATCGATTTAACACTTACATCAAGGACCTTTTT
TTTAACTTAATTCAGAGTAACTATGCAGGGAAGAGAGGATGATAACATCGAGACAATAA
GGAAGCGATTGAAAGTTTTCGTGGAGTCAACTCTCCCTACAATTGAATACTATGAATCA
AAGGGGAAAATTAGGAAGGTAAAACACTACTCTATAATCTTCTTCTATTGTTGAGTAGG
GTGGTGGACGTAGATATTGAATCGGTATAACATGTAACGATTTTAGTCGGAGGTGAGTG
CAACATTTTAGCATCGAGTTCATCTGAACCCAATACTTTCAACTCAATGCAGAGCATAA
ATTTATATGTTTAAAATTGTATATTTGTCACACATAGTACTTGTGAACTTATAAATCTTAA
AAATACAATAGTTCAACGTTAAGAATTGAATTCGTAAAGTTTAAATCCAGGACACATCC
ACCTGTTGTTTTAGTAATAACCTCTTCCTTGTGTCTGAACATATCAGGTTGATGCTGGAA
AATCTATTGATGAGGTTTTTGAATCCATCAAAGTTATTTTCTCACCAGGAAAAGATAAC
AAGATGCCACCAAGTAAACACAAGTGCAAATGCTTGATACTTTGAATGGATGATTGTCT
TCAAAGCTTGGTAATTCACAAATATTTTTACTAATGCGCGAGATTTACCTTATAGATACTA

TCCGTGTGTTTGAACTTGTTGCAATAAGTCATGTCGTGGCTATGAATCCATTATTATTGA
CGGCTGGAGGCCAATCGTCTTTCTGGGGATTTGATTGTGTGGAAAACTATATCGCAAGT
GTATATAAGCTAAACTATGTATCGTATGATATCTACATGTTTCTTGTTAATGAATGAAATG
CAAAGGCATAGTGTGTTGTGAATTTAAGTAGTAATATATGTGTTACAATCACTTGTGATC
TCTGTTGGAAATTTCAAATGACATA

>SlADK10

AGGAAATCGCCACCCTTTTTCTCCCCTTTTTCACTTTTTCTCTTCACACTTCTCAAGAA
AAATAACTCCGGGAGAATTTTCCGGCAATGGCGGCCATGATCCGCCTGTTCAGATCTTC
ATCATCTTCATCATCTCTTATCAGTAGATCTTTATCTACAGCAGCTGCATCTGAGACAGT
GAAATCCCGATCTTACCCTCATTCTACAAGTGTTGAACCCAAGGCTAAATCTGTTCAAT
GGGTCTTTTTGGGTTGTCCTGGTGTCGGAAAAGGTACATATGCTAGCCGTCTCTCAACC
CTTTTAGGCGTTCCTCATATTGCTACTGGAGATCTTGTTCGTGATGAGTTGAAATCTTCA
GGTCCTTTATCGAAACAAGTATGTTGTGCTGCTTGTCCCTTTTTTTCTTCCAATAAATTT
GGGTTTTATCTGTAAAGTCAAATACTTGTTGCTTAGAGTGCATTCATAAATTTTGAGATT
GATTTGTTGGTGAGTTAATCGGAAATGGTTTCTCTATCCCACAGAAGTAGTGATAGTAG
TGGTAGGGTGTGCGTGTATCCTACCCTCTCCGGAGTAGTTGTTGTTGATTTCACTTAGA
GTGATCGAAAATAGTACCCCATAAAGGTAGAATTAGATGTGTATATCTTACCTCCCCAAA
CCTCGCTTGTGTTATTACACTGGGTATGTTGTTGTTGATATCTTCTAGAGTCGCCTATGTG
TTGTGTAGAGTAAAGCGGATGTTGTCCGAGGCACAGATGGAGAACGTAAGATTGGGAA
AATGATTCTTTATGATAGGGAATGAAGGGATGGTAGACTAGATTGGATCTAATTTGGCA
GAGAAGAGACCGCGAAAATTTGATCCATTCAGGTAATGTCTGGAAATAAGTTTGGTGG
AAGCTTAATAATTGAAGGTCGGCCTTATCTAGAGTTTTACTTACTCGTATATCAAGTATG
CTATGTTGTTGTCAGTGGACGAGACTATTCCTATACTTTTGGGGAGTCTTTTAGTCATGA
TTTAGATTTATATACCTGTAAAAAATATAGACGTTCTTGAAGTTATTATTTATGTTTTAATT
TTTGTGTGTTAGCCTTAGATAATCTAAAATGGAGCTATATGGTAATAGAGGATTCATATG
GCCGACCCTAGCTTGAGTTCGGGATTGAGGCATAGTTGTTCTTATTATATTATCTCCAGT
TTATGGTGTTACTTCATGAAATTTGTTCTTCTCCTGACCGGCTTCATCCTCTTTCAGCTT
GCAGAGATTGTCAACCAAGGAAAATTGGTTTCAGATGAGATTATACTGAATCTACTATC
CAAAAGGCTTGAGAGTGGGGAAGCTAAGGGTGAAGCTGGATTCATACTTGATGGTTTC
CCTAGAACTGTGAGACAAGCAGTGAGTATACATTTAATTTTCCTGCATAAAATTTATCTA
TGCTCTGGTTATCTCCATAAACCTTATATATTGAGATTGAAATGTTATGCTCTGCTTATCT
GTGAGAAAATAACTAGCCTATGACGGTGAGTATGAATTAACCATTCCCGTCTAACCAAA
AAGAAGAAAAGTAAGAAGTTAGAGGCTAAATGCTGCTCAAGTTAGGTTCAATGTTAAA
TGCATTAGCCTGAATATTGGAAGTCAAGAATGATGCTTGGAATAGTGTATTGTTGTATTA
GTTACTTAAAACCTTCATAAAATAGGATTTTATTACCACTGTTCATTAAAGTAGCTTCTT
GAATGCCTGGTAATTTCTGCCCTGTTCACGACTTCACCCTACTTCTTTGTCTAATTTATAT
GACTTTTGGTAAAAAGAATTGTGACAAGACTTCTGATTTAGTCTTGTCAGCAGAGGTA
TAGGAACTGGGAAAGAAAGTGAAGATCCTTTGCAACAGTTCGCTACCTTTTTGTCTAT
GAAAAGGTACCCCCCACCTTATGTGGCTAGCGGGGTTTGAAAAGAGGATGGAAGTGAT
ATCAAAACTCTATTTCGTATTCCAGTCGCTTCTCCAAGTACATTTGTGTAATCAAAATTT
ATCTCAGAGGTGTAAAACAAAGTTTTGTGCTAGTTATATAACATTCGTGTCTATTTCTGC
ATCAGGAAATATTGACCGAGGTGACAGACATAGATTTGGTGGTTAATCTCAAGCTTCCA
GAGCGGGTATTGGTTGAGAAATGCCTTGGCCGAAGAATCTGCAGTGAATGTGGAAAGA
ATTTCAACGTGGCATCTATAGATGTCGCTGGTGAAAATGGGGCTCCTAGAATCAGCATG

GCTCCCCTTAATCCTCCCTCTCAGTGTATATCAAAGTTAATCACTCGAGCAGATGATACA
GAAGCCATTGTGAAGGAAAGGCTCAGTATATACTGGGATAAGGTACTGTTCTGATATTT
TAACCACAATATTTTCAAGTTCCGCTATTACTCTAATAAAGCATTTTTCCTCAACACGAG
TAACTACTATCAGAGCAACACTCACAAGTTAACGCCTTAACAGTCATTCAGCATCTATG
CTATCTGATTCTCGCTCTTATTTATGATTGCAGAGTCAGCCTGTTGAGGACTTCTACCGT
AGCCAAGGTAAGTTACTGGAATTTGATTTACCGGGAGGCATCCCCGAATCATGGCCTAA
GTTGCTGGAAGTTCTCAACCTTGATGAACAAGAACATAAACTGTCTGCTGCAGCTTAG
TTTCTCTCCGCTGCTTTGCTTGTGTGCTACGCGTTAAACAATTTTGCAGAAAAAAGAAC
ATATTGATTCGTTTCCCTTCTATTTTCCGCTTAAATTTAAGTCAAAATAACAAACCTTTTG
AGTGTACTTTGAAGCCGTAACAATACGGTGGCATCCATTGTTGAATTTCGAATTCATATG
AAGAGGTCTGTTGTACCATAGTTTCAACTAAAGAGTTATAAAGATGATTATTTTTTTACA
GTTGTGTAATAAGCTTATAGTAATTGATAGCATTCTGATTTAATACTTAGGTAAATGACAT
AATTTGATATATGCAACTGATATGGTTTTGTTTGGTGCATTA

>SlADK11

ATGGCGATGATAGCTTCAGTCACTATGAATTTTCCTCACATTTCTACTCATAATATTTCT
TCAAATCAAACGTTTTCTCCAATCTGTACTAATAATCCTTCTAATTTCTCATCATCGTCG
TCTTCATCAATACCAATTTCTTCGAATTCTATCCGTTTATCATCTTCAATTGCTTACTCG
GAACAACTAATCGCGTCTCACAATGTCAATCGACGTACTAAGAATCGCAAAATCAAGG
TTAGCATGTATGACTAGTAACGAATTTAAGTTATATACACTATCCGTGTAAAAAGAGTTT
TACATGAACAGGTCAGGTCGGGTCGGGTCATATTTGTTTTCAAGTTTACGAATCTCACA
TTTACACTCACAATGTATAATTTTGTTTATCGAATTAAATTATGGAAGTATTTGTTTCTTC
AGGTGATATCTGCAAGAAGTGAGCCTTTAAAGGTGATGATATCAGGTGCCCCTGCATCT
GGTAAAGGGACTCAATGTGAATTGATTGTACAAAAGGTTGAATTCTCGATTCCTCTTGT
TTTCTTCTGCTAGGTGATGATTAGAATTATCTTTTGACTGGAAGGGGGTAGCAATTTATT
TTGCCCGACCAAAACTAGTTAACGATTGGTACATAGTTGATTCAATGTTTACGACGTTG
TGTTAAATAGGATTGACCATGACCTATTTTTGAATATGCTAAAGCATTGAAGTAGTTCAA
TTCTTCTTGTTATGTAGATCATTTTATGCACGTGTTGCATAACGTGCCAAGTGTTCGTTAT
TTGTTTTTCGTGAACAGAAAAGGATGTAGTGGGATGGATGAAATCCCTCTACCCTTTAC
AAGAGGTATCGGGTTTGAGCAACGGGAGTGGAGAAATCTCTGGTAGTGAGCGCTTCCT
CCTTCAATATTGGAGTTACGCAACACAAATTTTGTTTAGTCAGACCAATGGGTTGATTG
AACAACAAAAGTTTGTCTTTTGTGGTGTGACGATTCATGTTGTACTTAATGAAATTTGT
GAATGGGAATGTTGGTTGGTGTAAAATCTCCTCTTGAATGATATTTCATCATGGTTTGTT
TCAGTTTGGATTGGTGCACATATCAACTGGTGATCTCCTACGAGCTGAATTATCGGCTG
GTACAGATATTGGAAATAAAGCTAAGGAGTATATGAACGCTGGTCGCCTGGTTCCTGAT
GAGATTGTAACAGCTGTATGAGATTTTTCAAGTGTCTTGTTAATGAAAAAAATTAACTT
TCGCTTTTTATGATTTTCGAAGTCGACTTCTGTTACAGATGGTGACAACACGATTATCAA
AGGAAGATGCAAAAGAAAAAGGGTGGCTTCTGGATGGGTATCCACGAACGTTGGCTC
AAGCAGAAAGTCTGGAAAGGTTGAATATCAGACCAGATATCTACATTGTGCTTGATGTA
CGTCCACTCTACCATTACATATTTGCATTTTCTTTCCCGTCACAAATAAGTGATCTTTTGA
AAGTTTACTAAAGATAGGTTAGATGTTTTTGTCATTGTTCCAAATTTTTGATGCTGTATG
CGATATGATGTTGCAATGCTGGTAGCGTAGTGCACTTTGAAATGTTTTAGGTTTGATCAT
AACAGAAATTGAAATATCTTCAGAGTTGAGGATCACTTTAAGTAGGTTGAGTCATTCAT
ACATGTCCAGTGGATAAAGACATTGTAGAAAACATGAAAGCTTGCCTCTAAATGTTGTT
TGGACTCTCCACAAATGTTGCCACACCCGTGGTGGATCGTCCAAAAATACACTACTTTT

GGAGGATCCAACACGCACCAATCGACAATTTGAAAAGTTTGAGCAATATAACCCTCAG
CCATATTATGTTGAGTGATACAGTCAATGACTTCCTTGAACACTACTAGATGAAAGCGC
ATGGATTGAGAAAAAATAAGTGAAGTCTTCTTGTCATAGCACACATTCGTGAATTGATA
TGGTGTCCGATGTTCTTCTTGTCATAGCACACATTCATGAATATACCAAAAAGAGGTATA
ATAGAGCAAGAATGAGATTAAGAATTTGCTTTTAAGCTAAAATTATCTTTTTAAGCATGT
TTGGAGTGTTTGTATAAGTTAAAAAGTGCTTATAAAGCACTTAGTTTTAAGATAAATTGT
TAATATAAGCCAAAACCATAAGCTAGAGTCTAACTTATGGCTTTTGGCTTATGAGTCAA
AAGTGAAGCAAATCAAAGAGGCTAGTCTTCTTCTTCTTCCATTTGATTTGTGTTCGATC
TTTTACACAATAAGTCCTTCTCTTCCACAGGTTCCTGATGCGATTCTTATTGACAGATGT
GTTGGTAGAAGGCTAGATCCTCTCACTGGTAAAATATACCATGTTACTAATTTCCCTCCA
GAGACCGAGGACATCAAAGCAAGGCTCATAACTCGTCCTGATGACACAGAGGAAAAG
GTACTTTTAACCCCCATATTGTTTGCCTTGCTTTAGTTTAATAGACGTATACTTGCTTTTA
AGACTGGGTTATACAAATCATTTTTTTGCTTCTGATATTCAAGACACCATAATATAGACT
AATCCTACGTAGTGCCCATCTTCGATATATCTAGAAAAGCTGAAGCATCTAATATATCCA
CAAGTCTACAGTTTAACCAGGTGTGCTAAATGTGTTTCAGGTGAAATCACGTCTGCAAA
TATACAAGCAAAATGCTGAAGCAATATTACCAGTGTACTCAGATATAATGAATAAGGTA
GATTTGGCATTTACTCTTATTATAGTATAATTTTGAAATTCTGTATGTTTTCTAGTGATTTG
TACTTATGAGGTGTGGCATACAGATTGATGGGAACCGCGGCAAAGATTCAGTATTTGCA
GAAATTGATTCCTTATTGTCACGTGTGCAGAAAGAGGAACAAGATGCAAGAAAATCAG
GTGTGAACCTTTTTTTTATAGAAGTATCTCTCTTGAGGTTCTTTGTATTCTATGTAGAGCT
ATGTTTTAACCAAATTGAAATTCCTGCTGATCTCGGCAGCATATAATCCTATAAGAGATG
TAAAGATTGCGTAACACATGATTAACCATATGTCTCAATTATCAATCCCCCGCCTTTTGT
TCTGCACCCACCTCAATAATTAAACTATATCTCTATTCTTTTTTCTCATATTTGTCATCTTG
TTTCTCTTCATGCTGTGAAGCCTGTTTGACTTGAAAAACATTGGAGTAAACTTAATTAA
ATTAAGATAGGCTTGTCTGATGTTTGCAGTTCAAAGCAAATGGGAACTTAATTAACATG
GTATATTTTGTGAAGCTAATATGGATGAAATCAAGATGATAGAGCTTTTGCAGCTTTTCA
CTTACACTTTATAGTATTCGGAAATTGTTTAAAGCATTTTCTGAGTGTGATCATAGAAGA
AACTAAAATACCCATATATAAAACTTGTATAGACTCCAATGTTTCCTTTTCTCTTTTCTTT
TTTCTTCTTTTACAATATGCTAAATATACTTTTTAGAGGAAATGTTGCACTCTTGAATATT
TGAACAATCATTTATAGCTTTAGGTAATTACATCCTGAAGCAGTGAAATAAAATTTCCTT
CAAGCTCATTTCCTTTTGTTTCCCCTAAAAACCAAATACCGAAATAGATGTTCCATTTAA
CAATAAAAAAGATTTTTTGCTGGACACCTTTTTGAATTTCTTGTATCAACATGCTGACA
AACTAAATTAGCAGTCATCACTTAGCTCAATGACACTTTGGACCCTGAAACTCTACCAT
ATTTTGTTTGCAGAAGAATCAGCAATTTCCAGTACTCGAGCTGATATGGCATCTTTGAG
CAAGGTAATCATCTGCTAAATCACGAACACAACTTCCCTTCTTTTTTTGAAGGAAAGTT
GACCGTTTTCCTTATATCGTTATAGGATTGGAGAGGAATACCTACTAGACTGAATAATAT
TCCTCACTCAAGAGAAATTAGGGAATATTTCTACACTGATGTGCTTCAGGCTACTCAAA
GAGCTGTCAATGATGGGAAAACTCGTTTAAAGGTATGACTCTGCCCTGATTCGAAGGT
AACTCAAATATTCAAAAGTAATTCAGATAAGTTTAACATCGAAGTAGTTTGCGTTGGTA
GAACTGTTTTACAAGATAATTTTGATTGATTATCATTTTAATTAAGACACCCCCTTCTATC
AATCGAAGGGGAGTCTTGTAGCAATGGTGAAGTTGTCTTTGTGTGACCTATAGGTCAC
GGGCTCAAGCCATGGAAGCGGCCACTAATGCTTGCATTAGGATAGGATGCCTAAATCAC
AGTGCTTGGGGTGCGGCCCTTCCCCGGACCCTGCTAAAGCTGGATGCTTTGTGCAGTG
TGCTGTCTTTTTTATCCCTTCTTTCAACCATTTTATTAAAGTGCCACGTTTTCCAACCACT

TTGACTGAGAAGTCGCCTTTCTTAGTGGAAGTAGATACAAGATATTATATTCATATATATG
TGGTATACAGTTTTCTGAATATACCATGATATGCCCCCTTGAGTGAGTTGACGAGTTTCT
GAGAAGAAAGCCAGTATGTTTCTTCGTCAATCAATGAAGAAATATAACTTGTTATGATA
CTTTAAATCTGTTGACACATGAGTTTCACTAGATTATTTTACCCTTGAATTTCATATTCAA
TGGAATCTGGAGACATCTCTTATCTTGTTTCTAAAAATTTATGCTATTGTGAGAACGAAC
GACTAAGTATTTCAAAAGACTTACGGATAACATGCGATTACTCAGTGGAAATAGTTCCA
ATGAATAACTCTTCTTATTTGAAGGAAATATGCCATATTCTGGTGTCCACATAACTCGAA
ACATGGAAAGGTCACCTTTTCTTAATTTTGAGAAAAGAGTAAGAGAGAACTACCAGCT
CATGTTCCCACAAACTAGTTTTATAGATAATAAAAGCATGAAAATAGCTAGCTCAAAAG
ATGAATCTTGTCCCATTCTATTTAATAATATCCTGTAAACTTATAGTATATGCTCTTCGATA
TTCTATCTAATCTCATATGTTAAGTGTGTTTCGACAACTGTGCTGGTGTCAGAATATGGT
GCATAATTTGTTAGGCTTTTTGGGAATAAAATAAGGAAAAGGAGCCCTTGACCCAGTG
GCTGAGCTAGAATTTTATCTAAGGTGTGTCAAAATATTAAGAATCAAAATCGTGCAGAA
GCCAAGGAGTGACAATATGTAATTTATATACATAAAAAATATTTTTTATTCTAGCAACATA
ATGTAATTTTCCGACGAAGGGGTGTCGGCCGACACCCCTTAACTATATGTGGCTCCGCC
ACTGCCTTGACCCACAGGAGGATTTATTCAATAACATAGTTTGGCTCCTATCATATGACA
CCTTAGGGCTTTCTATTTGATTGTATCTTAAAAATGTGGTTTAATATTGAAAGATCAAATT
GCTATGAAATGAGAAAAACTAAGCTGTTCATTTATTTATGCCAGAGAATTAGTTTTATTC
TTTATTAAACCTATAGAAGGAATCCAGGCCTTCCCCTATCTACATTTATCGATTTATCCAA
GTTATATTAATACCTTTATCTTGATTTAACAGATAGAAATCAATATCCCGGAGCTGAATCC
TTCAATGGTGAGAAAACATTTTCTGATCATATGCCCATAGTTTATTTAGGTTTGACAGTA
TGTTAAATCCACAGGATGTTTATCGAATAGGTACTCTAATGGAACTTATCCGTGTACTTG
CTCTTTCATTTGCTGACGATGGAAAGCGTGTCAAGGTATGTGTCACTGATGGTTATTCTT
TTCCACTCTTAGTTAATCTAGTATCTTTGAAATAAGGCAAATTATACATATTCATTTCTCC
AGATCTCATTTGTAGGTTTGTGTCCAAGGGTCTATGGGGGAAGGTGCACTTGCAGGGA
TGCCTTTGCAGCTTGCAGGAAGTCGAAAGATATTAGAGTACATGGATTGGGGTGATTAT
GGCGCGTTGGGAAACTTTATCAATATTGGTTCTATAGGTATTGTAGAGATTAAAATTGCA
CTGATGACTTTCACTTCCTATCCAATATCATGTTTTTAGAAGCATACTGCAACTATAAATG
GTCTTCTAAGACATAATATACAAATCTATCCGTGTGCGTTCGTGCACAACATTTCTCTTT
TTTATGAGATAGGGGCAGAGCCACAGTAGAAGTTTTGTGTTCAATAGAACAACTTTGG
CTCAAACGCTGTAGTTGTATTAAGAAATTCAATGCATCCCGAACCCAGTAAGCAATAAG
AGGTGTTCTAAACCTAAAATCGTAGATCCGCCTCTGTTGTGAGGGGTTTATTTGAGAAT
GTAGTTCTAATATAGCTGATAGTAGAAACTTCATAGCTTTATAAATGTTTCTGTTGTGTGG
TGACTATTTGTGTATGTTGTGCAAGTATACACATGCTAACATGAATGCTTAACCCTCAAT
TTTTTCTGAGGTTAGCATTGCGATAAAACACAATATAATCACACTCTTGAATTCGCTTCT
TTCACGATTGCTGGTCAACTCCATGTTTACTACAAATTTTAATGTGTTTTTCAGGTGGCA
AGGAGGTTGAAAAACAAGATGACGTGTTCATTCTAGTGGCTCCCCAGAATGCTGTCGG
AAATTGCATCATAGATGTGATTCACTCTGAACCATTTTCTGTATTTTCCTCATACTGCTCT
TATTCAGTTTATTAAATTTTTTCTCTCCTGTTGAATAATGCAGGATATGAGAGCTATGACT
GATGCAGCCGGTAACCGACCGATTATTCTAGTCAACCCCAAACTAAAGGTTTGGATCTT
AATACCTCGTTAATGAATGTGACTTCCTTTTTAACTAAAAAACAGCTCATGGCTTACCAT
GAATCTCAATCGCAGCAGCAAAAGTTGTTTGAATGATAAATTAGTCTCACGTCCCTTCC
AATACTTAATATCACCTACCAAAGTGAAGCTTGTGGTTGATGCATATACAACAATGCTCA
TTTTAAATTTCTTTTTGTAGCTTATCGTAAAATACATTTTGATTCTGGCGGTGTCCAAGA

ACTTAAATGCAACGTCTCATACCCAAGTCCATCTGTCCATGTAACCTAGAGAGTACTGT
TTGACATGTAGATATGCAAATGAGACGAATTCATTAATCTACTGAAAGTGTTTATTCTAG
AAATTTTTTAAAATTTGAAAACTGTGCTTAAGCCTTCTAATCTGGATAAGGGTGTTTTAC
CATCTATGTTACTCTGACTTTTCATAAATGTCAGCGGGTGCATGTCGAATTCTCCAAAAG
TAGTGTATTTTTGGAGTATCCGTGTGAGTGCGGCATCGAAAGTAGAGAGTCACACAAC
TTACTTTACCATTATACACTAAAATATTGATTACGAACTCAAACCATCCATGTATTATCCA
TTTATTCGTCTTGCATTTATACTGATTTTTCTCTCAGAGAAAACAATATTTTTTAGTTGCA
CTAGAAAACCCGTTAAATAAATTTTGCTTACACTTCCGGTGTTCATAGCTTGACTCAAC
TTCTCCTCGATGATGAAATGATCATAAATGGCTTGACTTTGATCTATCACATACTAATTGT
GCAGGATTTACCTGCTTCAAGTGGTATCATGCAAGTAAGTATCGTAACCATTGAACCAT
AATGGCCAACAGTAAAGGGAACTTCTTCCTACGTTCTGATTGATTTTCTCTCTGTCTTC
AAGACAATGGGTAGAGATAAAAGATTGGAATATGCTGCATTGTTTGAGATATGCTATCA
ATTCCGGCTACTCTACTATGCAGGAACACAATATCCTATTATGGGGGCACTCAGGTTAGA
TTTGTTTCTATGCTATTTGATTGGTTTGTCATAACATGATCATGAAGAAGAAGAGCAACT
TAAAATGGCATGAAAATGCTTTCGTCTTTTTTCGGTTTGGTGAGATAAAATGTGGATTC
ACGGATCACTCCTTTGTCATGACTGATGTCACTTTTTTTCCCTGACTTTTCGAGGTCATG
AAATAAGAAATTATGTTATTTTGGTATTCTCAGTGTGTTAATCCATAGAATTTTTAGAATT
TGTTCGATCATCCCCAAACTTAGCGGCTTCACCTGAAACCTCCTATAGTGACCAATACC
CATGGCTTCGTCATGATTGACATCACTTTTTTGGCGTTTCAGCATCATAAAGAAGGAATT
CAGGATTGTTTTGGTGTTGTTGTGTGTGTTTGCCCATGGATCTTTTGGAGTTTGTTCGAC
TGAATCCGTTCTTCCCCAAACTCAGTGGGTCCATCCAAAACCGCCTGGTGACCAATACT
CTCATTGTTTCGCCATGACTAAATTCCATCTTTTTTGGCATTTCGGCATCATGACTCATAA
AGATGAGATTTAGGGATTTTCTGGAGTTTTTGATGTGTGTTAGTCCATAGATTCTCCGGA
ATTTGTTCAATCGACTCCATTTCTTTCCTCAAACTTGGTGGTTCCATCGAAAACCACCT
GGTGACCAATACTCTCACTGTTTTGCCATGACCAATGCCTCCTTTTTTAGCATTTCGTCG
TTAATGAAAGTAGGAATTCAAGATTTTTTGGAGTTTTTGATGTGTGGTAGTCATAGATTT
TCCGTAACTTGTTCGATTGACTGTCTTCTGCCCAAACTTAATGGGTCCATTCAAAACCA
TCGGATGACCAACAATCATTGTTTCACAATGAGCAATCTCATGCCAGCACAGGATGTCG
TACCCGTATCCTTATGATAGATTCTTGTTGTGATGGCCTCTGCAAAAAGGAATGGCTGAA
TATATCCATTGTCAACATTCACTCTCTTGCTAATTATCTCATGTTACAGGATGTCGTACCC
GTATCCTTATGAGTTATACAAGAGAGTCGATGAATCACCGGGGAAGGAGAAATACATAT
CCTTGGCAACATTTGCAAAGAGGCCAAGTATTGATGAAATGAACGATGCCTTCGAAGG
AAAATCAAGGTACACTACAAATCATAAACCTGAACTCTTTATAAACTCCTACACCTGCT
AAATCTGTTCATTATACCTCTACATCACTGCACTAAAACAATACTTAACGATTATAACTC
TGCAGAAATCAAGAGAAAAAAGCAGAGGGATTTTGGTATGTGATTAACTATCCTCCGT
TTTATCTATGTTTTGAGTAGTCTAAGCTGGCTTCGCGTTAATTGCTAGACGTATTTTGACA
TTCTTTTAGGGGCTTCTTGAGTGGTATACTATAA
