# Supplementary material for: Genome-Wide Identification and Expression Analysis of Tomato ADK Gene Family during Development and Stress
Source: Int J Mol Sci. 2021 Jul 19;22(14):7708. doi: 10.3390/ijms22147708 (PMC8305589; doi:10.3390/ijms22147708)
Supplement: Supplementary file 1 [file ijms-22-07708-s001.zip › Table S2 ADK AA sequences of different plant species.pdf]

Table S2 ADK amino acid sequences of different plant species for phylogenetic tree construction

Arabidopsis (*Arabidopsis thaliana*)

>At5g50370

MATSSAASVDMEDIQTVDLMSELLRRMKCASKPDKRLVFIGPPGSGKGTQSPVIKDEFCL
CHLSTGDMLRAAVAAKTPLGVKAKEAMDKGELVSDDLVVGIMDEAMNRPKCQKGFILD
GFPRTVTQAEKLDEMLNRRGAQIDKVLNFAIDDSVLEERITGRWIHPSSGRSYHTKFAPPK
VPGVDDLTGEPLIQRKDDNADVLRSRLDAFHKQTQPVIDYYAKKENLVNIPAEKAPEEVT
KVVKKVVST

>AT5g63400

MATGGAAADLEDVQTVDLMSELLRRLKCSQKPDKRLIFIGPPGSGKGTQSPVVKDEYCLC
HLSTGDMLRAAVASKTPLGVKAKEAMEKGELVSDDLVVGIIDEAMNKPKCQKGFILDGFP
RTVTQAEKLDEMLKRRGTEIDKVLNFAIDDAILEERITGRWIHPSSGRSYHTKFAPPKTPGV
DDITGEPLIQRKDDNADVLKSRLAAFHSQTQPVIDYYAKKAVLTNIQAEKAPQEVTSEVK
KALS

>AT5g47840

MTGCVNSISPPPVTLYRHRASPSRSSFSLSGDALHSLYRHRRVSRSPSIIAPKFQIVAAEKSEP
LKIMISGAPASGKGTQCELITHKYGLVHISAGDLLRAEIASGSENGRRAKEHMEKGQLVPD
EIVVMMVKDRLSQTDSEQKGWLLDGYPRSASQATALKGFGFQPDLFIVLEVPEEILIERVV
GRRLDPVTGKIYHLKYSPPETEEIAVRLTQRFDDTEEKAKLRLKTHNQNVSDVLSMYDDIT
IKIEGNRSKEEVFAQIDSSLSELLQERNTAPSSLLS

>AT5g35170

MASLSLSSAHFSSTSSSSRSSISTSSLSPSSTSLPLLQSPIRRRYRSLRRRLSFSVIPRRTSRSFS
TSNSQIRCSINEPLKVMISGAPASGKGTQCELIVHKFGLVHISTGDLLRAEVSSGTDIGKRA
KEFMNSGSLVPDEIVIAMVAGRLSREDAKEHGWLLDGFPRSFAQAQSLDKLNVKPDIFILL
DVPDEILIDRCVGRRLDPVTGKIYHIKNYPPESDEIKARLVTRPDDTEEKVKARLQIYKQNS
EAIISAYSDVMVKIDANRPKEVVFEETQTLLSQIQLKRMIKTDKASPVQDKWRGIPTRLNN
IPHSRDIRAYFYEDVLQATIRSIKDGNTRLRVDINIPELNPEMDVYRIGTLMELVQALALSFA
DDGKRVKVCVQGSMGEGALAGMPLQLAGTRKILEYMDWGDDETLGTFVKLGAIGGKE
VDEEDDMFILVAPQNAVGNCIIDDLQAMTTAAGKRPVVLINPRLKDLPASSGIMQTMGRE
QRLEYALTFDNCYVFRLLYYLGTQYPIMGALRMSYPYRYELYKRVNEENGKEKYVLLATY
AERPTPEQIDDAFSGKSRDQSKKASGIWGFLSSVFS

>AT3g01820

MAWLSRVRGVSPVTRLAAIRRSFGSAAALEFDYDSDDEYLYGDDRRLAEPRLGLDGSGP
DRGVQWVLMGAPGAWRHVFAERLSKLLEVPHISMGSLVRQELNPRSSLYKEIASAVNERK
LVPKSVVFALLSKRLEEGYARGETGFILHGIPRTRFQAETLDQIAQIDLVVNLKCSEDHLVN
RNETALPQQEFLGSMLHSPVAINARRESVGVYAQEVEEYYRKQRKLLDFHVGGATSADT
WQGLLAALHLKQVNLTTSQKLTL

>AT2g39270

MAVSHRLLRPATTTIKNTFSSLFIRSLSSSSSGSSLDPKIDLEEAAAQLGKSSSTSTSPYKGRN
FHWVFLGCPGVGKGTYASRLSSLLGVPHIATGDLVREELSSSGLLSSQLKELVNHGKLVPD
EFIISLLSKRLQAGKDKGESGYILDGFPRTVTQAEILEGVTNIDLVINLKLREEALLAKCLGR
RICSECGGNYNVACIDIKGDDDTPRMYMPPLLPPPNCESKLISRADDTEEVVKERLRIYNK
MTQPVEEFYKKRGKLLEFELPGGIPESWARLLRALHLEDDKQSAIA

>AT2g37250
MARLVRVARSSSLFGFGNRFYSTSAEASHASSPSPFLHGGGASRVAPKDRNVQWVFLGCP
GVGKGTYASRLSTLLGVPHIATGDLVREELASSGPLSQKLSEIVNQGKLVSDEIIVDLLSKR
LEAGEARGESGFILDGFPRTMRQAEILGDVTDIDLVVNLKLPEEVLVDKCLGRRTCSQCGK
GFNVAHINLKGENGRPGISMDPLLPPHQCMSKLVTRADDTEEVVKARLRIYNETSQPLEE
YYRTKGKLMEFDLPGGIPESWPRLLEALRLDDYEEKQSVAA
Tomato (*Solanum lycopersicum*)
>SlADK1
MGTVVESANQGAVSLPTNKKVTVIFVLGGPGSGKGTQCANIVEHFGYTHLSAGDLLRAEI
KSGSENGTMISNMIKEGKIVPSEVTVKLLQRAIQENGNDKFLIDGFPRNEENRAAFELVTGI
EPEFVLFFDCPEAEMEKRLLGRNQGREDDNIETIKKRFNVYMESSLPVIEHYNSKGKVRKI
DAVKPVGEVFEAVKAVFAPSNEKVAA
>SlADK2
MAMLSFLGVSARTFLRAASSKSVRAYGSAVAAHFDYDNEEDMEEPSGSVPRRGVQWLIM
GHPMTQRHVYAQWLSKLMDVPYISMGSLVPQQLNPHYNKISSVVNEGKHVPEEVIFGLLS
KRLEEGHCRGENGFILDGIPRTMLQAEILDKVVDIDLVLNLKCSVSKNDRSNGIYSTEDQL
LKRGNLMSSRVMDGGAWKEKQYDHDEQIKPLEEYYRKQKKLLNYQVAGGPAETWQGL
LAALQLQHMMSAVGSTQLTAGC
>SlADK3
MALLSRIRAAAKPLIRTESLRSYGSAAAQLVDYDYDDYEYEEFQNRSCVMEESEGSVPRR
GVQWVIMGDPMAQRHVYAQWLSKLLGVPHISMGSLVRQELHPRSSLYKQIADAVNQGKL
VPEEVIFGLLSKRLEEGYCSGESGFILDGIPRSKIQAEILDKTVDIDLVLNLKRAEDLVSKKD
KSTGLYPPLEFLRMGASGISTSRQPEGGHFRPSSIMEDVSRKNLHVHAEQVNPLEEYYRKQ
RKLLDFQVAGGPGETWQGLLAALHLQHRNAVGSTQLTAGC
>SlADK4
MWRRRFTSLPLFFSHLQQVRRADELKICQAFCTETVKPPVEGESNSGRNSPFVAFVLGGPG
SGKGTQCLKIAETFGFDHIGAGDLLRKEMHSDSENGAMIQKLMKEGSIAPSEVTVKLIKK
AIESAENRKFLIDGFPRSEENRVAYERIIGAEPNFVLFFDCPEEVMVKRVLNRNEGRVDDNE
HTVKERLKVYKAITLPVANHYAMKGKLYKVDGTGTQEEIFERVRPIFASLRLST
>SlADK5
MSTSSVNLEDVPSESLMSELLRRMRCSSKPDKRLILIGPPGSGKGTQSPIIKDEYCLCHLAT
GDMLRAAVAAKTPLGIKAKEAMDKGELVSDDLVVGIIDEALKKPSCQKGFILDGFPRTVV
QAEKLDVMLQNRGTKVDKVLNFAIDDAILEERITGRWIHPASGRSYHTKFAPPKVPGIDDV
TGEPLIQRKDDTAAVLKSRLEAFHRQTEPVIDYYAKKGNVVNLPAEKPPQAVTAEVKKVL
S
>SlADK6
MVVWTRAVVRTWRCRPTNFSRAFSEKLPTSEPKGRNIQWVFLGCPGVGKGTYAARLSKL
LGVPHIATGDLVRQQLSSHGPLASKLVDIVSQGQLISDEIVIDLLSKRLEAGEAKGETGFILD
GFPRTIRQAEILEGVTDIDLVINLKLREDALIAKCLGRRTCSECGGNYNVACIDMKGDDGE
TRMYMPPLLPPPHCETKLITRSDDTENVVKERLRIYHEMSKPVEDFYRQRGKLLEFDLPG
GIPESWSKLLQALNIYDDEDKKSAAA
>SlADK7
MAASLEDVPSESLMSEVLRRLRCSSKPDKRLILIGPPGSGKGTQSPIIKDEYCLCHLATGDM
LRAAVAAKTPLGIKAKEAMNNGELVSDDLVVGIIDEAMKKPSCQKGFILDGFPRTVVQAE

KLDEMLQKQGSKIDKVLNFAIDDAILEERITGRWIHPSSGRSYHTKFQPPKVPGVDDVTGE
PLIQRKDDTAEVLKSRLDAFHRQTEPVINYYSTKGVVASLHAEKPPKEVTSEVKHVLSS
>SlADK8
MASCCSLSFSTVSSKPNKPYSSPISSSLELPFTSQLPFSKKYSLYSNHTLLQTQCRKTQSPDC
PSFLVVGSAKKQEPLRVMISGAPASGKGTQCELITKKYDLVHIAAGDLLRAEIAAGTENGR
RAKEYMDKGQLVPNEIVVTMVKERLMCPDSQEKGWLLDGYPRSLSQAVALKEFQPNLFI
LLEVPEEILVERVVGRRLDPVTGRIYHLKYSPPETDEIAARLTQRFDDTEEKVKLRLHTHR
QNVESVLSMYKDTIFQVDGSVSKEEVFAQIDGALTQLLEAKE
>SlADK9
MDLHKEGDTGSAKQKKVKIVFVIGGPGSGKGTQCKRIAQQFGYTHLSVGEILRQETSSGS
ETGHMVQKIMKEGKLVPSDVTVRLLQQAMQGIDNDKFLIDGFPRDEENVKAFEDLTKME
PEFVLYLDCPQDEMEKRLLSRNEGREDDNIETIRKRLKVFVESTLPTIEYYESKGKIRKVD
AGKSIDEVFESIKVIFSPGKDNKMPPSKHKCKCLIL
>SlADK10
MAAMIRLFRSSSSSSSLISRSLSTAAASETVKSRSYPHSTSVEPKAKSVQWVFLGCPGVGK
GTYASRLSTLLGVPHIATGDLVRDELKSSGPLSKQLAEIVNQGKLVSDEIILNLLSKRLESGE
AKGEAGFILDGFPRTVRQAEILTEVTDIDLVVNLKLPERVLVEKCLGRRICSECGKNFNVAS
IDVAGENGAPRISMAPLNPPSQCISKLITRADDTEAIVKERLSIYWDKSQPVEDFYRSQGKL
LEFDLPGGIPESWPKLLEVLNLDEQEHKLSAAA
>SlADK11
MAMIASVTMNFPHISTHNISSNQTFSPICTNNPSNFSSSSSSSIPISSNSIRLSSSIAYSEQLIAS
HNVNRRTKNRKIKVISARSEPLKVMISGAPASEKDVVGWMKSLYPLQEVSGLSNGSGEIS
GSERFLLQYWSYATQILFSQTNGLIEQQKFVFCGFGLVHISTGDLLRAELSAGTDIGNKAKE
YMNAGRLVPDEIVTAMVTTRLSKEDAKEKGWLLDGYPRTLAQAESLERLNIRPDIYIVLD
VPDAILIDRCVGRRLDPLTGKIYHVTNFPPETEDIKARLITRPDDTEEKVKSRLQIYKQNAE
AILPVYSDIMNKIDGNRGKDSVFAEIDSLLSRVQKEEQDARKSEESAISSTRADMASLSKD
WRGIPTRLNNIPHSREIREYFYTDVLQATQRAVNDGKTRLKIEINIPELNPSMDVYRIGTLM
ELIRVLALSFADDGKRVKVCVQGSMGEGALAGMPLQLAGSRKILEYMDWGDYGALGNFI
NIGSIGGKEVEKQDDVFILVAPQNAVGNCIIDDMRAMTDAAGNRPIILVNPKLKDLPASSGI
MQTMGRDKRLEYAALFEICYQFRLLYYAGTQYPIMGALRMSYPYPYELYKRVDESPGKE
KYISLATFAKRPSIDEMNDAFEGKSRNQEKKAEGFWYVINYPPFYLCFE
Potato (*Solanum tuberosum*)
>Sotub04g013920
MVVWTRAVVRTWRCRPTNFSRAFSEKLPTPEAKGRNVQWVFLGCPGVGKGTYAARLSK
LLGVPHIATGDLVRQQLSSHGPLALKLVDIVSQGQLISDEIVIDLLSKRLEAGEAKGEIGFIL
DGFPRTIRQAEILEGVTDIDLVINLKLREDALIAKCLGRRTCSECGGNYNVACIDMKGDDG
ETRMYMPPLLPPPHCETKLITRSDDTENVVKERLRIYHETSKPVEDFYRKRGKLLEFDLPG
GIPESWQKLLQALNIYDDDEDKKSAAA
>Sotub09g006620
MAAMIRLFRSSSSSSSNSISLISRSLSTAAASETVKSQSYPHNPHSTSVDPKAKTVQWVFLG
CPGVGKGTYASRLSTLLGVPHIATGDLVRDELKSSGPLSKQLAEIVNQGKLVSDEIILNLLS
KRLESGEAKGEAGFILDGFPRTVRQAEILTEVTDIDLVVNLKLPERVLIEKCLGRRICSECG
KNFNVASIDVAGENGAPRISMAPLNPPSQCVSKLITRADDTEAIVKERLSIYWDKSQPVED
FYRSQGKLLEFDLPGGIPESWPKLLEVLNLDEQEYKLSAAA

>Sotub03g005270
MALLSRIRAAVQPLIRTESLRSYGSAAAQLVDYDYDDYEYEEYQNRSYVMEESEGSIPRR
GVQWVIMGDPMAQRHVYAQWLSKLLDVPHISMGSLVRQELHPRSSLYKQIADAVNQGKL
VPEEVIFGLLSKRLEEGYCSGESGFILDGIPRSKIQAEILDKTVDIDLVLNLKCAEDLVSKKD
KSTGLYPPLEFLRRGASGISTSRQPEGGHFRPSSIMDDVSRKNLHVHAEQVNPLEEYYRKQ
RKLLDFQVAGGPGETWQGLLAALHLQHRNAVGSTQLTAGC
>Sotub02g037180
MAMLSFLGVSARPFLLAASSKSVRAYGSAAAAHFDYDYEEEPSGSVPRRGVQWLIMGDP
MTQRHVYAQWLSKLVDVPYISMGSLVRQELNPHYNKISSVVNEGKLVPEEVIFDLLSKRL
EEGYCRGENGFILDGIPRTMFQAIKRLEEYYRKQKKLLNYQVAGGPAETWRGLLAALQLQ
HMMSAVGSTQLTAGC
>Sotub03g023880
MSTSSVNLEDVPSESLMSELLRRMRCSSKPDKRLILIGPPGSGKGTQSPIIKDEYCLCHLAT
GDMLRAAVAAKTPLGIKAKEAMDKGELVSDDLVVGIIDEALKKPSCQKGFILDGFPRTVV
QAEKLDVMLQSRGTKVDKVLNFAIDDAILEERITGRWIHPASGRSYHTKFAPPKAPGIDDV
TGEPLIQRKDDTAAVLKSRLEAFHRQTEPVIDYYAKKGNVVNLPAEKPPQAVTAEVKKVL
S
>Sotub05g016010
MAASLEDVPSESLMSEVLRRLRCSSKPDKRLILIGPPGSGKGTQSPIIKDEYCLCHLATGDM
LRAAVAAKTPLGIKAKESMNNGELVSDDLVVGIIDEAMKKTSCQKGFILDGFPRTVVQAE
KLDEMLQKQGAKIDKVLNFAIDDAILEERITGRWIHPSSGRSYHTKFQPPKVPGVDDVTGE
PLIQRKDDTAEVLKSRLDAFHRQTEPVINYYSTKGVVASLHAEKPPKEVTSEVKNVLSS
>Sotub01g028550
MGTVVESANQGAVSLPTNKKVTVIFVLDLFPLLTWTGGPGSGKGTQCTNIVEHFGYTHLS
AGDLLRAEIKSGSENGTMISNMIKEGKIVPSEVTIKLLQRAIQENGNDKFLIDGFPRNEENR
AAFELVTGIEPEFVLFFDCPEAEMEKRLLGRNQGREDDNIETIRKRFNVYMESSLPVIEYY
NSKGKVRKIDAVKPVGEVFEAVKAVFTPANEKVKY
>Sotub08g022760
MDLHKEGDRGSAKQKKVKIVFVIGGPGSGKGTQCKRIAQQFGYTHLSVGEILRQEISSGS
ETGSMIQKIMKEGKLVPSDVTVRLLQQAMQGINSDKFLIDGFPRNEENVKAFEDLTKMEP
EFVLYLDCPQDEMEKRLLSRNEGREDDNIETIRKRFKVFMESTLPTIEYYESKGKIRKVDA
GKSVDEVFESIKVIFSQGKDNKVPPSRHKCKCLIL
>Sotub11g015570
MASCSSLNFTAVSSNPQKLPSSSISSPIVQRPFTSHLSFSKSSSLHSDQIPIRTHCGKLPQPNG
AGFVVLGCARKKEPLRIMISGAPASGKGTQCELITQKYGLVHIAAGDLLRAEIAAGSENGK
QAKEYMDKGKLVPNEIVVTMVKERLNGPDSREKGWLLDGYPRSSSQAIALEEFGFQPDL
FILLEVPEEILVERVVGRRLDPITGKIYHLKYSPPETEEIASRVTQRFDDTEEKACIVKLRLQ
THHQNVEAILLMYEDITVKVNGIGSKQEVFAQIDGALTQLLEQKQEKLGTVAA
>Sotub06g024300
MASCCSLSFSTVSSKPNKPYSSPISSSLQLPFTSQLPFSKKKSLYSNHTLLQTQCRKTPSPDC
PSFLVVGSAKKQEPLRVMISGAPASGKGTQCELITKKYDLVHIAAGDLLRAEIAAGTENGR
RAKEYMDKGQLVPNEIVVTMVKERLMRPDSQEKGWLLDGYPRSSSQAVALKEFQPDLFI
LLEVPEEILVERVVGRRLDPVTGRIYHLKYSLPETDEIAARLTQRFDDTEEKVKLRLHTHH
QNVESVLSMYKDTIFQVDGSVSKEEVFAQIDAALTQLLEAKE

>Sotub03g020180
MWRRFTSLPLFFSHLQQVRRADELKICQAFCTEIVKPPVEGESNSRRNIPFVAFVLGGPGSG
KGTQCLKIAETFGFDHIGAGDLLRKEIHSDSENGAMIQKLMKEGSIAPSEVTVKLIKKAIES
AENRKFLIDGFPRSEENRVAYERIIGAEPNFVLFFDCPEEVMVKRVLNRNEGRVDDNEHTV
KERLKVYKAITLPVANHYAKKGKLYKVDGTGTQEEIFERVRPIFASLRLST
>Sotub12g007490
MDVYRIGTLMELIRVLALSFADDGKRVKVCVQGSMGEGALAGMPLQLAGSRKILEYMD
WGDYGALGNFVNIGTIGGKEVEKQDDLFILVAPQNAVGNCIIDDMRAMTDAAGNRPIILV
NPKLKDLPASSGIMQTMGRDKRLEYAASFEICYQFRLLYYAGTQYPIMGALRMSYPYPYE
LYKRVDESPGKEKYISLATFAKRPSIDEMNDAFDGKSRNQEKKAEGFWGFLSGIL
Rice (*Oryza sativa*)
>Os12t0236400
MAANLEDVPSMELMTELLRRMKCSSKPDKRVILVGPPGCGKGTQSPLIKDEFCLCHLATG
DMLRAAVAAKTPLGIKAKEAMDKGELVSDDLVVGIIDEAMKKTSCQKGFILDGFPRTVVQ
AQKLDEMLAKQGTKIDKVLNFAIDDAILEERITGRWIHPSSGRSYHTKFAPPKTPGLDDVT
GEPLIQRKDDTAAVLKSRLEAFHVQTKPVIDYYTKKGIVANLHAEKPPKEVTVEVQKALS
>Os11t0312220
MAAAANLEDVPSMDLMNELLRRMKCSSKPDKRLILVGPPGSGKGTQSPIIKDEYCLCHLA
TGDMLRAAVAAKTPLGVKAKEAMDKGELVSDDLVVGIIDEAMKKPSCQKGFILDGFPRT
VVQAQKLDEMLEKKGTKVDKVLNFAIDDSILEERITGRWIHPSSGRSYHTKFAPPKVPGV
DDVTGEPLIQRKDDTAEVLKSRLEAFHKQTEPVIDYYSKKALVANLHAEKPPKEVTAEVQ
KVLS
>Os08t0288200
VHISTGDLLRAEVSSGTEIGKKAKEYMDNGMLVPDQVVTDMVVSRLSQPDVRERGWLL
DGYPRSYAQAQSLESMKIRPDIFIVLEVPDDILIDRCVGRRLDPETGKIYHIKNFPPENDEVS
ARLVTRSDDTFEKVKSRLDTYKQNSEAVIPTYSDLLNQIDGNRQVEVVFNEIDSLLQKICE
NASFNMLAKTNGKPQDSKDTTASKNEFRGIPTRLNNIPHSREIRKYFYNDVLVATRHAVED
KKTRLQIDINIPELNPEMDVYRIGTLMELVRELSLSFADDGKRVKVCVQGSMGQGAFAGIP
LQLAGTRKILEIMDWGEYGAKGTFINFGAVGASEVDKEDDMFILIAPQNAVGNCIIDDMK
AMTDAAGDRPVILVNPRLKDMPGSSGVMQTMGRDMRLKYAASFETCYSFRLLFYAGSFY
PIMGALRMAYPNKYEIYRRVDEPNGQERYVLLEEFVEKPTPDEITNAFRPRKNENEKSASG
FWGFLSGIL
>Os08t0118900
MAGVLRLAGAARSPLARALAPAARRMGASAAAAMEDEAYWTEWEEEEEKARARESAP
VAEMCPTGGGGGGPQWVVMGRPGPQKHAHAARLAEVLAVPYISMGTLVRQELSPASSLY
KKIANSVNEGKLVPEDIIFGLLTKRLEEGYNKGETGFILDGIPRTHMQAEILDEIVDIDLVLN
FKCADNCFMKRRFGGDICPHCGQLFDFSKTASSDRNPSLGSCTWPSQVQHAAVLGLEDSR
MEKMRAYAEQTKLLEDYYRKQRKLMELKTSARPGETWQGLVAALHLQHLDASPTPHKL
TM
>Os08t0109300
MASSMAATATLSPPVLSAERPTVRGGLFLPPSPATSRSLRLQSARRCGISPATRKPRSLPRAA
KVVVAVKADPLKVMIAGAPASGKGTQCELIKSKYGLVHISAGDLLRAEIAAGSENGKRAK
EFMEKGQLVPDEIVVNMVKERLLQPDAQEKGWLLDGYPRSYSQAMALETLNIRPDIFILL
DVPDELLVERVVGRRLDPVTGKIYHLKYSPPENEEIASRLTQRFDDTEEKVKLRLQTHYQN

VESLLSIYEDVIVEVKGDALVDDVFAEIDKQLTSSLDKKTEMVASA
>Os07t0412400
MASRGGGARRTRPNVLVTGTPGTGKTTTCSLLADAVDLRHINIGDLVREKSLHDGWDEEL
ECHIINEDLVCDELEDVMEEGGILVDYHGCDFFPERWFDLVVVLQTDNSILHDRLTSRGYM
GAKLTNNIECEIFQMLLEEARESYKEEIVMPLRSDNVEDISRNVGTLTEWINNWRPSRS
>Os03t0130400
MAAVQRLLRASASGGAAAAAAAARRRMSTAVAPEQTPAAAAFPFAAAAGRARQRVAEER
NVQWVFLGCPGVGKGTYASRLSRLLGVPHIATGDLVRDELASSGPLSVQLAEIVNQGKLV
SDEIIINLLSKRLKKGEEQGESGFILDGFPRTVKQAEILDGVTDIDMVVNLKLREDVLVEKC
LGRRICGQCGKNFNLACIDVKGENGLPPIYMAPLLPPNNCMSKLITRADDTEEVVRNRLQI
YNDMSQPVEGFYRQQGKLLEFDLPGGIPESWPKLLHVLNLEDQEEMKLATA
