# Supplementary material for: Genome-Wide Identification and Expression Analysis of Tomato ADK Gene Family during Development and Stress
Source: Int J Mol Sci. 2021 Jul 19;22(14):7708. doi: 10.3390/ijms22147708 (PMC8305589; doi:10.3390/ijms22147708)
Supplement: Supplementary file 1 [file ijms-22-07708-s001.zip › Table S3 Features of ten motifs of SlADK.pdf]

Table S3 Features and description of ten motifs of SlADKs

Motif	Pfam	Description	Clan
1	AAA_17, ADK	AAA domain, Adenylate kinase	CLOO23
2	ADK	Adenylate kinase	CLOO23
3	ADK	Adenylate kinase	CLOO23
4	ADK	Adenylate kinase	CLOO23
5	ADK_lid	Adenylate kinase,active site lid	n/a
6	AAA_17, ADK	AAA domain, Adenylate kinase	CLOO23
7	/	/	/
8	/	/	/
9	/	/	/
10	/	/	/
