# Supplementary material for: Genome-Wide Identification and Expression Analysis of Tomato ADK Gene Family during Development and Stress
Source: Int J Mol Sci. 2021 Jul 19;22(14):7708. doi: 10.3390/ijms22147708 (PMC8305589; doi:10.3390/ijms22147708)
Supplement: Supplementary file 1 [file ijms-22-07708-s001.zip › Table S5 Primers sequences used for qPCR.pdf]

Table S5 Primer sequences used for quantitative real-time PCR

Name	**Forward primer Sequence (5' →3')**	**Reverse primer Sequence (5' →3')**
SlADK1	CTGAAGCTTATTAATGTGAAC	AGTGACCTTCTTGTTGGTTG
SlADK2	AACATTTCTTCGGGCTGCTTC	GAGTTGTTGAGGTACGAGAG
SlADK3	CATCTTCGCGTCTCTAAAACC	CTGCAGCTGATCCGTAAGAC
SlADK4	ATAGAGTTAATTGGCCTTGGG	CTCCACATCTTCGATCGGTG
SlADK5	ACCATACAAAATTTGCACCTCC	GCATAGTAGTCAATGACCGGCT
SlADK6	CATAGATCCCCAGTCCTCTC	TCCATGTTCTTACCACTGCAC
SlADK7	GTGTTGTAGGGGAAGAAGAAG	ATATCACCGGTGGCCAAATGG
SlADK8	ACTGAGATCTCTATTTAGCAC	TTTGGAGAAGGGTATGATTGG
SlADK9	TACTAATGCGCGAGATTTACC	TAGCTTATATACACTTGCGAT
SlADK10	GAAATCGCCACCCTTTTTCTC	ATATGTACCTTTTCCGACACC
SlADK11	CTCATCATCGTCGTCTTCATC	CATCCATCCCACTACATCCT
SlER5	TATTGGTAAAGATTGGGACATTGA	TGTCTTCTTGTTTGTCACCGTTC
SlCAT3	TCTTACGGTTGGTGCAAGAGGTC	GTTGGCAAGTTTCTCCACCAAATG
SlAPX2	TCAGTGATCCTGCTTTCCGC	TGTCACCACCCTCCCAACTCT
SlGME2	CCATCACATTCCAGGACCAGA	CGTAATCCTCAACCCATCCTTC
