# Supplementary figures and images for: Genome-Wide Identification and Expression Analysis of Tomato ADK Gene Family during Development and Stress
Source: Int J Mol Sci. 2021 Jul 19;22(14):7708. doi: 10.3390/ijms22147708 (PMC8305589; doi:10.3390/ijms22147708)

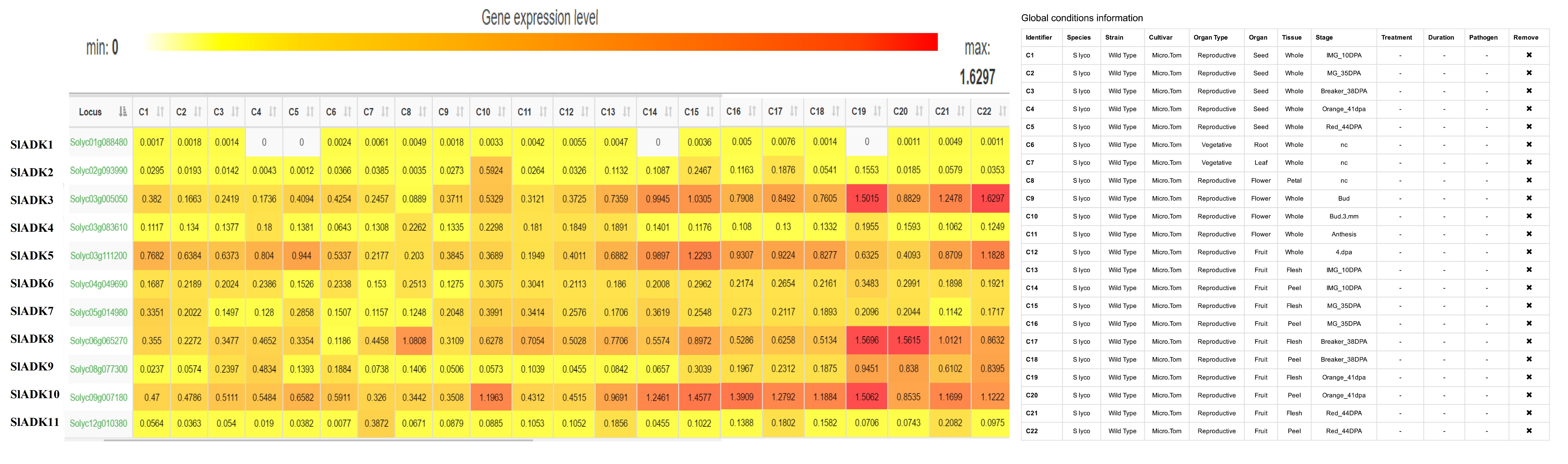

Supplement: Supplementary file 1 [file ijms-22-07708-s001.zip › Figure S1.jpg]

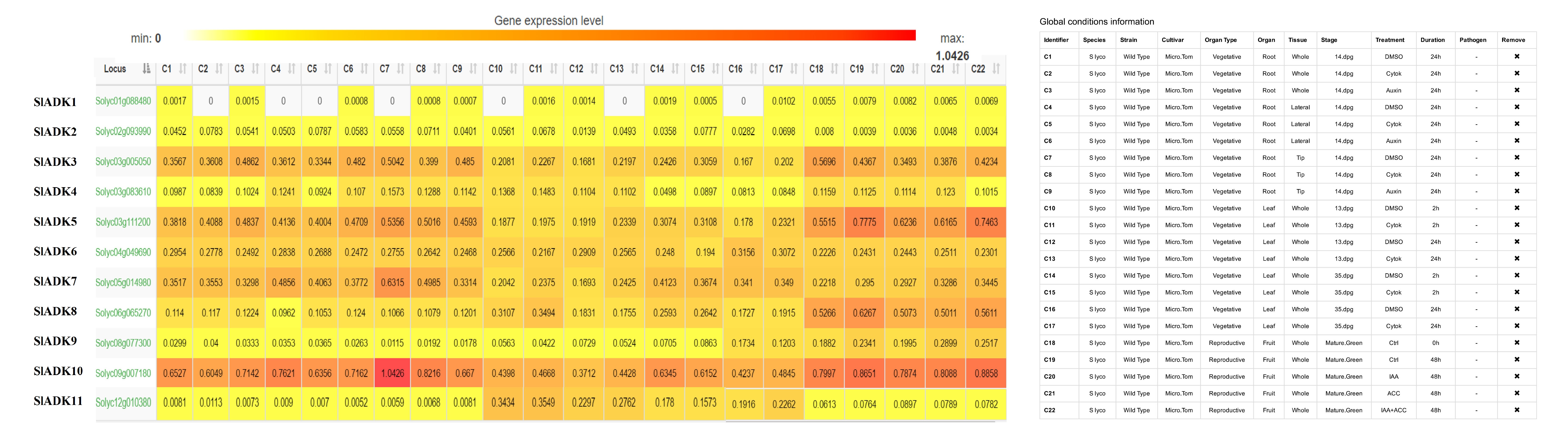

Supplement: Supplementary file 1 [file ijms-22-07708-s001.zip › Figure S2.jpg]

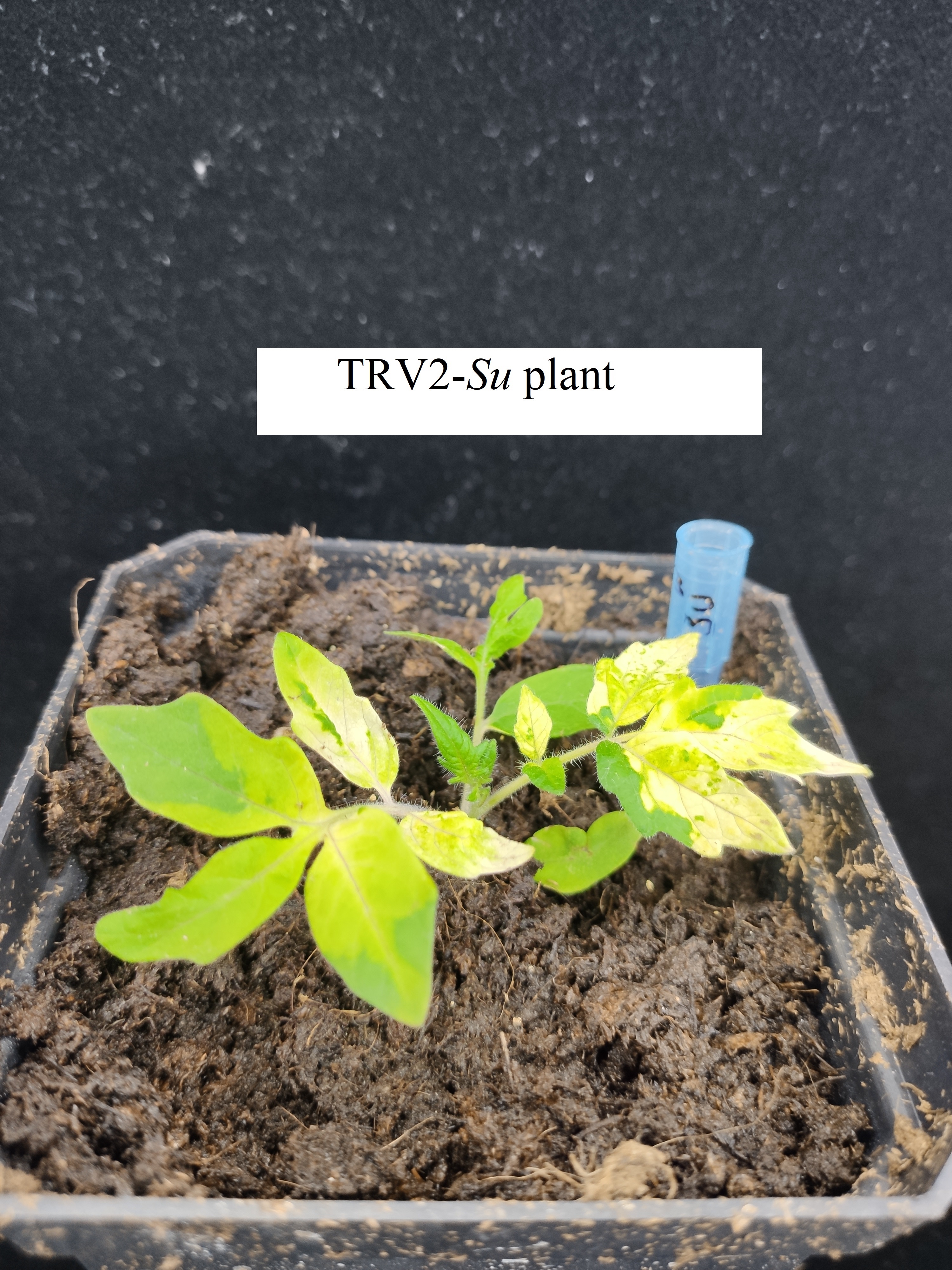

Supplement: Supplementary file 1 [file ijms-22-07708-s001.zip › Figure S3.jpg]
